# Ethnicity, minority status, and inter-group bias: A systematic meta-analysis on fMRI studies

**DOI:** 10.3389/fnhum.2022.1072345

**Published:** 2023-01-06

**Authors:** Aino Saarinen, Liisa Keltikangas-Järvinen, Niklas Ravaja

**Affiliations:** Department of Psychology and Logopedics, Faculty of Medicine, University of Helsinki, Helsinki, Finland

**Keywords:** prejudice, racial bias, in-group bias, ethnic, BOLD, neural

## Abstract

**Introduction:**

This meta-analysis investigated (1) whether ethnic minority and majority members have a neural inter-group bias toward each other, and (2) whether various ethnic groups (i.e., White, Black, and Asian) are processed in the brain differently by the other respective ethnicities.

**Methods:**

A systematic coordinate-based meta-analysis of functional magnetic resonance imaging (fMRI) studies was conducted using Web of Science, PubMed, and PsycINFO (altogether 50 datasets, *n* = 1211, 50.1% female).

**Results:**

We found that ethnic minority members did not show any signs of neural inter-group bias (e.g., no majority-group derogation). Ethnic majority members, in turn, expressed biased responses toward minority (vs. majority) members in frontal, parietal, temporal, and occipital regions that are known to be involved in e.g., facial processing, attention, and perspective-taking. We also found differences in neural response patterns toward different ethnic groups (White, Black, and Asian); broadest biases in neural response patterns were evident toward Black individuals (in non-Black individuals). Heterogeneity was mostly minor or low.

**Discussion:**

Overall, the findings increase understanding of neural processes involved in ethnicity perception and cognition as well as ethnic prejudices and discrimination. This meta-analysis provides explanations for previous behavioral reports on ethnic discrimination toward minority groups.

## 1. Introduction

Ethnic inter-group bias refers to biased mental processing of ethnic in-group and out-group members. It can encompass in-group favoritism or out-group derogation, being either unconscious or conscious (Hewstone et al., 2002; Amodio, 2014). In this way, inter-group bias is an adjacent concept to prejudices that are negative evaluations or emotional reactions toward an out-group member on the basis of preconceptions (Amodio, 2014). Inter-group bias has enormous societal significance by providing a psychological foundation for ethnic discrimination (Greenwald and Pettigrew, 2014). As the harmful impacts of ethnic discrimination are well-known (Ben et al., 2017; Benner et al., 2018), the European Union Charter of Fundamental rights has pointed out that “any discrimination based on any ground such as — race, color, ethnic or social origin — shall be prohibited.” Further, the United Nations Commission on Human Rights (1998) has emphasized that international community must “protect effectively the human rights of all persons belonging to national or ethnic — minorities without any discrimination and in full equality.”

Despite discrimination-reduction efforts, ethnic discrimination has remained evident in the Organization for Economic Co-operation and Development (OECD) countries in 1990–2015 (Zschirnt and Ruedin, 2016). A systematically replicated key finding has been that discrimination is not equally distributed among or toward all ethnic groups. In particular, Black and Hispanic employees are more likely to perceive racial discrimination than White employees (Avery et al., 2008), and teachers have less positive expectations for African American than European American students (Tenenbaum and Ruck, 2007). Not only individual’s ethnic group as such, but also individual’s ethnic minority or majority status (i.e., the proportion of one’s ethnic group in one’s living region) plays a crucial role. When compared to ethnic majorities, ethnic minorities have substantially higher odds of encountering discrimination (Avery et al., 2008), they need to send ca. 50% more applications to get invited to a job interview (Zschirnt and Ruedin, 2016), and they have higher risk of being charged or fully prosecuted (Wu, 2016). Taken together, the current evidence indicates that certain ethnic groups and ethnic minorities are particularly susceptible to receiving negatively biased responses from other ethnicities.

This evidence has remained ignored, however, in the neuroscientific models of ethnic inter-group bias and prejudices. That is, the neuroscientific models on the topic have postulated a single model of neural responses toward ethnic in- and out-groups, without paying attention to the specific ethnic group in question, or ethnic minority or majority status (Amodio, 2014; Molenberghs and Louis, 2018). Thus, there is an urgent need to gain evidence whether distinct or overlapping brain regions are involved in inter-group bias among ethnic minority vs. majority members, and whether similar or different brain regions are involved in response to different ethnic groups. This was the aim of the present meta-analysis.

To date, two meta-analyses have examined ethnic inter-group biases in general, without taking into consideration possible differences between ethnic groups or between ethnic minorities and majorities. The meta-analyses showed that ethnic inter-group bias associates with activity patterns in the frontal cortex, insula, right superior temporal gyrus, left superior parietal gyrus, and cerebellum (Merritt et al., 2021; Saarinen et al., 2021). The findings were interpreted to reflect biases in attentional, perspective-taking, and emotional-prosodic processes toward ethnic out-groups (Merritt et al., 2021; Saarinen et al., 2021). Although the meta-analyses provided crucially important pieces of neural evidence on ethnic inter-group bias, it was acknowledged that “neural responding may vary depending on the specific racial groups involved (e.g., Black vs. White and Asian vs. Black)” and that “distinguishing among various types of cross-race dyads is an important future direction” (Merritt et al., 2021).

We conducted a systematic literature search and meta-analysis of functional magnetic resonance imaging (fMRI) studies to investigate blood-oxygenation-level-dependent (BOLD) responses to visually presented ethnic groups in healthy adults. In this study, we used the concept of “ethnic group” to refer a group of individuals sharing distinctive outward physical characteristics (such as skin color or hair texture). More specifically, we investigated (1) whether ethnic minority and majority members have a neural inter-group bias toward each other and (2) whether various ethnic groups (i.e., White, Black, Asian) are processed in a biased way by the other ethnicities.

## 2. Materials and methods

### 2.1. Literature search

The Meta-analyses Of Observational Studies in Epidemiology (MOOSE) Checklist was followed throughout the meta-analysis. Moreover, most recent recommendations for a neuroimaging meta-analysis were followed through the process (when applicable) (Müller et al., 2018). The literature search was conducted using PsycINFO, PubMed, and Web of Science (17 January 2022). The search was directed to the fields of title and abstract, and we set no restrictions regarding publication date, language, number of citations, or publication status (see Supplementary methods for the search terms).

**FIGURE 1:**
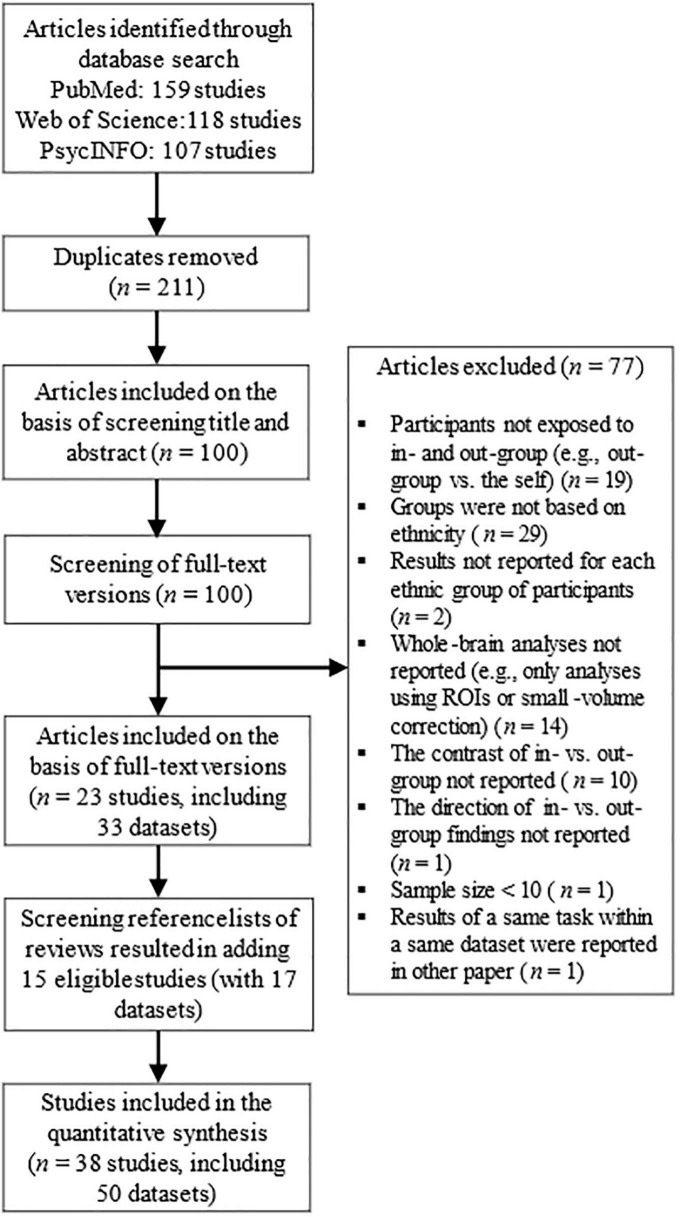
Literature search process.

A PRISMA flowchart on the article selection process can be found in Figure 1. After removing duplicates, all identified studies were screened on the basis of title and abstract and classified as eligible/ineligible for this meta-analysis. Thereafter, the eligible full-text articles were screened more precisely on the basis of the exclusion and inclusion criteria. Besides of original studies, the reference lists of all meta-analyses and reviews (identified by the search terms) were manually checked for any additional eligible studies. In case some necessary information was missing, the authors of the original studies were contacted (Supplementary Table 4 describes the studies about which we received additional details from the authors). Included studies are listed and described in Supplementary Tables 1, 2. Literature search was conducted by AS (Ph.D in psychology; Ph.D in medicine; and Ph.D in educational sciences). In order to increase transparency of the literature search, we have provided additional descriptive information of the included studies in Supplementary Table 3; the statistical contrasts selected from each study in Supplementary Table 4; and the primary reasons for excluding articles in Supplementary Table 5 (exclusion on the basis of title and abstract) and in Supplementary Table 6 (exclusion on the basis of full-text version). Further, the full statistical data (e.g., all the coordinates and statistical estimates) can be requested from the corresponding author.

### 2.2. Inclusion and exclusion criteria

We had the following inclusion criteria: an original peer-reviewed fMRI article; *n* ≥ 10; adult sample (mean age > 18 years); a non-clinical sample (the subjects did not have any reported diseases or medications); a study design including exposure to ethnic in-group and out-group; a stimulus material including visual exposure to ethnic in-group and out-group members, including their faces (so that skin color could be approximately obtained); study design included face visual material of ethnic in-group and out-group (regardless whether the study topic was in/out-group favoritism/degoration or other ethnic perceptual or cognitive processing); the coordinates had been reported in the Talairach Atlas (Tal) or the Montreal Neurological Institute (MNI) space; *T* or *Z* statistics or *p* values of the observed BOLD response toward ethnic in-group vs. out-group members were available; the study reported results of whole-brain analyses or analyses with very limited masking. More precisely, we did not include small-volume corrected results or region-of-interest (ROI) based results because a previous review recommended to only include studies with whole-brain results available (Radua and Mataix-Cols, 2012). We allowed masking if the study had proved that masking did not limit regions where significant in-group vs. out-group differences could be obtained; if masking was limited to regions (e.g., the brain stem or occipital lobe) that are not theoretically relevant in the context of inter-group bias; or if masking was limited to such brain regions that, in preliminary analyses, had been shown to correlate with the mental process under investigation (e.g., in-group vs. out-group contrast during facial processing was investigated in regions that were significant in face vs. non-face contrast). The exclusion criteria (mostly consisting of the opposite criteria to the inclusion criteria) can be found in Supplementary methods.

Data extraction is described in Supplementary methods.

### 2.3. Meta-analyses

The meta-analyses were conducted using the Seed-based *d* Mapping (SDM) software (version 6.21). Further meta-analytical details of the SDM software are available elsewhere (Radua et al., 2012, 2014; Albajes-Eizagirre et al., 2019).

During the data pre-processing, the lower and upper bounds of the possible effect-size values of the studies were estimated (full anisotropy = 1.0, full width at half maximum of Gaussian kernel = 20 mm, voxel size = 2 mm). Next, a mean analysis was conducted, representing the weighted mean difference in the fMRI activity (i.e., BOLD responses) toward in-group vs. out-group. There is evidence that sufficient statistical stability can be reached with 20 imputations (Radua et al., 2012). To be on the safe side, we used 50 imputations. The statistical threshold consisted of an uncorrected voxel *p* value <0.005, cluster extent ≥10 voxels, and *SDM-Z* < 1, in accordance with previous recommendations (Radua et al., 2012). Regarding other analytical details, we used the default settings of the SDM software that are described in other sources more precisely (Radua et al., 2012, 2014; Albajes-Eizagirre et al., 2019). All the meta-analyses were adjusted for age and gender.

#### 2.3.1. Main analysis

First, a main analysis was conducted where all the original datasets were included (regardless of ethnic minority/majority status or ethnic in-group). Supplementary Table 4 presents the contrasts that were selected from each study. In this analysis, we contrasted the BOLD responses to ethnic in-group vs. out-group in all the original samples.

#### 2.3.2. Sub-group analyses separately in subjects with ethnic minority or majority status

Next, we examined neural inter-group bias separately (a) in subjects belonging to ethnic minorities (responses to ethnic minorities vs. majorities) and (b) in subjects belonging to ethnic majorities (responses to ethnic minorities vs. majorities). The analyses were adjusted for age and gender. Supplementary Table 1 describes the studies included in these analyses; and Supplementary Table 3 provides additional information on subjects’ ethnic background.

When classifying subjects’ ethnic groups, we used similar classifications to those in the original studies: (1) Caucasian/White/European-American, (2) African/African-American/Black, or (3) Asian/Chinese/Korean. Subjects’ ethnic majority or minority status was defined by cross-tabulating (1) subjects’ ethnicity and (2) most recent statistical data available on ethnic minorities and majorities in the study country (e.g., the racial distribution in the US was checked from this source: https://www.kff.org/other/state-indicator/distribution-by-raceethnicity/). In general, the study countries had obvious minority/majority groups; most common study countries were the US (ca. 76% White, 14% Black, 7% other ethnic groups) and China (ca. 91% Han Chinese, 9% other ethnic groups). The study country was defined on the basis of the following information: the region where the subjects were collected, the location of the institutional board for ethical permission, the location of the fMRI imaging device, or (if none of the previous details were available) from the country and region of the authors’ affiliations. We excluded a study in case it included an ethnically heterogeneous sample (e.g., including both White, Black, and Asian subjects) and the results had not been reported separately within each ethnic group.

Then, we conducted a sub-group meta-analysis *in ethnic majority members*. Specifically, we analyzed the contrast of BOLD responses to ethnic majority members (i.e., subjects’ ethnic in-group, individuals coming from a same majority group) vs. BOLD responses to ethnic minority members (i.e., subjects’ ethnic out-group, individuals coming from a minority that the study subjects did not belong to). Second, we conducted a corresponding sub-group analysis *in ethnic minority members*. That is, we analyzed the contrast of BOLD responses to ethnic minority members (i.e., subjects’ ethnic in-group, individuals coming from a same minority than the study subjects) vs. BOLD responses to ethnic majority members (i.e., subjects’ ethnic out-group, individuals coming from a majority that the study subjects did not belong to). In these analyses, we excluded studies that compared exposure to two different types of ethnic minorities (e.g., exposure to African-American vs. Japanese individuals in a sample living in the US). Supplementary Table 4 presents the contrasts that were selected from each study.

#### 2.3.3. Sub-group analyses separately toward White, Black, and Asian target individuals

Here, we examined whether various ethnic groups (i.e., White, Black, Asian) are processed in a biased way by the other ethnicities. The analyses were adjusted for age and gender. Supplementary Table 2 presents the studies included in these analyses.

(1) When examining inter-group bias toward White target individuals (i.e., White ethnicity constituted subjects’ ethnic out-group), we included subjects with non-White ethnicity (i.e., subjects’ ethnic in-group was Asian or Black ethnicity). Specifically, we contrasted the BOLD responses to White vs. non-White target individuals. (2) When examining inter-group bias toward Black target individuals (i.e., Black ethnicity composed subjects’ ethnic out-group), we included subjects with non-Black ethnicity (i.e., subjects’ ethnic in-group was Asian or White ethnicity). In this analysis, we contrasted the BOLD responses toward Black vs. non-Black target individuals. (3) When examining inter-group bias toward Asian target persons (i.e., Asian ethnicity composed subjects’ ethnic out-group), we included subjects with non-Asian ethnicity (i.e., subjects’ ethnic in-group was Black or White ethnicity). More specifically, we contrasted the BOLD responses toward Asian vs. non-Asian target individuals. The analyses were controlled for age and gender.

#### 2.3.4. Heterogeneity and publication bias of the findings

In order to investigate robustness of the results, heterogeneity was assessed using *I*^2^ statistics (i.e., the percentage of total variance between studies resulting from rather a heterogeneity than chance). We used the following interpretations of *I*^2^ values: low (<25%), moderate (>50%), and high (>75%). Additionally, the amount of publication bias was evaluated with the metabias tests (Radua and Albajes-Eizagirre, 2019) and a graphical investigation of funnel plots.

## 3. Results

### 3.1. Search results and descriptive information of the included studies

The literature search process is illustrated in Figure 1. The literature search resulted in a total of 50 eligible original datasets that had been published between 2008–2021 and included altogether 1,211 subjects (*M*_age_ = 24.5 years, 50.1% female). There were 39 datasets with samples including majority members (*n* = 945, *M*_age_ = 24.1, 48.9% female, a total of 411 brain loci with significant differences in BOLD responses toward in- vs. out-group in the original studies); and 11 datasets with samples including minority members (*n* = 246, *M*_age_ = 26.2, 54.6% female, 51 loci). Additionally, we found altogether 21 datasets with White target individuals (*n* = 477, *M*_age_ = 23.8, 49.0% female, 89 loci); 21 datasets with Black target individuals (*n* = 557, *M*_age_ = 26.4, 48.0% female, 297 loci); and 8 datasets with Asian target individuals (*n* = 204, *M*_age_ = 32.8, 57.2% female, 89 loci). Most minority members were newly moved immigrants: they had been living only a short time in the country, and many of them were students of an abroad study program or had not had close contacts with the ethnic out-group before the study (see Supplementary Table 5).

Further details about the included studies can be found in the Supplementary material. Supplementary Table 1 presents studies categorized on the basis of subjects’ ethnic majority vs. minority status; Supplementary Table 2 presents studies categorized on the basis of target individuals’ ethnicity; and Supplementary Table 3 includes additional descriptive information on subjects’ ethnic background.

### 3.2. Ethnic in-group bias: meta-analysis on all the eligible studies

First, we conducted a meta-analysis including all eligible studies. There was higher activity toward ethnic in-group in the left superior parietal gyrus (*x* = −22, *y* = −78, *z* = 46, *SDM-Z* = 3.263, *p* = 0.00055) and left median network (*x* = −6, *y* = −48, *z* = 28, *SDM-Z* = 2.787, *p* = 0.00266) when compared to ethnic out-group. No brain region exhibited the opposite activity pattern. There was not significant publication bias, as indicated by a visual inspection of the funnel plots (see Supplementary Figure 1) and the results of metabias tests (*p* = 0.998–0.994 for the peak voxels). Heterogeneity was minor in the left median network (*I*^2^ = 0.256) and low in the left superior parietal gyrus (*I*^2^ = 10.034).

### 3.3. Meta-analyses on inter-group bias among ethnic minority and majority members

#### 3.3.1. Meta-analysis in ethnic majority members

**FIGURE 2:**
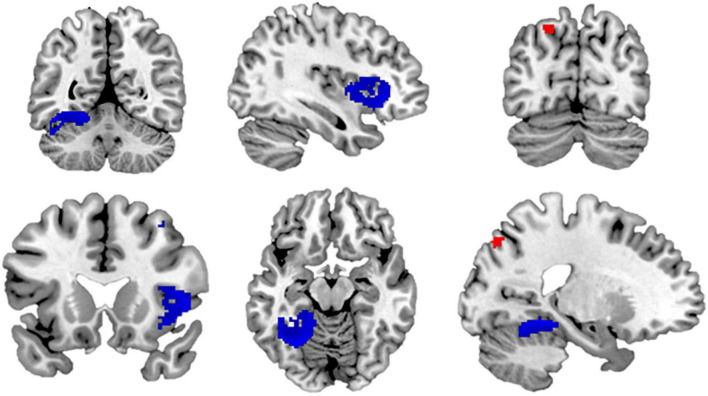
The results of the meta-analysis among ethnic majority members. The brain regions with higher activity toward ethnic majority (vs. minority) members are marked with red color; and the brain regions with higher activity toward ethnic minority (vs. majority) members are marked with blue color.

Next, we conducted a meta-analysis on inter-group bias among ethnic majority members. Ethnic majority members showed higher activity toward ethnic majority members (vs. minority members) in the left superior parietal gyrus and in an undefined brain region (Brodmann area no. 30) (Table 1a and Figure 2). Additionally, ethnic majority members had higher activity toward ethnic minority members (vs. majority members) in the right inferior frontal gyrus, left fusiform gyrus, right cuneus cortex, and right inferior temporal gyrus (Table 1a and Figure 2).

**TABLE 1:** Neural inter-group bias toward ethnic minority vs. majority members (a) among ethnic majority members and (b) among ethnic minority members.

MNI coordinates	*SDM-Z*	*p*	Voxels	Description	Direction of the contrast
**(a) Among ethnic majority members**					
−20, −76, 44	2.999	0.001356363	35	Left superior parietal gyrus, BA 7	Ethnic majority > ethnic minority
18, −28, −20	2.714	0.003326476	11	(undefined), BA 30	Ethnic majority > ethnic minority
50, 22, 4	−3.888	0.000050426	1,172	Right inferior frontal gyrus, triangular part, BA 45	Ethnic minority > ethnic majority
−30, −64, −16	−3.810	0.000069559	922	Left fusiform gyrus, BA 19	Ethnic minority > ethnic majority
22, −96, 10	−2.964	0.001518071	42	Right cuneus cortex, BA 18	Ethnic minority > ethnic majority
54, −58, −10	−3.393	0.000345945	13	Right inferior temporal gyrus, BA 37	Ethnic minority > ethnic majority
**(b) Among ethnic minority members**					
No significant results					

Heterogeneity was minor or low in all the peak voxels (*I*^2^ = 1.565–23.042). Further, the metabias tests did not obtain any statistically significant publication bias (*p* = 0.995–1.000 for the peak voxels). The funnel plots are available in Supplementary Figure 2.

#### 3.3.2. Meta-analysis in ethnic minority members

Next, we examined inter-group bias among ethnic minority members (toward ethnic minority vs. majority members). No brain region showed higher activity toward ethnic minority (vs. majority) members, or vice versa. That is, we did not obtain any neural inter-group bias among ethnic minority members.

### 3.4. Meta-analyses on inter-group bias toward different ethnic groups

#### 3.4.1. Ethnic inter-group bias toward White (vs. non-White) target individuals

Then, we examined whether various ethnic groups (i.e., White, Black, Asian) are processed in a biased way by the other ethnicities.

**FIGURE 3:**
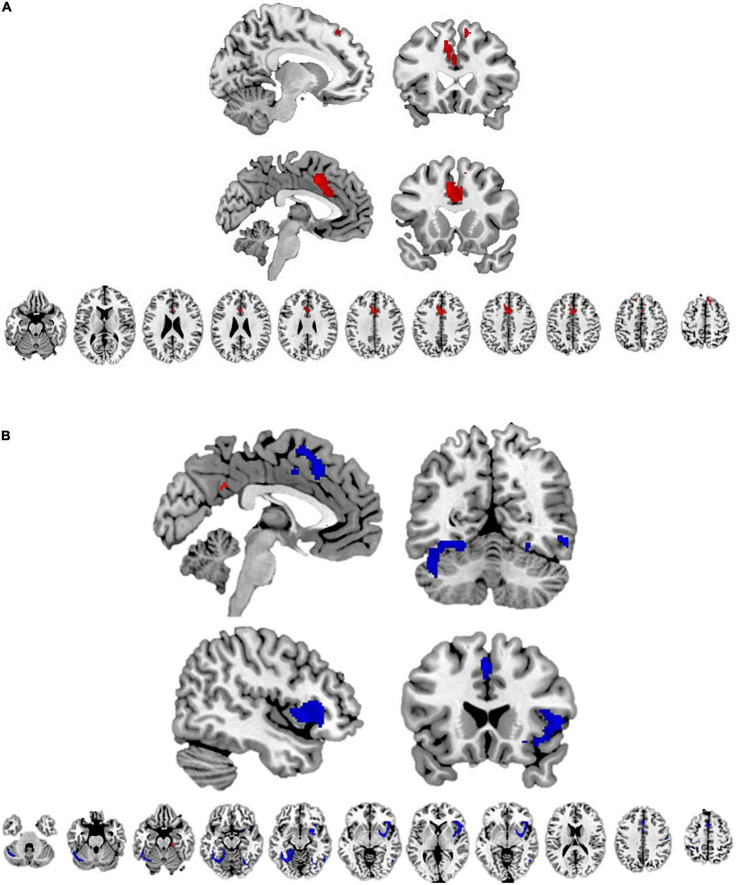
**(A)** The brain regions with lower activity toward White (vs. non-White) target individuals (marked with red color). **(B)** The brain regions with lower activity toward Black (vs. non-Black) target individuals are marked with red color; and the brain regions with higher activity toward Black (vs. non-Black) target individuals are marked with blue color.

First, we conducted a meta-analysis on neural inter-group bias toward White (vs. non-White) target individuals in non-White study subjects (Black or Asian subjects). The findings are presented in Table 2a. We found lower activity toward White (than non-White) individuals in the left supplementary motor area, and right and left superior frontal gyri in non-White subjects (Table 2a and Figure 3A). No brain region showed the opposite activity pattern (Table 2a). Heterogeneity was low in the left supplementary motor area (*I*^2^ = 10.613) and moderate in the right and left superior frontal gyri (*I*^2^ = 28.315 and *I*^2^ = 25.026, respectively). We found no significant publication bias in the funnel plots or metabias tests (*p* = 0.978–0.992 for the peak voxels) (see Supplementary Figure 2).

**TABLE 2:** Neural inter-group bias (a) toward White (vs. non-White) target individuals, (b) toward Black (vs. non-Black) target individuals, and (c) toward Asian (vs. non-Asian) target individuals.

MNI coordinates	*SDM-Z*	*p*	Voxels	Description	Direction of the contrast
(a) Toward White (vs. non-White) target individuals					
2, 10, 44	3.943	0.000040233	512	Left supplementary motor area, BA 32	White < non-White
10, 26, 56	3.375	0.000369251	59	Right superior frontal gyrus, medial, BA 8	White < non-White
−10, 36, 54	2.834	0.002301991	16	Left superior frontal gyrus, medial, BA 9	White < non-White
**(b) Toward Black (vs. non-Black) target individuals**					
−10, 8, 6	2.874	0.002026737	22	Left anterior thalamic projections	Black < non-Black
−4, −48, 30	2.905	0.001836419	21	Left posterior cingulate gyrus, BA 23	Black < non-Black
20, −26, −20	2.936	0.001664579	19	Right parahippocampal gyrus, BA 30	Black < non-Black
48, 16, −2	−4.216	0.000012398	1019	Right insula, BA 47	Black > non-Black
−20, −46, −10	−4.108	0.000019968	777	Left lingual gyrus, BA 30	Black > non-Black
−2, 22, 42	−3.870	0.000054300	206	Left superior frontal gyrus, medial, BA 32	Black > non-Black
30, −68, −14	−3.635	0.000139177	73	Right fusiform gyrus, BA 19	Black > non-Black
54, −60, −8	−3.956	0.000038028	64	Right inferior temporal gyrus, BA 37	Black > non-Black
54, −12, 36	−3.452	0.000277758	44	Right postcentral gyrus, BA 3	Black > non-Black
−40, −42, 48	−3.104	0.000953972	31	Left inferior parietal (excluding supramarginal and angular) gyri, BA 40	Black > non-Black
0, 4, 40	−3.262	0.000553787	22	Left median cingulate/paracingulate gyri, BA 24	Black > non-Black
−36, −82, 14	−3.069	0.001073062	20	Left middle occipital gyrus, BA 19	Black > non-Black
32, −80, −8	−3.163	0.000780761	12	Right inferior network, inferior longitudinal fasciculus	Black > non-Black
**(c) Toward Asian (vs. non-Asian) target individuals**					
No significant results					

In analysis (a), participants were Asian in 17 studies and Black in four studies. In analysis (b), participants were Black in 20 studies and Asian in one study. In analysis (c), participants were White in all the studies.

#### 3.4.2. Ethnic inter-group bias toward Black (vs. non-Black) target individuals

Next, we conducted a meta-analysis on neural inter-group bias toward Black (vs. non-Black) target individuals in non-Black study subjects (White or Asian subjects). There was lower activity toward Black (vs. non-Black) target individuals in the left anterior thalamic projections, left posterior cingulate gyrus, and right parahippocampal gyrus (Table 2b). In addition, we found higher activity toward Black (vs. non-Black) target individuals in numerous brain regions, with largest clusters (>100 voxels) appearing in the right insula, left lingual gyrus, and left superior frontal gyrus (Table 2b). The findings are illustrated in Figure 3B.

Heterogeneity was minor in all the peak voxels (*I*^2^ = 0.535–11.377), except for the peak voxel of the left anterior thalamic projections where we obtained slightly higher but still low heterogeneity (*I^2^* = 23.728). The metabias tests did not obtain any significant publication bias (*p* = 0.989–0.998 for all the peak voxels). The funnel plots are available in Supplementary Figure 3.

#### 3.4.3. Ethnic inter-group bias toward Asian (vs. non-Asian) target individuals

Finally, we conducted a meta-analysis on neural inter-group bias toward Asian (vs. non-Asian) target individuals in non-Asian study subjects (Black or White subjects). No brain region showed higher activity toward Asian (vs. non-Asian) target individuals, or vice versa. That is, we did not obtain any neural inter-group bias toward Asian (vs. non-Asian) target individuals.

## 4. Discussion

### 4.1. A summary of the main findings

This meta-analysis provided a novel perspective to the current literature by investigating the neural basis of ethnic inter-group bias (a) separately among ethnic minority and majority members (toward visually presented ethnic minority vs. majority members) and (b) separately toward visually presented Black, White, and Asian individuals. The pooled evidence from the original studies showed that, first, neural inter-group bias was non-evident in ethnic minority members but evident in ethnic majority members. Second, we found neural inter-group bias toward Black and White individuals but not toward Asian individuals in subjects not belonging to the respective ethnic group. Finally, heterogeneity was mostly minor or low in the brain regions related to inter-group bias, and the metabias tests did not identify any significant publication bias. In summary, this study showed that neural inter-group bias is differently expressed among ethnic minority vs. majority members, and toward different ethnic groups (White, Black, and Asian individuals).

### 4.2. Neural inter-group bias in ethnic majority and minority members

We did not find any neural inter-group bias in ethnic minority members: ethnic minority members did not show different responses to ethnic minority vs. majority members. Hence, in minority members, we obtained neither signs of out-group derogation (such as deactivation of perspective-taking-related brain regions when observing majority members); nor signs of anxiety or threat responses toward ethnic majority members. In the original studies, most minority members were newly moved immigrants: they had been living only a short time in the country, and many of them had not had close contacts with the ethnic out-group beforehand (see Supplementary Table 5). It is possible that inter-group bias could be evident after spending a longer time period in a country as an ethnic minority member. Finally, it is necessary to consider that we had a smaller number of minority (than majority) members in this meta-analysis, providing a further possible explanation for the null results.

The fusiform gyrus showed more activity toward ethnic minority (vs. majority) members; and also toward Black (vs. non-Black) individuals. The fusiform gyrus constitutes a face-specialized region (Haxby et al., 2000), shows activity in response to visual objects of expertise (Wong et al., 2009), and responds sensitively to face color (Nakajima et al., 2014). There is evidence from experimental studies and computational models that facial features, including ethnicity and race, are stored through previous experience in a multidimensional memory space that guides face perception (Valentine, 1991; Caldara and Hervé, 2006): ethnic majority members may have developed “a field of expertise” of processing face of their own ethnic group. Related to this, we found that the left lingual gyrus showed more activity toward Black (vs. non-Black) individuals. The left lingual gyrus is involved in working memory for schematic faces (Kozlovskiy et al., 2014), responds more strongly to faces than non-faces (Patriquin et al., 2016), and exhibits higher reactivity to submissive than neutral faces (Chiao et al., 2008). This provides further evidence of biased responses to Black faces, and also arouses a cautious question whether Black faces are more easily interpreted as submissive than non-Black faces.

Our meta-analysis showed higher activity toward ethnic minority (vs. majority) members in the right inferior frontal gyrus; and also higher activity toward Black (vs. non-Black) individuals in the left median cingulate cortex. The right inferior frontal gyrus is a region of the emotional empathy network (Schulte-Ruther et al., 2007; Jabbi and Keysers, 2008; Seitz et al., 2008; Perry et al., 2012; Peled-Avron et al., 2019; Li et al., 2021), and is also involved in executive functioning such as inhibitory control and attentional monitoring according to original studies (Aron et al., 2003, 2014; Hampshire et al., 2010; Gavazzi et al., 2017) and meta-analyses (Simmonds et al., 2008; Gavazzi et al., 2021). The left median cingulate cortex is a part of the mirror neuron system and involved in prosocial emotions (Amodio, 2014; Molenberghs and Louis, 2018). Consequently, these findings would support an interpretation that empathic responses might be stronger toward minority (vs. majority) members and toward Black (vs. non-Black) individuals.

Our results also showed, however, that observing Black (vs. non-Black) individuals correlates with higher activity of the right insula, which is commonly interpreted as a stronger disgust reaction (Amodio, 2014). Additionally, we found higher activity toward minority members (vs. majority) members and toward Black (vs. non-Black) individuals in the right inferior temporal gyrus, which is found to show activation when one overestimates his/her capacity to express empathy for others (Sollberger et al., 2014). This implies that majority members and non-Black (White or Asian) individuals may be prone to overestimate their empathic expressions toward minority members and Black individuals. Taken together, a very cautious interpretation might be that, toward Black and minority members, there may be a kind of spontaneous disgust reaction that may be compensated by activating the frontal empathy-related regions and an attempt to express empathy toward them.

Finally, we did not find any inter-group bias in the subcortical regions (e.g., the amygdala), indicating that there may not be any threat-related reactions when observing ethnic majority or minority members. We found, however, that ethnic majority members showed higher activity toward ethnic majority (than minority) members in the left superior parietal gyrus. It is a part of the dorsal frontoparietal network that is thought to maintain a “salience map” and direct attention to such external stimuli that are salient on the basis of semantic knowledge (Corbetta and Shulman, 2002). Thus, a cautious interpretation might be that, when ethnic majority members have directed attention to majority members (compared to minority members), they may be more actively retaining semantic knowledge from their memory.

### 4.3. Neural intergroup bias toward Asian, Black, and White target individuals

The left superior frontal gyrus showed lower activity toward White (vs. non-White) individuals; and also higher activity toward Black (vs. non-Black) individuals. Thus, the left superior frontal gyrus seemed to respond in opposite directions depending on the target individuals’ ethnicity. In general, this brain region is thought to be involved in working memory, especially during high cognitive load and spatial processing (du Boisgueheneuc et al., 2006). Interestingly, higher activity of the left superior frontal gyrus also associates with social semantics (Wood et al., 2003), stronger feelings of social punishment (Zinchenko, 2019), and higher social dominance in response to disgusting facial expressions (Aleman and Swart, 2008). Thus, our finding possibly implies that a non-Black observer may have stronger feelings of social punishment or disgust-related dominance when observing a Black individual, while those feelings may be less evident when observing a White individual.

We found biased responses toward Black (vs. non-Black) individuals in the middle occipital gyrus that is involved in perception of visuospatial objects (Yang et al., 2015), and in the postcentral gyrus is known to include the primary somatosensory cortex. Thus, both regions contribute to primary sensory processing. Interestingly, we also found biased reactivity to Black (vs. non-Black) individuals in the left anterior thalamic projections that constitute a link between the primary sensory regions and frontal cortex (where we also found biased responses) (Mitchell, 2015). The thalamocortical projections play a role in sensory selection during divided attention (Wimmer et al., 2015) and in maintaining frontal activity during working memory performance (Bolkan et al., 2017). Hence, there seems to be biases in the neural mechanisms regulating attentional resources toward different ethnic groups. This attention-related interpretation was supported but that we also found lower activity toward Black (vs. non-Black) individuals in the left posterior cingulate gyrus. This region typically deactivates when concentrating on an external task and when sustaining a vigilant attentional state (Leech and Sharp, 2014).

There was lower activity toward White (vs. non-White) individuals in the left supplementary motor area (SMA). The SMA is involved in the preparation and execution of voluntary movements (Weilke et al., 2001). Thus, our finding may reflect biased motor responses, as most fMRI tasks of the original studies included pressing a button in response to ethnic groups. Additionally, the SMA is also in self-other distinction and empathic perspective-taking (Schulte-Ruther et al., 2007). Thus, an alternative interpretation of our finding might be that White (vs. non-White) target individuals may arouse lower perspective-taking responses.

We found no signs of neural inter-group bias toward Asian (vs. non-Asian) individuals in subjects with non-Asian ethnicities. This is in accordance with some behavioral studies showing that Asian individuals encounter less racial discrimination in the UK labour market when compared to e.g., African individuals (Heath and Di Stasio, 2019); and Asian-Indians are more likely to encounter benevolent than hostile prejudice in the US (Ramasubramanian and Oliver, 2007). Finally, we had fewer original datasets available when examining inter-group bias toward Asian individuals (than toward White or Black individuals). Overall, we conclude that the pooled results of the currently published original studies did not find evidence for a neural inter-group bias toward Asian individuals.

### 4.4. Methodological considerations

Due to a lack of a sufficient number of original studies, this meta-analysis could not investigate neural inter-group biases between different ethnic minority groups, or inter-group biases toward Arabs, Middle Eastern individuals, or Latinos. Some pieces of evidence suggest that ethnic discrimination is more evident toward Arabs and Middle Eastern than Asian individuals (Zschirnt and Ruedin, 2016). Further, some single findings have supported the presence of inter-group biases between ethnic minorities, such as between Arab and Israeli groups in the US (Bruneau et al., 2012). Thus, inter-group bias toward these ethnic groups remains an intriguing topic for future studies.

It is necessary to keep in mind that, the brain responses toward particular ethnicities (Black, White, Asian) may differ between individuals from different ethnic groups. In our analyses, it was not possible to examine brain responses toward White/Black/Asian individuals separately among White/Black/Asian individuals (due to limited number of studies in each category). Therefore, results per ethnicity might theoretically reflect the combination of activation clusters linked to the different ethnic out-groups. This remains a research question for future studies.

In all included studies, the fMRI tasks consisted of visual (including facial) processing of ethnic groups. Nevertheless, there was some degree of variation in the task content between single original studies: for example, whether the stimulus material included video clips or photographs; whether the stimulus material presented only faces (with neutral or painful expression) or also a body; and whether subjects were instructed to press a button on the basis of group membership or some other indicator (e.g., target individual’s gender). As a result, each sub-group meta-analysis included a slightly different combination of different fMRI tasks. The fMRI task of each original study is described in Supplementary Tables 3, 4. In order to increase comparability between the original studies, we included only studies with visual processing (excluded studies with merely auditory processing) and studies where the target individuals’ skin color could be seen (excluded studies where target individuals’ ethnic group was described only in a written statement).

Inter-group bias can encompass either in-group favoritism or out-group derogation. In this meta-analysis, however, it was not possible to certainly define whether the biased BOLD responses reflected either in-group favoritism or out-group derogation. This is because, first, most brain regions (e.g., the amygdala, frontal regions) are involved in both positive and negative emotions and may also process complex sociocognitive information. Second, when finding a significant difference (such as higher activity toward out- than in-group), it is not possible to deduce whether it refers to elevated activity to out-group or reduced activity to in-group. This is because the brain responses cannot be directly interpreted as reflecting changes in activity level (e.g., increased activity toward in-group vs. decreased activity toward out-group) but merely a difference in activity level.

Finally, this meta-analysis was limited to non-clinical study samples with adult-aged subjects. The original datasets were approximately balanced by sex: ca. 48.0–57.2% were female in different analyses of this manuscript. A large proportion of the original samples consisted of young adults, with the mean age of 24.1–32.8 years in our meta-analyses. Consequently, our results cannot be directly generalized to low-educated groups, clinical populations, elderly, adolescents, or children. Inter-group bias may be less evident in children (Abrams et al., 2003), and more evident in low-educated groups as low educational level associates with a stronger need for ethnic distance (Hello et al., 2006).

## 5. Conclusion

Ethnic inter-group bias seems not to constitute a universal pattern of neural responses toward visually presented ethnic in-group and out-group. On the contrary, neural responses to ethnic groups depend on (i) one’s own ethnic minority or majority status and (ii) the target individuals’ ethnic background. Neural inter-group bias appears to be non-evident among ethnic minority members (e.g., no signs of majority-group derogation) but clearly visible among majority members in many brain regions (involved in, e.g., facial processing, attention, and perspective-taking). Moreover, this meta-analysis found evidence for distinct response patterns toward different ethnic groups (White, Black, or Asian) that may possibly reflect certain sociocultural norms and attitudes. Taken together, this meta-analysis provides novel understanding of the neural mechanisms beyond ethnic inter-group bias and ethnic prejudices.

## Data availability statement

Publicly available datasets were analyzed in this study. This data can be found here: The full statistical data (e.g., all the coordinates and statistical estimates) can be requested from the corresponding author. Additionally, we have provided descriptive information of the included studies in Supplementary Table 3; the statistical contrasts selected from each study in Supplementary Table 4; and the primary reasons for excluding articles in Supplementary Table 5 (exclusion on the basis of title and abstract) and in Supplementary Table 6 (exclusion on the basis of full-text version).

## Author contributions

AS conducted the data analysis and wrote the initial manuscript draft. All authors contributed to the study design, interpretation of the data, and writing of the manuscript.
