# Supplementary material for: Ethnicity, minority status, and inter-group bias: A systematic meta-analysis on fMRI studies
Source: Front Hum Neurosci. 2023 Jan 6;16:1072345. doi: 10.3389/fnhum.2022.1072345 (PMC9852837; doi:10.3389/fnhum.2022.1072345)
Supplement: Supplementary file 1 [file Data_Sheet_1.DOCX]

**Supplementary Material**

**Supplementary Methods.** Search terms.

**PsycINFO:**

((fmri or functional MRI or BOLD) and (prejudice or ingroup or outgroup or group membership or group bias or ingroup favoritism or racial bias or ethnic bias)). ti.

((fmri or functional MRI or BOLD) and (prejudice or ingroup or outgroup or group membership or group bias or ingroup favoritism or racial bias or ethnic bias)). ab.

**PubMed:**

("fmri"[Title/Abstract] OR "functional MRI"[Title/Abstract] OR "BOLD"[Title/Abstract]) AND ("prejudice"[Title/Abstract] OR "ingroup"[Title/Abstract] OR "outgroup"[Title/Abstract] OR "group membership"[Title/Abstract] OR "group bias"[Title/Abstract] OR "ingroup favoritism"[Title/Abstract] OR "racial bias"[Title/Abstract] OR "ethnic bias"[Title/Abstract])

**Web of Science:**

TI=(("fmri" OR "functional MRI" OR "BOLD") AND ("prejudice" OR "ingroup" OR "outgroup" OR "group membership" OR "group bias" OR "ingroup favoritism" OR "racial bias" OR "ethnic bias")) OR AB=(("fmri" OR "functional MRI" OR "BOLD") AND ("prejudice" OR "ingroup" OR "outgroup" OR "group membership" OR "group bias" OR "ingroup favoritism" OR "racial bias" OR "ethnic bias"))

**Supplementary Methods.** The exclusion criteria during article search process, and further information about collection of the statistical details.

The exclusion criteria for the identified studies were: not an original study (e.g., a review or commentary); sample size < 10; findings of a same fMRI task within a same dataset were reported in another included study; a clinical sample (e.g., subjects had been exposed to traumatic events or medications); not exposure to a clearly defined ethnic or national out-group and in-group (e.g., in-group *vs*. neutral group, or out-group *vs*. the self, or political/trivial in-group vs. out-group); the sample of subjects included many ethnic groups that were not separately analyzed; the stimulus material did not include visual processing of ethnic in- and out-group members (but e.g. auditory processing of different accents); group membership was only defined via sociodemographic factors (age or gender); whole-brain analyses were not reported (e.g., only ROI-based, small-volume corrected, or strongly masked analyses); only connectivity-based analyses were conducted; or some necessary statistical information was not available.

When deriving the statistical details from the original studies, the subsequent principles were followed. First, if a study described both uncorrected and corrected results of whole-brain or voxel-wise analyses, uncorrected results were selected because the estimations of the SDM software are more accurate if including statistical information about more peaks (Radua & Albajes-Eizagirre, 2019). Second, if a statistical threshold value was not reported (but all other needed information was available), we used the threshold value p < .001 in accordance with previous recommendations (Radua & Albajes-Eizagirre, 2019). Third, if T statistics had not been reported, Z statistics or p values were converted into T statistics using a web-based tool (https://www.sdmproject.com/utilities/?show=Statistics). Finally, in case a study did not report an exact T or Z or p value for the needed statistical contrast, the SDM software imputed an estimated T value on the basis of the statistical threshold used in the study and the direction of the contrast (e.g., in-group > out-group).

**Data extraction from the included studies.** The following information was collected from each original study if available: study country, publication year, sample size, age and gender distribution, ethnic groups under investigation, subjects’ ethnic in-group, assessment method of subjects’ ethnic in-group (e.g., a self-report questionnaire or examination of family history), type of fMRI task (e.g. processing of faces), analyzing software package for fMRI, smoothing kernel (mm), magnetic field strength (Tesla), threshold value for statistical significance (if a study had used cluster-based analyses, the cluster-forming threshold was selected), and use of correction for multiple comparisons (correction on the basis of voxel height or voxel extent). Furthermore, if applicable, we collected the x-, y- and z-coordinates of statistically significant contrasts and the direction of those contrasts (e.g., Chinese > African). The *T* values of the statistical contrasts were reversed into a same direction (i.e., negative *T* values referred to higher BOLD responses to ethnic out-group than in-group, and positive *T* values referred to higher BOLD responses to ethnic in-group than out-group). Additional details of deriving the statistical contrasts can be found in **Supplementary Methods**. Furthermore, **Supplementary Table 4** lists the statistical contrasts that were used in this meta-analysis.

**Supplementary Table 1.** Descriptive information of the studies including samples with (a) majority status and (b) minority status.

| **First Author** | | **Publication Year** | | **Sample size** | **Female (%)** | **Mean age** | **Description of fMRI task** | **Study country (region)** | **Subjects' ethnic in-group** | **Subjects’ ethnic out-group** |
| --- | --- | --- | --- | --- | --- | --- | --- | --- | --- | --- |
|  | |  | |  |  |  |  |  |  |  |
| **(A) Samples with ethnic majority status** | | | | | | | | | | |
| Richeson | | 2003 | | 15 | 53.3 | 20.5 | Subjects viewed photographs depicting in-group and out-group members faces | US (New Hampshire, New Jersey) | White | Black |
| Cunningham | | 2004 | | 13 | 30.8 | 27.0 | Subjects viewed photographs of in-group and out-group members’ faces | US (Yale) | White | Black |
| Lee | | 2008 | | 13 | 0.0 | 24.8 | Subjects viewed photographs of in-group and out-group members’ faces | South Korea (Uijeongbu) | Korean | Caucasian |
| van Bavel | | 2008 | | 17 | 58.8 | NA | Subjects viewed faces of in-group and out-group members and categorized them as belonging to in-group or out-group | US (Ohio) | White | Black |
| Xu | | 2009A | | 17 | 52.9 | 23.0 | Subjects viewed video clips where in-group and out-group members were touched on their face by a pain-inducing or non-pain-inducing object | China (Peking) | Chinese | Caucasian |
| Freeman | | 2010 | | 16 | 50.0 | NA | Subjects viewed faces of in-group and out-group members, read a series of judgments (superficial or individuated) about their personality, and rated whether the statements were true or not | US (Massachusetts) | White | Black |
| Mathur | | 2010B | | 14 | 92.9 | NA | Subjects viewed naturalistic visual scenes depicting in-group and out-group members in a painful or neutral situation | US (Northwest) | Caucasian-American | African-American |
| Rule | | 2010A | | 14 | NA | NA | Subjects viewed in-group and out-group faces and made voting decisions (i.e., either to vote or not) | US (Massachusetts) | American | Japanese |
| Feng | | 2011 | | 30 | 36.7 | 23 | Subjects viewed faces of in-group and out-group members and categorized them on the basis of race or similarity with previously presented face | China (Beijing) | Han Chinese | Caucasian |
| Gilbert | | 2012 | | 20 | 30.0 | 19 | Subjects viewed in-group and out-group members’ faces and judged them based on an evaluation (who would you befriend?) or a stereotype-relevant trait (who is more likely to enjoy athletic activities?) | US (New York) | White | Black |
| Contreras | | 2013 | | 17 | 52.9 | 22.2 | Subjects visually processed photographs of in-group vs. out-group members and categorized them on the basis of race or sex | US (Harvard, Cambridge) | White | Black |
| Contreras-Huerta | | 2013 | | 20 | 60.0 | 22.5 | Subjects viewed visual scenes when in-group and out-group members received either painful or non-painful touch | Australia (Queensland) | Caucasian-Australian | Chinese |
| Losin | | 2013 | | 20 | 50.0 | 22.9 | Subjects viewed portraits of in-group and out-group members, observed their gestures, and imitated their gestures | US (Los Angeles) | European-American | African American or Han Chinese American |
| Zuo | | 2013 | | 20 | 60.0 | 23.3 | Subjects viewed video clips where in-group and out-group members were touched on their face by a pain-inducing or non-pain-inducing object | China (Peking) | Chinese | Caucasian |
| Sheng | | 2014 | | 21 | 52.4 | 22.0 | Subjects viewed faces of in-group and out-group members with painful or neutral facial expressions | China (Peking) | Chinese | Caucasian |
| Li | | 2015A | | 16 | 25.0 | 22.4 | Subjects viewed faces of in-group or out-group members with painful or neutral facial expressions (subjects had been primed with either mortality salience or negative affect) | China (Peking) | Asian (Chinese) | Caucasian |
| Li | | 2015B1 | | 20 | 45.0 | 23.2 | Subjects viewed faces of in-group or out-group members with painful or neutral facial expressions (subjects had been primed with either mortality salience or negative affect) | China (Peking) | Asian (Chinese) | Caucasian |
| Li | | 2015B2 | | 20 | 60.0 | 22.0 | Subjects viewed faces of in-group or out-group members with painful or neutral facial expressions (subjects had been primed with either mortality salience or negative affect) | China (Peking) | Asian (Chinese) | Caucasian |
| Liu | | 2015 | | 26 | 50.0 | 21.2 | Subjects passively viewed in-group and out-group members’ faces with different facial emotions | China (Beijing) | Chinese | African-American |
| Luo | | 2015A | | 30 | 46.7 | 20.2 | Subjects viewed video clips where in-group and out-group members were touched on their face by a pain-inducing or non-pain-inducing object | China (Peking) | Chinese | Caucasian |
| Luo | | 2015B | | 30 | 46.7 | 20.3 | Subjects viewed video clips where in-group and out-group members were touched on their face by a pain-inducing or non-pain-inducing object | China (Peking) | Chinese | Caucasian |
| Molapour | | 2015 | | 20 | 50.0 | 22.4 | Subjects viewed pictures of in-group and out-group members, and either received or did not receive electroshocks | Sweden (Stockholm) | White | Black |
| Rauchbauer | | 2015 | | 41 | 43.9 | 22.6 | Subjects viewed facial expressions of in-group and out-group members and imitated them (in congruent and incongruent conditions) | Austria (Vienna) | White European-Caucasian | African-American |
| Telzer | | 2015A | | 13 | 53.8 | 19.0 | Subjects viewed faces of in-group and out-group members, and made decisions to sacrifice earning money in order to donate to an in-group or out-group member (or, alternatively, subjects could earn money themselves at no cost to the confederates) | US (Illinois) | American | Chinese |
| Wang | | 2015 | | 30 | 46.7 | 22.6 | Subjects viewed video clips where in-group and out-group members were touched on their face by a pain-inducing or non-pain-inducing object (after independent or interdependent priming) | China (Peking) | Asian (Chinese) | Caucasian |
| Berlingeri | | 2016 | | 25 | 52.0 | 25.3 | Subjects viewed pictures of in-group and out-group members who were being touched by a painful or non-painful object | Italy (Milano) | Caucasian | African |
| Firat | | 2017 | | 13 | 42.5 | 47.2 | Subjects viewed pictures of in-group and out-group members in socially high-valued or low-valued positions | US (Iowa) | Caucasian | Black |
| Fourie | | 2017B | | 19 | 57.9 | 40.1 | Subjects viewed short video clips of in-group and out-group members who expressed either painful or neutral expressions | South Africa (Cape Town) | African-American | White |
| Watson | | 2017 | | 21 | 61.9 | 22.0 | Subjects viewed photographs of in-group and out-group members with angry and happy body postures | Netherlands (Maastricht) | White | African |
| Dominguez | | 2018 | | 48 | 72.9 | 25.3 | Subjects viewed pictures of in-group and out-group members and decided to shoot or not shoot the person (depending on whether the target seemed to hold a gun or some other object) | Australia (Monash) | Caucasian | Non-Caucasian (Arabian Muslim) |
| Katsumi | | 2018 | | 20 | 50.0 | NA | Subjects observed social encounters with ingroup and outgroup members displaying dynamic and static non-verbal behaviors | US (Illinois) | Caucasian / White | Non-Caucasian (East Asian, South Asian, and African–American) |
| Lin | | 2018A | | 22 | NA | NA | Subjects watched photographs of in-group and out-group members, rated pleasantness of images, then viewed the same images along with information on how in-group and out-group members had rated them, and finally rated the images again themselves | US (Illinois) | American (White) | Asian |
| Mattan | | 2018 | | 60 | 0.0 | 23.8 | Subjects viewed faces of in-group and out-group members who were described to high or low socioeconomic position | US (Chicago) | White | Black |
| McCutcheon | | 2018A | | 19 | 52.6 | 24.0 | Subjects viewed photographs depicting in-group and out-group members’ faces | US (throughout the country) | White | Black |
| Krosch | | 2019 | | 30 | 53.3 | 19.6 | Subjects viewed faces of in-group and out-group members and made decisions of allocating them different amounts of funds (in economically scarce or neutral circumstances) | US (New York) | White | Black |
| Yan | | 2019B | | 24 | 50.0 | 25.4 | Subjects viewed faces of in-group and out-group members and categorized them as belonging to in-group or out-group | Germany (Mannheim) | German | Chinese |
| Cassidy | | 2020 | | 75 | 62.7 | 45.0 | Subjects viewed pictures of faces of in-group and out-group members (with neutral facial expressions) | US (Indiana) | White | Black or Asian |
| Farmer | | 2020 | | 25 | 68.0 | 25.2 | Subjects viewed faces of in-group and out-group members and categorized faces on the basis of e.g. their age | UK (Plymouth) | White | Black |
| Rubien-Thomas | | 2021A | | 51 | 52.9 | 26.0 | Subjects viewed photographs of in-group and out-group members’ faces displaying a neutral expression | US (New York, New Haven, Connecticut) | White | Black |
|  | |  | |  |  |  |  |  |  |  |
| **(B) Samples with ethnic minority status** | | | | | | | | | | |
| Xu | | 2009B | | 16 | 50.0 | 23.0 | Subjects viewed video clips where in-group and out-group members were touched on their face by a pain-inducing or non-pain-inducing object | China (Peking) | Caucasian | Chinese |
| Mathur | | 2010A | | 14 | 71.4 | NA | Subjects viewed naturalistic visual scenes depicting in-group and out-group members in a painful or neutral situation | US (Northwest) | African-American | Caucasian-American |
| Rule | | 2010B | | 14 | NA | NA | Subjects viewed in-group and out-group faces and made voting decisions (i.e., either to vote or not) | US (Massachusetts) | Japanese | American |
| Cao | | 2015 | | 30 | 60.0 | 23.2 | Subjects observed videos of in-group and out-group members receiving painful or non-painful touch | China (Mainland) | Chinese | Caucasian |
| Chen | | 2015 | | 26 | NA | NA | Subjects were presented photographs of in-group and out-group members with different facial expressions (without awareness) | US (Dartmouth) | Chinese | Caucasian |
| Telzer | | 2015B | | 13 | 46.2 | 19.4 | Subjects viewed faces of in-group and out-group members, and made decisions to sacrifice earning money in order to donate to an in-group or out-group member (or, alternatively, subjects could earn money themselves at no cost to the confederates) | US (Illinois) | Chinese | American |
| Fourie | | 2017A | | 19 | 52.6 | 41.5 | Subjects viewed short video clips of in-group and out-group members who expressed either painful or neutral expressions | South Africa (Cape Town) | Caucasian-White | Black / African-American |
| Lin | | 2018B | | 23 | NA | NA | Subjects watched photographs of in-group and out-group members, rated pleasantness of images, then viewed the same images along with information on how in-group and out-group members had rated them, and finally rated the images again themselves | US (Illinois) | Chinese | White |
| McCutcheon | | 2018B | | 17 | 58.8 | 24.5 | Subjects viewed photographs depicting in-group and out-group members’ faces | UK (throughout the country) | Black | White |
| Yan | | 2019A | | 20 | 45.0 | 26.0 | Subjects viewed faces of in-group and out-group members and categorized them as belonging to in-group or out-group | Germany (Mannheim) | Chinese | German |
| Rubien-Thomas | | 2021B | | 54 | 53.7 | 25.8 | Subjects viewed photographs of in-group and out-group members’ faces displaying a neutral expression | US | Black | White |
|  |  | |  |  |  |  |  |  |  |  |

**Supplementary Table 2.** Descriptive information of the studies with (a) White target persons, (b) Black target persons, and (c) Asian target persons.

| **First Author** | | **Publication Year** | | | | **Sample size** | | | **Female (%)** | | | **Mean age** | | | **Description of fMRI task** | | | **Subjects' ethnic in-group** | | | **Study country (region)** | |  |  |  |
| --- | --- | --- | --- | --- | --- | --- | --- | --- | --- | --- | --- | --- | --- | --- | --- | --- | --- | --- | --- | --- | --- | --- | --- | --- | --- |
|  | | |  |  | | | | | | | | | | | | | | | | | | |  |  |  |
| **(A) White target persons** | | | | | | | | | | | | | | | | | | | | | | |  |  |  |
| Lee | | 2008 | | | | 13 | | | 0.0 | | | 24.8 | | | Subjects viewed photographs of in-group and out-group members’ faces | | | Korean | | | South Korea (Uijeongbu) | |  |  |  |
| Xu | | 2009A | | | | 17 | | | 52.9 | | | 23.0 | | | Subjects viewed video clips where in-group and out-group members were touched on their face by a pain-inducing or non-pain-inducing object | | | Chinese | | | China (Peking) | |  |  |  |
| Mathur | | 2010A | | | | 14 | | | 71.4 | | | NA | | | Subjects viewed naturalistic visual scenes depicting in-group and out-group members in a painful or neutral situation | | | African-American | | | US (Northwest) | |  |  |  |
| Rule | | 2010B | | | | 14 | | | NA | | | NA | | | Subjects viewed in-group and out-group faces and made voting decisions (i.e., either to vote or not) | | | Japanese | | | US (Massachusetts) | |  |  |  |
| Feng | | 2011 | | | | 30 | | | 36.7 | | | 23.0 | | | Subjects viewed faces of in-group and out-group members and categorized them on the basis of race or similarity with previously presented face | | | Han Chinese | | | China (Beijing) | |  |  |  |
| Zuo | | 2013 | | | | 20 | | | 60.0 | | | 23.3 | | | Subjects viewed video clips where in-group and out-group members were touched on their face by a pain-inducing or non-pain-inducing object | | | Chinese | | | China (Peking) | |  |  |  |
| Sheng | | 2014 | | | | 21 | | | 52.4 | | | 22.0 | | | Subjects viewed faces of in-group and out-group members with painful or neutral facial expressions | | | Chinese | | | China (Peking) | |  |  |  |
| Cao | | 2015 | | | | 30 | | | 60.0 | | | 23.2 | | | Subjects observed videos of in-group and out-group members receiving painful or non-painful touch | | | Chinese | | | Australia (Queensland) | |  |  |  |
| Chen | | 2015 | | | | 26 | | | NA | | | NA | | | Subjects were presented photographs of in-group and out-group members with different facial expressions (without awareness) | | | Chinese | | | US (Dartmouth) | |  |  |  |
| Li | | 2015A | | | | 16 | | | 25.0 | | | 22.4 | | | Subjects viewed faces of in-group or out-group members with painful or neutral facial expressions (subjects had been primed with either mortality salience or negative affect) | | | Asian (Chinese) | | | China (Peking) | |  |  |  |
| Li | | 2015B^1^ | | | | 20 | | | 45.0 | | | 23.2 | | | Subjects viewed faces of in-group or out-group members with painful or neutral facial expressions (subjects had been primed with either mortality salience or negative affect) | | | Asian (Chinese) | | | China (Peking) | |  |  |  |
| Li | | 2015B^1^ | | | | 20 | | | 60.0 | | | 22.0 | | | Subjects viewed faces of in-group or out-group members with painful or neutral facial expressions (subjects had been primed with either mortality salience or negative affect) | | | Asian (Chinese) | | | China (Peking) | |  |  |  |
| Luo | | 2015A | | | | 30 | | | 46.7 | | | 20.2 | | | Subjects viewed video clips where in-group and out-group members were touched on their face by a pain-inducing or non-pain-inducing object | | | Chinese | | | China (Peking) | |  |  |  |
| Luo | | 2015B | | | | 30 | | | 46.7 | | | 20.3 | | | Subjects viewed video clips where in-group and out-group members were touched on their face by a pain-inducing or non-pain-inducing object | | | Chinese | | | China (Peking) | |  |  |  |
| Telzer | | 2015B | | | | 13 | | | 46.2 | | | 19.4 | | | Subjects viewed faces of in-group and out-group members, and made decisions to sacrifice earning money in order to donate to an in-group or out-group member (or, alternatively, subjects could earn money themselves at no cost to the confederates) | | | Chinese | | | US (Illinois) | |  |  |  |
| Wang | | 2015 | | | | 30 | | | 46.7 | | | 22.6 | | | Subjects viewed video clips where in-group and out-group members were touched on their face by a pain-inducing or non-pain-inducing object (after independent or interdependent priming) | | | Asian (Chinese) | | | China (Peking) | |  |  |  |
| Fourie | | 2017B | | | | 19 | | | 57.9 | | | 40.1 | | | Subjects viewed short video clips of in-group and out-group members who expressed either painful or neutral expressions | | | Black / African-American | | | South Africa (Cape Town) | |  |  |  |
| Lin | | 2018B | | | | 23 | | | NA | | | NA | | | Subjects watched photographs of in-group and out-group members, rated pleasantness of images, then viewed the same images along with information on how in-group and out-group members had rated them, and finally rated the images again themselves | | | Chinese | | | US (Illinois) | |  |  |  |
| McCutcheon | | 2018B | | | | 17 | | | 58.8 | | | 24.5 | | | Subjects viewed photographs depicting in-group and out-group members’ faces | | | Black | | | UK (throughout the country) | |  |  |  |
| Yan | | 2019A | | | | 20 | | | 45.0 | | | 26.0 | | | Subjects viewed faces of in-group and out-group members and categorized them as belonging to in-group or out-group | | | Chinese | | | Germany (Mannheim) | |  |  |  |
| Rubien-Thomas | | 2021B | | | | 54 | | | 53.7 | | | 25.8 | | | Subjects viewed photographs of in-group and out-group members’ faces displaying a neutral expression | | | Black | | | US (New York, New Haven, Connecticut) | |  |  |  |
|  |  | | | |  | | |  | | |  | | |  | |  | | |  | | |  | | |  |
| **(B) Black target persons** | | | | | | | | | | | | | | | | | | | | | | |  |  |  |
| Richeson | 2003 | | | | 15 | | | 53.3 | | | 20.5 | | | Subjects viewed photographs depicting in-group and out-group members faces | | | | White | | | US (New Hampshire, New Jersey) | | |  |  |
| Cunningham | 2004 | | | | 13 | | | 30.8 | | | 27.0 | | | Subjects viewed photographs of in-group and out-group members’ faces | | | | White | | | US (Yale) | | |  |  |
| van Bavel | 2008 | | | | 17 | | | 58.8 | | | NA | | | Subjects viewed faces of in-group and out-group members and categorized them as belonging to in-group or out-group | | | | White | | | US (Ohio) | | |  |  |
| Freeman | 2010 | | | | 16 | | | 50.0 | | | NA | | | Subjects viewed faces of in-group and out-group members, read a series of judgments (superficial or individuated) about their personality, and rated whether the statements were true or not | | | | White | | | US (Massachusetts) | | |  |  |
| Mathur | 2010B | | | | 14 | | | 92.9 | | | NA | | | Subjects viewed naturalistic visual scenes depicting in-group and out-group members in a painful or neutral situation | | | | Caucasian American | | | US (Northwest) | | |  |  |
| Gilbert | 2012 | | | | 20 | | | 30.0 | | | 19.0 | | | Subjects viewed in-group and out-group members’ faces and judged them based on an evaluation (who would you befriend?) or a stereotype-relevant trait (who is more likely to enjoy athletic activities?) | | | | White | | | US (New York) | | |  |  |
| Contreras | 2013 | | | | 17 | | | 52.9 | | | 22.2 | | | Subjects visually processed photographs of in-group vs. out-group members and categorized them on the basis of race or sex | | | | White | | | US (Harvard, Cambridge) | | |  |  |
| Losin | 2013 | | | | 20 | | | 50.0 | | | 22.9 | | | Subjects viewed portraits of in-group and out-group members, observed their gestures, and imitated their gestures | | | | European American | | | US (Los Angeles) | | |  |  |
| Liu | 2015 | | | | 26 | | | 50.0 | | | 21.2 | | | Subjects passively viewed in-group and out-group members’ faces with different facial emotions | | | | Chinese | | | China (Beijing) | | |  |  |
| Molapour | 2015 | | | | 20 | | | 50.0 | | | 22.4 | | | Subjects viewed pictures of in-group and out-group members, and either received or did not receive electroshocks | | | | White | | | Sweden (Stockholm) | | |  |  |
| Rauchbauer | 2015 | | | | 41 | | | 43.9 | | | 22.6 | | | Subjects viewed facial expressions of in-group and out-group members and imitated them (in congruent and incongruent conditions) | | | | White European-Caucasian | | | Austria (Vienna) | | |  |  |
| Berlingeri | 2016 | | | | 25 | | | 52.0 | | | 25.3 | | | Subjects viewed pictures of in-group and out-group members who were being touched by a painful or non-painful object | | | | Caucasian | | | Italy (Milano) | | |  |  |
| Firat | 2017 | | | | 13 | | | 42.5 | | | 47.2 | | | Subjects viewed pictures of in-group and out-group members in socially high-valued or low-valued positions | | | | Caucasian | | | US (Iowa) | | |  |  |
| Fourie | 2017A | | | | 19 | | | 52.6 | | | 41.5 | | | Subjects viewed short video clips of in-group and out-group members who expressed either painful or neutral expressions | | | | Caucasian-White | | | South Africa (Cape Town) | | |  |  |
| Watson | 2017 | | | | 21 | | | 61.9 | | | 22.0 | | | Subjects viewed photographs of in-group and out-group members with angry and happy body postures | | | | White European | | | Netherlands (Maastricht) | | |  |  |
| Mattan | 2018 | | | | 60 | | | 0.0 | | | 23.8 | | | Subjects viewed faces of in-group and out-group members who were described to high or low socioeconomic position | | | | White | | | US (Chicago) | | |  |  |
| McCutcheon | 2018A | | | | 19 | | | 52.6 | | | 24.0 | | | Subjects viewed photographs depicting in-group and out-group members’ faces | | | | White | | | UK (recruited throughout the country) | | |  |  |
| Krosch | 2019 | | | | 30 | | | 53.3 | | | 19.6 | | | Subjects viewed faces of in-group and out-group members and made decisions of allocating them different amounts of funds (in economically scarce or neutral circumstances) | | | | White | | | US (New York) | | |  |  |
| Cassidy | 2020 | | | | 75 | | | 62.7 | | | 45.0 | | | Subjects viewed pictures of faces of in-group and out-group members (with neutral facial expressions) | | | | White | | | US (Indiana) | | |  |  |
| Farmer | 2020 | | | | 25 | | | 68.0 | | | 25.2 | | | Subjects viewed faces of in-group and out-group members and categorized faces on the basis of e.g. their age | | | | White | | | UK (Plymouth) | | |  |  |
| Rubien-Thomas | 2021A | | | | 51 | | | 52.9 | | | 26.0 | | | Subjects viewed photographs of in-group and out-group members’ faces displaying a neutral expression | | | | White | | | US | | |  |  |
|  |  | | | |  | |  | | | |  | | |  | |  | | |  | | |  | | |  |
| **(C) Asian target persons** | | | | | | | | | | | | | | | | | | | | | | |  |  |  |
| Xu | 2009B | | | | 16 | | 50.0 | | | 23.0 | | | Subjects viewed video clips where in-group and out-group members were touched on their face by a pain-inducing or non-pain-inducing object | | | | Caucasian | | | China (Peking) | | |  |  |  |
| Rule | 2010A | | | | 14 | | NA | | | NA | | | Subjects viewed in-group and out-group faces and made voting decisions (i.e., either to vote or not) | | | | American | | | US (Massachusetts) | | |  |  |  |
| Contreras-Huerta | 2013 | | | | 20 | | 60.0 | | | 22.5 | | | Subjects viewed visual scenes when in-group and out-group members received either painful or non-painful touch | | | | White (Caucasian-Australian) | | | Australia (Queensland) | | |  |  |  |
| Losin | 2013 | | | | 20 | | 50.0 | | | 22.9 | | | Subjects viewed portraits of in-group and out-group members, observed their gestures, and imitated their gestures | | | | European American | | | US (Los Angeles) | | |  |  |  |
| Telzer | 2015A | | | | 13 | | 53.8 | | | 19.0 | | | Subjects viewed faces of in-group and out-group members, and made decisions to sacrifice earning money in order to donate to an in-group or out-group member (or, alternatively, subjects could earn money themselves at no cost to the confederates) | | | | American | | | US (Illinois) | | |  |  |  |
| Lin | 2018A | | | | 22 | | NA | | | NA | | | Subjects watched photographs of in-group and out-group members, rated pleasantness of images, then viewed the same images along with information on how in-group and out-group members had rated them, and finally rated the images again themselves | | | | American | | | US (Illinois) | | |  |  |  |
| Yan | 2019B | | | | 24 | | 50 | | | 25.4 | | | Subjects viewed faces of in-group and out-group members and categorized them as belonging to in-group or out-group | | | | German | | | Germany (Mannheim) | | |  |  |  |
| Cassidy | 2020 | | | | 75 | | 62.7 | | | 45.0 | | |  | | | | White | | | US (Indiana) | | |  |  |  |
|  | | |  |  | | | | | | | | | | | | | | | | | | |  |  |  |

**Supplementary Table 3.** Additional descriptive information of the included studies.

| **First author** | **Publication year** | **Subjects’ ethnic group** | **Additional information on subjects’ ethnic background** | **Study country (region)** |
| --- | --- | --- | --- | --- |
| Berlingeri, M. | 2016 | Caucasian | NA | Italy (Milano) |
| Cao, Y. | 2015 | Chinese | All subjects had lived in Australia for < 5 years, including eleven new arrivals (residential time < 6 months). All subjects were born in Mainland China, had two Chinese parents, and finished primary and secondary schooling in China. | Australia (Queensland) |
| Cassidy, B. S. | 2020 | White | All subjects self-identified themselves as White. | US (Indiana) |
| Chen, P. A. | 2015 | Chinese | Newly arrived Chinese international graduate students who were recruited within the first month of their arrival in the US. None of them had ever stayed or studied in a foreign country for more than 2 months prior to their arrival in the US. All the subjects were fluent in both Chinese and English. | US (Dartmouth) |
| Contreras, J. M. | 2013 | White | Subjects were college students and community members from Cambridge and described themselves as White. | US (Cambridge) |
| Contreras-Huerta, L. S. | 2013 | Caucasian-Australian | Caucasian-Australian subjects were born in Australia, had white skin, and had Caucasian, Anglo-Saxon parents. | Australian (Queensland) |
| Cunningham, W. A. | 2004 | White | NA | US (Yale) |
| Dominguez, D. J. F. | 2018 | Caucasian | NA | Australia (Monash) |
| Farmer, H. | 2020 | White | Subjects self-identified themselves as White. | UK (Plymouth) |
| Feng, L. | 2011 | Han Chinese | Subjects were living in Beijing, PR China where 99.99% of the population is Han Chinese. All subjects were prescreened to ensure that they had no prior direct contact with any Caucasian individuals. | China (Beijing) |
| Firat, R. B. | 2017 | Caucasian | NA | US (Iowa) |
| Fourie, M. M.^1^ | 2017A,B | Caucasian-White or African-American | Ethnic group was defined on the basis of the South African Population Registration Act of 1950, which divided the population into four racial groups: Whites, Blacks (Natives), Indians and Coloureds (people of mixed racial ancestry). All subjects lived in South Africa during apartheid (before 1994). | South Africa (Cape Town) |
| Freeman, J. B. | 2010 | White | NA | US (Massachusetts) |
| Gilbert, S. J. | 2012 | White | Subjects were White and native-English speakers who were recruited through flyers placed around the New York University campus. | US (New York) |
| Katsumi, Y. | 2018 | Caucasian (White) | All subjects were native English speakers and identified their race as Caucasian/White. | US (Illinois) |
| Krosch, A. R. | 2019 | White | Subjects were native English-speaking undergraduate students. | US (New York) |
| Lee, K. U. | 2008 | Korean | NA | South Korea (Uijeongbu) |
| Li, X. ^1^ | 2015A,B | Chinese | Chinese college students | China (Peking) |
| Lieberman, M. D. ^1^ | 2005A,B | Caucasian-American or African-American | NA | US (Los Angeles) |
| Lin, L. C. ^1^ | 2018A,B | American or Chinese | American subjects were White/Caucasian. Chinese subjects were born in China and had been in the United States for less than one year. | US (Illinois) |
| Liu, Y. | 2015 | Chinese | All subjects were Chinese students and reported no or minimal contact with Black people: none of the subjects had been abroad or had any intergroup contact with African people according to their self-reports. Six subjects had seen Black people in real life. | China (Beijing) |
| Losin, E. A. R. | 2013 | European-American | NA | US (Los Angeles) |
| Luo, S. Y. ^1^ | 2015A,B | Chinese | Chinese university students | China (Peking) |
| Mathur, V. A. ^1^ | 2010A,B | African-American or Caucasian-American | NA | US (Northwest) |
| Mattan, B. D. | 2018 | White | Subjects self-identified themselves as White, had lived in the US for at least 5 years, and had a good command of the English language. | US (Chicago) |
| McCutcheon, R. ^1^ | 2018A,B | White or Black | The Black subjects were first- or second-generation migrants, self-identified as being Black, and were classified as of either Black Caribbean or Black African ethnicity on the basis of either their own (first-generation migrants) or their parents’ (second-generation migrants) country of origin. The White subjects self-identified as being of White British ethnicity and had at least one parent of UK nationality. | UK (recruited throughout the UK) |
| Molapour, T. | 2015 | White | The subjects were of European decent. | Sweden (Stockholm) |
| Rauchbauer, B. | 2015 | European-Caucasian | NA | Austria (Vienna) |
| Richeson, J. A. | 2003 | White (American) | NA | US (New Hampshire, New Jersey) |
| Rubien-Thomas, E. ^1^ | 2021A,B | White or Black | NA | US |
| Rule, N. O. ^1^ | 2010A,B | American or Japanese | The American subjects were university students in the US. The Japanese subjects were students at a Japanese university who were in the US participating in a study-abroad program. None of the subjects had spent more than a month in the US, including the time between their arrival and the date of scanning. | US (Massachusetts) |
| Sheng, F. | 2014 | Chinese | NA | China (Peking) |
| Telzer, E. H. ^1^ | 2015A,B | American or Chinese | The American subjects were born in the US. The Chinese subjects were born in China and had moved to the US less than one year prior to their scan. | US (Illinois) |
| Van Bavel, J. J. | 2008 | White | NA | US (Ohio) |
| Wang, C. | 2015 | Chinese | NA | China (Peking) |
| Watson, R. | 2017 | White (European) | The subjects represented a homogeneous ethnic group recruited from the local student population. None of them had any personal experience with other ethnical groups. | The Netherlands (Maastricht) |
| Xu, X. ^1^ | 2009A,B | Chinese or Caucasian (10 Americans, 2 Dutch, 1 Italian, 1 German, 1 Russian, 1 Israeli) | NA | China (Peking) |
| Yan, Z. ^1^ | 2019A,B | Chinese or German | NA | Germany (Mannheim) |
| Zuo, S. | 2013 | Chinese | Some subjects were born in China and emigrated to the US or Canada at an early age. Other subjects were born and brought up in the US or UK. | China (Peking) |
| ^1^ Note: This study included two datasets. | | | | |

**Supplementary Table 4.** Additional information on analytical methods.

| **First author** | **Publication year** | **Analyzing software package for fMRI** | **Smoothing kernel (mm)** | **Magnetic field strength (Tesla)** | **Statistical comparisons of fMRI results that were used in the meta-analysis**  *Note*: * indicates that some additional information was received from the authors of the original publication |
| --- | --- | --- | --- | --- | --- |
| Richeson, J. A. | 2003 | SPM99 | 6 | 1.5 | Experiment 1: Main effect of group membership |
| Cunningham, W. A. | 2004 | SPM99 | 9 | 1.5 | Presentation of faces for 30 ms: response to Black vs. White faces  Presentation of faces for 525 ms: response to Black vs. White faces |
| Lee, K. U. | 2008 | SPM2 | NA | 1.5 | Neutral face: other race > own race  Emotional (happy/sad) face: own race > other race |
| Van Bavel, J. J. | 2008 | SPM5 | 9 | 3 | Main effect of race processing |
| Xu, X. | 2009A | SPM2 | 8 | 3 | Among Chinese subjects: needle penetration vs. Q-tip applied to racial in-group and out-group faces |
| Xu, X. | 2009B | SPM2 | 8 | 3 | Among Caucasian subjects: needle penetration vs. Q-tip applied to racial in-group and out-group faces |
| Freeman, J. B. | 2010 | BrainVoyager QX | 7 | 3 | Judgment × race effect (the effect of individuated vs. superficial judgments for White vs. Black targets) |
| Mathur, V. A. | 2010A | SPM2 | 8 | 3 | African-American group: pain of in-group vs. out-group |
| Mathur, V. A. | 2010B | SPM2 | 8 | 3 | Caucasian-American group: pain of in-group vs. out-group |
| Rule, N. O. | 2010A | BrainVoyager QX | 7 | 3 | American subjects: processing in-group vs. out-group (not considering voting behavior) |
| Rule, N. O. | 2010B | BrainVoyager QX | 7 | 3 | Japanese subjects: processing in-group vs. out-group (not considering voting behavior) |
| Feng, L. | 2011 | SPM8 | 8 | 3 | Categorization task: faces of in-group vs. out-group members  Localizer task: faces of in-group vs. out-group members |
| Gilbert, S. J. | 2012 | SPM8 | 4 | 3 | Judgement of White vs. Black faces  Friendship (evaluative) judgments of White vs. Black faces  Stereotype-related trait judgement of White vs. Black faces |
| Contreras-Huerta, L. S. | 2013 | SPM8 | 6 | 3 | Racial groups: Observing painful vs. non-painful touch in own-group members compared with other-group members, and vice-versa |
| Contreras, J. M. | 2013 | SPM8 | 8 | 3 | Categorization of faces of in-group vs. out-group members |
| Losin, E. A. R. | 2013 | FSL, AFNI, ART | 6 | 3 | Imitate gesture of European American vs. African American  Imitate gesture of European American vs. Han Chinese  Viewing of European American vs. Han Chinese  Viewing of European American vs. African American  Gesture observation of European American vs. Han Chinese |
| Zuo, S. | 2013 | SPM8 | 8 | 3 | Pain of in-group vs. out-group members |
| Sheng, F. | 2014 | SPM8 | 8 | 3 | Pain judgements: Asian face (Pain − Neutral) vs. Caucasian face (Pain − Neutral)  Racial judgements: Asian face (Pain − Neutral) vs. Caucasian face (Pain − Neutral) |
| Cao, Y. | 2015 | SPM8 | 6 | 3 | Pain x group membership (own race vs. other race) |
| Chen, P. A. | 2015 | SPM8 | 6 | 3 | Main effect of group membership  Interaction between facial expression and group membership |
| Li, X. | 2015A | SPM8 | 8 | 3 | Experiment 1: Mortality salience group: 436 ms after stimulus onset: pain toward in-group vs. out-group  Experiment 1: Mortality salience group: 456 ms after stimulus onset: pain of in-group vs. out-group |
| Li, X. | 2015B | SPM8 | 8 | 3 | Experiment 2: Mortality salience priming group: pain of in-group vs. out-group |
| Li, X. | 2015B | SPM8 | 8 | 3 | Experiment 2: Negative affect priming group: pain of in-group vs. out-group |
| Liu, Y. | 2015 | SPM8 | 6 | 3 | Main effect of viewing in-group vs. out-group faces  Viewing disgusted faces of in-group vs. out-group members |
| Luo, S. Y. | 2015A | SPM8 | 8 | 3 | Among A/A individuals: (painful–non-painful)_Asian faces_ minus (painful–non-painful)_Caucasian faces_ |
| Luo, S. Y. | 2015B | SPM8 | 8 | 3 | Among G/G individuals: (painful–non-painful)_Asian faces_ minus (painful–non-painful)_Caucasian faces_ |
| Molapour, T. | 2015 | SPM8 | 8 | 3 | Acquisition: Overall activity (White vs. Black)  Acquisition: Overall activity (White shock vs. Black shock)  Acquisition: linear change over time: White vs. Black  Acquisition: linear change over time: Black shock vs. White shock  Extinction: linear changer over time: White vs. Black  Extinction: linear changer over time: Black shock vs. White shock  Extinction: Overall activity (White vs. Black)  Extinction: Overall activity (Black shock vs. White shock) |
| Rauchbauer, B. | 2015 | SPM8 | 8 | 3 | Out-group (Incongruent > Congruent) masked inclusively with Out-group (Incongruent > Congruent) > In-group (Incongruent > Congruent)  In-group (Incongruent > Congruent) masked inclusively with In-group (Incongruent > Congruent) > Out-group (Incongruent > Congruent) |
| Telzer, E. H. | 2015A | SPM8 | 8 | 3 | Among American subjects: prosocial donations to in-group vs. out-group members |
| Telzer, E. H. | 2015B | SPM8 | 8 | 3 | Among Chinese subjects: prosocial donations to in-group vs. out-group members |
| Wang, C. | 2015 | SPM8 | 8 | 3 | Independent self-construal priming condition: pain of in-group vs. out-group member  Interdependent self-construal priming condition: pain of in-group vs. out-group members |
| Berlingeri, M. | 2016 | SPM8 | 8 | 1.5 | Stimulus phase: pain responses toward in-group vs. out-group *  Response phase: pain responses toward in-group vs. out-group * |
| Firat, R. B. | 2017 | AFNI | 4 | 3 | Experiment 2: visual viewing of pictures: high White vs. high Black  Experiment 2: visual viewing of pictures: low White vs. low Black  Experiment 2: visual viewing of pictures: middle White vs. middle Black |
| Fourie, M. M. | 2017A | BrainVoyager QX | NA | 3 | Among White subjects: physical pain of in-group vs. out-group *  Among White subjects: social pain of in-group vs. out-group * |
| Fourie, M. M. | 2017B | BrainVoyager QX | NA | 3 | Among Black subjects: physical pain of in-group vs. out-group *  Among Black subjects: social pain of in-group vs. out-group * |
| Watson, R. | 2017 | BrainVoyager | 6 | 3 | Over both tasks: Black bodies vs. White bodies *  Over both tasks: Black angry bodies vs. White angry bodies *  Over both tasks: Black happy bodies vs. White happy bodies *  Emotion categorisation task: Black bodies vs. White bodies *  Emotion categorisation task: Black angry bodies vs. White angry bodies *  Emotion categorisation task: Black happy bodies vs. White happy bodies *  Shape categorisation task: Black bodies vs. White bodies *  Shape categorisation task: Black angry bodies vs. White angry bodies *  Shape categorisation task: Black happy bodies vs. White happy bodies * |
| Dominguez, D. J. F. | 2018 | SPM12 | 9 | 3 | Unjustified shooting decisions: correctly shooting armed Muslim targets vs. non-Muslim targets  Justified shooting decisions: incorrectly shooting armed Muslim targets vs. non-Muslim targets |
| Katsumi, Y. | 2018 | SPM8 | 8 | 3 | Main effect of race when observing static or approach or avoidance behavior of in-group vs. out-group member  Main effect of race when observing handshake (or not) from in-group vs. out-group member |
| Lin, L. C. | 2018A | FSL, SPM8 | 6 | 3 | Tracking of social influence from in-group vs. out-group members |
| Lin, L. C. | 2018B | FSL, SPM8 | 6 | 3 | Tracking of social influence from in-group vs. out-group members |
| Mattan, B. D. | 2018 | SPM8 | 8 | 3 | Main effect of race  Low-status Black – high-status Black > low-status White – high-status White |
| McCutcheon, R. | 2018A | SPM8 | 8 | 3 | Among White ethnicity group: White faces vs. Black faces |
| McCutcheon, R. | 2018B | SPM8 | 8 | 3 | Among Black ethnicity group: White faces vs. Black faces |
| Krosch, A. R. | 2019 | SPM8 | 6 | 3 | Main effect of viewing Black vs. White faces |
| Yan, Z. | 2019A | SPM12 | 9 | 3 | Among Chinese subjects: in-ethnicity versus out-ethnicity faces * |
| Yan, Z. | 2019B | SPM12 | 9 | 3 | Among German subjects: in-ethnicity versus out-ethnicity faces * |
| Cassidy, B. S. | 2020 | SPM12 | 8 | 3 | Main effect of race * |
| Farmer, H. | 2020 | SPM12 | 8 | 1.5 | Main effect of race on facial processing of White vs. Black faces |
| Rubien-Thomas, E. | 2021A | FSL | 5 | 3 | Among White subjects: Black faces vs. White faces |
| Rubien-Thomas, E. | 2021B | FSL | 5 | 3 | Among Black subjects: Black faces vs. White faces |

**Supplementary Table 5.** Article selection process on the basis of title and abstract screening, and primary reasons of excluding articles.

| **First Author** | **Publication Year** | **Journal or source** | **Inclusion / exclusion on the basis of title and abstract** | **Primary reason for exclusion** |
| --- | --- | --- | --- | --- |
| Allen, E. | 1836 | New York, NY, US: G W & A J Matsell; US | Out | Book |
| Blake, R. R. | 1961 | New York, NY, US: Harper and Brothers; US | Out | Book |
| Kroll, W. | 1965 | Research Quarterly | Out | The study did not include brain imaging |
| Uleman, J. S. | 1991 | Contemporary Psychology | Out | Essay |
| Knight, A. | 1994 | Copeia | Out | The study examined snakes, not humans |
| Hart, A. J. | 2000 | Neuroreport | In |  |
| Cho, C. J. | 2002 | Land Use Pol. | Out | A paper on Korean society, not related to inter-group bias or prejudices |
| Grossman, H. | 2002 | Springfield, IL, US: Charles C Thomas Publisher; US | Out | Book |
| Richeson, J. A. | 2003 | Nat. Neurosci. | In |  |
| Gehring, W. J. | 2003 | Nature Neuroscience | Out | Commentary paper |
| Madsen, A. K. | 2003 | NA | Out | Conference abstract |
| Bloom, S. G. | 2005 | Smithsonian | Out | Not an original paper related to inter-group bias or prejudices |
| Olsson, A. | 2006 | Dissertation Abstracts International: Section B: The Sciences and Engineering | Out | Dissertation abstract |
| Knutson, K. M. | 2007 | Hum. Brain Mapp. | In |  |
| Shami, M. J. | 2007 | Analyses of Social Issues and Public Policy | Out | Commentary paper |
| Beer, J. S. | 2008 | Neuroimage | In |  |
| Rilling, J. K. | 2008 | Neuroimage. | In |  |
| Wright, C. I. | 2008 | Neuroimage | Out | Group membership consisted of age groups |
| Sui, J. | 2008 | 42nd Asilomar Conference on Signals, Systems and Computers | Out | The study did not examine inter-group bias or prejudices |
| Sugiman, T. | 2008 | New York, NY, US: Springer Science + Business Media; US | Out | Book |
| Krendl, A. C. | 2009 | Psychol. Aging | In |  |
| Krill, A. | 2009 | Front Evol Neurosci. | In |  |
| Derntl, B. | 2009 | BMC Neurosci. | In |  |
| Nosarti, C. | 2009 | Neuroimage | Out | The study examined prenatally born children |
| Sui, J. | 2009 | Hum. Brain Mapp. | Out | The study did not examine inter-group bias or prejudices |
| Sui, J. | 2009 | Neuroimage | Out | The study examined schizophrenia patients |
| Szycik, G. R. | 2009 | Schizophr. Res. | Out | The study examined schizophrenia patients |
| James, G. A. | 2009 | Neuroimage | Out | The study did not examine inter-group bias or prejudices |
| Amodio, D. M. | 2009 | New York, NY, US: Psychology Press; US | Out | Book |
| Cikara, M. | 2010 | Soc. Cogn. Affect. Neurosci. | In |  |
| Bruneau, E. G. | 2010 | Neuroimage | In |  |
| Rule, N. O. | 2010 | Soc. Cogn. Affect. Neurosci. | In |  |
| Adams, R. B. | 2010 | J. Cogn. Neurosci. | In |  |
| Cunningham, W. A. | 2010 | Soc. Cogn. | Out | Commentary paper |
| Brown, T. | 2010 | Stanford Law Rev. | Out | Review paper |
| Van Bavel, J. J. | 2011 | J. Cogn. Neurosci. | In |  |
| Masten, C. L. | 2011 | J. Cogn. Neurosci. | In |  |
| Hellstrom, G. | 2011 | Behav. Ecol. Sociobiol. | Out | The study examined risk-taking of Eurasian perch |
| Kobayashi, M. | 2011 | PLoS One | Out | The study did not examine inter-group bias or prejudices |
| Sobhani, M. | 2012 | PLoS One | In |  |
| Morrison, S. | 2012 | Neuropsychologia | In |  |
| Baumgartner, T. | 2012 | Hum. Brain Mapp. | In |  |
| Stanley, D. A. | 2012 | Philos. Trans. R. Soc. B-Biol. Sci. | In |  |
| Korn, H. A. | 2012 | Soc. Neurosci. | In |  |
| Seebohm, P. | 2012 | Community Dev. J. | Out | The study did not examine inter-group bias or prejudices |
| Ruckmann, J. | 2012 | Int. J. Behav. Med. | Out | Meeting abstract |
| Jenoff, P. | 2012 | Univ. Cincinnati Law Rev. | Out | Review |
| Mourao-Miranda, J. | 2012 | PLoS One | Out | The study examined psychiatric disorders |
| Gallate, J. | 2012 | Hauppauge, NY, US: Nova Science Publishers; US | Out | Book |
| He, Y. | 2012 | issertation Abstracts International: Section B: The Sciences and Engineering | Out | Dissertation abstract |
| Contreras-Huerta, L. S. | 2013 | PLoS One | In |  |
| Azevedo, R. T. | 2013 | Hum. Brain Mapp. | In |  |
| Ratner, K. G. | 2013 | Soc. Cogn. Affect. Neurosci. | In |  |
| Scheepers, D. | 2013 | Front. Hum. Neurosci. | In |  |
| Fox, G. R. | 2013 | Front Psychol. | In |  |
| Molenberghs, P. | 2013 | Hum Brain Mapp. | In |  |
| Stallen, M. | 2013 | Front Hum Neurosci. | In |  |
| Ebner, N. C. | 2013 | Neuroimage | Out | Group membership consisted of age groups |
| Shkurko, A. V. | 2013 | Soc. Cogn. Affect. Neurosci. | Out | Meta-analysis |
| Deshpande, G. | 2013 | Front. Hum. Neurosci. | Out | The study examined brain networks related to autism |
| Bookheimer, S. Y. | 2013 | Evid.-based Complement Altern. Med. | Out | The study examined memory problems and pomegranate juice |
| Telzer, E. H. | 2013 | J Neurosci. | Out | The study examined adolescents with early adversities |
| Das, P. | 2013 | Biol Psychiatry. | Out | The study examined young girls’ emotional problems |
| Rotella, K. N. | 2013 | New York, NY, US: Psychology Press; US | Out | Book |
| Molenberghs, P. | 2014 | Hum. Brain Mapp. | In |  |
| Fourie, M. M. | 2014 | Soc. Neurosci. | In |  |
| Sheng, F. | 2014 | Neuroimage. | In |  |
| Molenberghs, P. | 2014 | Soc Cogn Affect Neurosci. | In |  |
| Heitzeg, M. M. | 2014 | Drug Alcohol Depend. | Out | The study examined adolescents’ substance use disorders |
| Mavromihelaki, E. | 2014 |  | Out | Proceedings paper |
| Wei, W. J. | 2014 |  | Out | Proceedings paper |
| Rüsch, N. | 2014 | Soc Neurosci. | Out | The study examined family coherence, not inter-group bias or prejudices |
| Pariyadath, V. | 2014 | Front Hum Neurosci. | Out | The study examined smoking, not inter-group bias or prejudices |
| Firat, R. B. | 2014 | Dissertation Abstracts International Section A: Humanities and Social Sciences | Out | Dissertation abstract |
| Hannum, K. M. | 2014 | Harvard Business School Publishing, Boston, US | Out | Book |
| Ruckmann, J. | 2015 | Psychiatry Res. Neuroimaging | In |  |
| Terbeck, S. | 2015 | Psychopharmacology (Berl). | In |  |
| Cao, Y. | 2015 | Cortex | In |  |
| Rauchbauer, B. | 2015 | Cortex | In |  |
| Telzer, E. H. | 2015 | Neuroimage | In |  |
| Luo, S. Y. | 2015 | Neuroimage | In |  |
| Kim, K. | 2015 | Soc. Cogn. Affect. Neurosci. | In |  |
| Li, X. | 2015 | Neuroimage. | In |  |
| Bestelmeyer, P. E. | 2015 | Cereb Cortex. | In |  |
| Wang, C. | 2015 | Soc Cogn Affect Neurosci. | In |  |
| Senholzi, K. B. | 2015 | Soc Neurosci. | In |  |
| Littlefield, M. M. | 2015 | Front Hum Neurosci. | In |  |
| Chen, P. A. | 2015 | Soc Psychol Personal Sci. | In |  |
| Forbes, C. E. | 2015 | Group Process Intergroup Relat. | Out | Review paper |
| Melchers, M. | 2015 | Neuroimage. | Out | The study did not examine inter-group bias or prejudices |
| Iannaccone, R. | 2015 | Eur Child Adolesc Psychiatry. | Out | The study examined adolescents’ attention-deficit hyperactivity disorder |
| Chester, D. S. | 2015 | Soc Cogn Affect Neurosci. | Out | The study examined alexithymia |
| Blackford, J. U. | 2015 | Schizophr Res. | Out | Group membership was defined on the basis of gender |
| Marsh, L. E. | 2016 | Neuroimage | In |  |
| Berlingeri, M. | 2016 | Neuroscience | In |  |
| Powers, K. E. | 2016 | J. Cogn. Neurosci. | In |  |
| Krendl, A. C. | 2016 | PLoS One | In |  |
| Molenberghs, P. | 2016 | Cereb. Cortex | In |  |
| Dunsmoor, J. E. | 2016 | Soc Cogn Affect Neurosci. | In |  |
| Hein, G. | 2016 | Proc Natl Acad Sci U S A. | In |  |
| Scheepers, D. | 2016 | Curr. Opin. Psychol. | Out | Review article |
| Barrera, M. L. | 2016 | Calif. Hist. | Out | Essay paper |
| Cetin, M. S. | 2016 | Front. Neurosci. | Out | The study examined schizophrenia patients |
| Bolling, D. Z. | 2016 | Soc. Neurosci. | Out | The sample consisted of children and adolescents |
| Cohn, M. D. | 2016 | Soc. Cogn. Affect. Neurosci. | Out | The study examined disruptive behavior |
| Krueger, F. | 2016 | Int. J. Psychol. | Out | Conference abstract |
| Chen, Q. F. | 2016 | PLoS One | Out | The study did not examine inter-group bias or prejudices |
| Etzel, J. A. | 2016 | PLoS One | Out | The study did not examine inter-group bias or prejudices |
| van IJzendoorn M. H. | 2016 | J Neuroendocrinol. | Out | Review paper |
| Culbreth, A. J. | 2016 | Schizophr Bull. | Out | The study examined schizophrenia patients |
| Senholzi, K. B. | 2016 | San Diego, CA, US: Elsevier Academic Press; US | Out | Book |
| Kubota, J. | 2016 | New York, NY, US: Psychology Press; US | Out | Book |
| Page-Gould, E. | 2016 | New York, NY, US: Routledge/Taylor & Francis Group; U | Out | Book |
| Ragland, J. D. | 2016 | Biological Psychiatry | Out | Commentary paper |
| Krosch, A. R. | 2016 | Dissertation Abstracts International: Section B: The Sciences and Engineering | Out | Dissertation abstract |
| Moradi, Z. | 2017 | Behav Brain Res. | In |  |
| Haas, I. J. | 2017 | Soc. Justice Res. | In |  |
| Greven, I. M. | 2017 | Neuropsychologia | In |  |
| Gamond, L. | 2017 | Eur. J. Neurosci. | In |  |
| Molenberghs, P. | 2017 | J. Manag. | In |  |
| Lau, T. | 2017 | Sci Rep | In |  |
| Feng, C. L. | 2017 | Hum. Brain Mapp. | In |  |
| Spiers, H. J. | 2017 | J. Cogn. Neurosci. | In |  |
| Fourie M. M. | 2017 | Soc Cogn Affect Neurosci. | In |  |
| Brown T. I. | 2017 | PLoS One. | In |  |
| Reimers, L. | 2017 | Neuroimage. | In |  |
| Hackel, L. M. | 2017 | Soc Cogn Affect Neurosci. | In |  |
| Firat, R. B. | 2017 | Soc Cogn Affect Neurosci. | In |  |
| Gopal, K. V. | 2017 | J. Am. Acad. Audiol. | Out | The study did not examine inter-group bias but tinnitus |
| Guo, H. N. | 2017 | Chin. J. Appl. Linguist. | Out | Review paper |
| Chen, X. | 2017 | Psychoneuroendocrinology. 2017 Apr | Out | The study did not examine inter-group bias or prejudices |
| Just, M. A. | 2017 | Nat Hum Behav. | Out | The study examined suicidality |
| Ryan, J. J. | 2017 | PsycCRITIQUES | Out | The study did not examine inter-group bias or prejudices |
| Sahakian, B. | 2017 | New York, NY, US: Oxford University Press | Out | Book |
| Jiang, X. M. | 2018 | Neuroimage | In |  |
| Lin, L. C. | 2018 | Proc. Natl. Acad. Sci. U. S. A. | In |  |
| McCutcheon, R. | 2018 | Psychol. Med. | In |  |
| Apps, M. A. J. | 2018 | Brain Behav. | In |  |
| Mattan, B. D. | 2018 | eNeuro | In |  |
| Katsumi, Y. | 2018 | Front. Hum. Neurosci. | In |  |
| Mattan, B. D. | 2018 | Soc. Cogn. Affect. Neurosci. | In |  |
| Dominguez, D. J. F. | 2018 | Soc. Neurosci. | In |  |
| Wu, C. T. | 2018 | Front Hum Neurosci. | In |  |
| Carpenter, A. C. | 2018 | Soc Neurosci. | In |  |
| Molenberghs, P. | 2018 | Front. Psychol. | Out | Review article |
| Titcombe-Parekh, R. F. | 2018 | J. Psychiatr. Res. | Out | The study examined postraumatic stress disorder |
| Appiah, O | 2018 | J. Commun. | Out | A commentary-style paper |
| Nah, Y. | 2018 | Neurobiol. Learn. Mem. | Out | The study examined post-partum females (not inter-group bias or prejudices) |
| Golestani, A. M. | 2018 | Brain Connect. | Out | The study did not examine inter-group bias or prejudices |
| Han, S. | 2018 | Trends Cogn Sci. | Out | Review article |
| Pretus, C. | 2018 | Front Psychol. | Out | The study examined brain functioning of jihadists, not inter-group bias or prejudices |
| Oddo-Sommerfeld, S. | 2018 | Neuropsychologia. | Out | The study examined e.g. amputation, not inter-group bias or prejudices |
| Duarte, I. C. | 2018 | Sci Rep. | Out | The study did not examine inter-group bias or prejudices |
| Gonzalez, F. J. | 2018 | Dissertation Abstracts International Section A: Humanities and Social Sciences | Out | Dissertation abstract |
| Richins, M. T. | 2019 | Soc. Neurosci. | In |  |
| Hughes, C. | 2019 | Front. Hum. Neurosci. | In |  |
| Andrews, T. J. | 2019 | Cereb Cortex. | In |  |
| Yan, Z. | 2019 | Soc Cogn Affect Neurosci. | In |  |
| Liuzza, M. T. | 2019 | Sci Rep. | In |  |
| Izuma, K. | 2019 | Neuroimage. | In |  |
| Hughes, B. L. | 2019 | Proc Natl Acad Sci U S A. | In |  |
| Endendijk, J. J. | 2019 | Biol. Psychol. | Out | The study did not examine inter-group bias or prejudices |
| Pillay, S. R. | 2019 | Am. Psychol. | Out | A narrative historical paper |
| Minkova, L. | 2019 | Front. Neurosci. | Out | The study examined amnesia, not inter-group bias or prejudices |
| Yang, X. | 2019 | BMC Psychiatry | Out | The study examined obsessive-compulsive disorder |
| Atikah, A. R. N. | 2019 | J. Asia-Pac. Entomol. | Out | The study examined plants, not humans |
| Qu, Y. | 2019 | Front. Hum. Neurosci. | Out | The study did not examine inter-group bias or prejudices |
| Weaver, D. | 2019 | Zoosymposia | Out | The study examined plants, not humans |
| Pillay, S. R. | 2019 | Am Psychol. | Out | Not an original paper, did not use fMRI, did not examine inter-group bias or prejudices |
| Do, K. T. | 2019 | Dev Cogn Neurosci. | Out | The sample consisted of children and adolescents |
| Savjani, R. | 2019 | Dissertation Abstracts International: Section B: The Sciences and Engineering | Out | Dissertation abstract |
| Cassidy, B. S. | 2020 | Aging Neuropsychol. Cogn. | In |  |
| Shin, W. G. | 2020 | Front. Behav. Neurosci. | In |  |
| Kesner, L. | 2020 | Front. Behav. Neurosci. | In |  |
| van Gils, S. | 2020 | J. Neurosci. Psychol. Econ. | In |  |
| Lantos, D. | 2020 | Soc. Neurosci. | In |  |
| Steines, M. | 2020 | Cortex | In |  |
| Nugiel, T. | 2020 | J. Cogn. Neurosci. | In |  |
| Li, Z. A. | 2020 | Neuroimage | In |  |
| Han, X. C. | 2020 | eLife | In |  |
| Farmer, H. | 2020 | Sci Rep | In |  |
| Lelieveld, G. J. | 2020 | Soc Cogn Affect Neurosci. | In |  |
| Harada, T. | 2020 | Neuropsychologia. | In |  |
| Vijayakumar, S. | 2020 | Soc Cogn Affect Neurosci. | In |  |
| Reggev, N. | 2020 | eNeuro. | In |  |
| Park, B. | 2020 | J Exp Soc Psychol. | In |  |
| Stange, J. P. | 2020 | Psychol. Med. | Out | The study did not examine inter-group bias or prejudices |
| Huang, J. P. | 2020 | Neurosci. Lett. | Out | The study did not examine inter-group bias or prejudices |
| Fombad, C. M. | 2020 | Hague J. Rule Law | Out | The paper was not an original study |
| Bahder, B. W. | 2020 | Zootaxa | Out | The study did not examine inter-group bias or prejudices |
| Boyd, B. | 2020 | Style | Out | The study did not examine inter-group bias or prejudices |
| Bagnis, A. | 2020 | Neuroimage. | Out | Meta-analysis |
| Assari, S. | 2020 | Res Health Sci. | Out | The sample consisted of children; the study examined hippocampal volume |
| Morese, R. | 2020 | Front Behav Neurosci. | Out | Theoretical review on Parkinson’s disease |
| Telzer, E. H. | 2020 | Dev Psychopathol. | Out | The sample consisted of adolescents |
| Bahder, B. W. | 2020 | Zootaxa. | Out | The study examined plants, not humans |
| Parvathaneni, P. | 2020 | Dissertation Abstracts International: Section B: The Sciences and Engineering | Out | Dissertation abstract |
| Rubien-Thomas, E. | 2021 | Cogn Affect Behav Neurosci. | In |  |
| Jie, J. | 2021 | Soc Cogn Affect Neurosci. | In |  |
| Liu, Y. | 2021 | Cereb Cortex. | In |  |
| Cassidy, B. S. | 2021 | Aging Neuropsychol. Cogn. | In |  |
| Kang, P. | 2021 | J Neurosci. | In |  |
| Merritt, C. C. | 2021 | Soc Cogn Affect Neurosci. | Out | Meta-analysis |
| Uzendu, A. I. | 2021 | Catheter. Cardiovasc. Interv. | Out | Review / essay |
| Brandner, B. | 2021 | Dev Cogn Neurosci. | Out | The study did not include any clear in-group vs. out-group design and examined adolescents |
| Lantos, D. | 2021 | Neurosci Biobehav Rev. | Out | Review |
| Tsuruha, E. | 2021 | Front Behav Neurosci. | Out | The study defined group membership on the basis of age |
| Jones, J. S. | 2021 | Dev Cogn Neurosci. | Out | The study examined behavioral problems of neuro-atypical children |
| Saarinen, A. | 2021 | Neurosci Biobehav Rev. | Out | Meta-analysis |
| Amodio, D. M. | 2021 | Annu Rev Psychol. | Out | Review article |
| Evangelista. N. D. | 2021 | Cereb Cortex. | Out | The study did not examine inter-group bias or prejudices |

**Supplementary Table 6.** Primary reasons for exclusion of original articles on the basis of reading full-text versions.

| **First author** | **Publication year** | **Title of the original publication** | **Primary reason for exclusion** |
| --- | --- | --- | --- |
| Hart, A. J. | 2000 | Differential response in the human amygdala to racial outgroup vs ingroup face stimuli | Too small sample size (*n* < 10) |
| Knutson, K. M. | 2007 | Neural correlates of automatic beliefs about gender and race | In-group vs. out-group contrast (in BOLD responses) not reported |
| Beer, J. S. | 2008 | The Quadruple Process model approach to examining the neural underpinnings of prejudice | In-group vs. out-group contrast (in BOLD responses) not reported |
| Rilling, J. K. | 2008 | Social cognitive neural networks during in-group and out-group interactions | In-group vs. out-group contrast (in BOLD responses) not reported |
| Derntl, B. | 2009 | General and specific responsiveness of the amygdala during explicit emotion recognition in females and males | The study did not report whole-brain analyses (used ROI-based analyses and strong masking) |
| Krendl, A. C. | 2009 | Aging Minds and Twisting Attitudes: An fMRI Investigation of Age Differences in Inhibiting Prejudice | The group membership was defined on the basis of stigmatization (not ethnic background) |
| Krill, A. | 2009 | In-group and out-group membership mediates anterior cingulate activation to social exclusion | The study reported only ROI-based analyses |
| Adams, R. B. | 2010 | Cross-cultural Reading the Mind in the Eyes: An fMRI Investigation | The study reported only ROI-based analyses |
| Bruneau, E. G. | 2010 | Attitudes towards the outgroup are predicted by activity in the precuneus in Arabs and Israelis | The study did not examine responses to clear and visible ethnic in-group and out-group |
| Cikara, M. | 2010 | On the wrong side of the trolley track: neural correlates of relative social valuation | Subjects not exposed to clearly defined in-group and out-group |
| Masten, C. L. | 2011 | An fMRI Investigation of Attributing Negative Social Treatment to Racial Discrimination | Subjects not exposed to clearly defined in-group and out-group |
| Van Bavel, J. J. | 2011 | Modulation of the Fusiform Face Area following Minimal Exposure to Motivationally Relevant Faces: Evidence of In-group Enhancement (Not Out-group Disregard) | The study reported only ROI-based analyses |
| Baumgartner, T. | 2012 | The mentalizing network orchestrates the impact of parochial altruism on social norm enforcement | The group membership was defined on the basis of army platoon (not ethnic background) |
| Korn, H. A. | 2012 | Neurolaw: Differential brain activity for Black and White faces predicts damage awards in hypothetical employment discrimination cases | In-group vs. out-group contrast (in BOLD responses) not reported |
| Morrison, S. | 2012 | The neuroscience of group membership | The group membership was not based on ethnic background |
| Sobhani, M. | 2012 | Interpersonal Liking Modulates Motor-Related Neural Regions | The direction of in-group vs. out-group findings not reported |
| Stanley, D. A. | 2012 | Race and reputation: perceived racial group trustworthiness influences the neural correlates of trust decisions | Subjects not exposed to clearly defined in-group and out-group |
| Azevedo, R. T. | 2013 | Their Pain is Not Our Pain: Brain and Autonomic Correlates of Empathic Resonance With the Pain of Same and Different Race Individuals | The study did not report findings separately for each ethnic group |
| Fox, G. R. | 2013 | Witnessing hateful people in pain modulates brain activity in regions associated with physical pain and reward | The group membership was defined on the basis of political / religious orientation (not ethnic background) |
| Molenberghs, P. | 2013 | Seeing is believing: neural mechanisms of action-perception are biased by team membership | The group membership was based on trivial criteria (not ethnic groups) |
| Ratner, K. G. | 2013 | Is race erased? Decoding race from patterns of neural activity when skin color is not diagnostic of group boundaries | The study reported only ROI-based analyses |
| Scheepers, D. | 2013 | The neural correlates of in-group and self-face perception: is there overlap for high identifiers? | The study compared responses to students of different universities (not ethnic groups) |
| Stallen, M. | 2013 | Peer influence: neural mechanisms underlying in-group conformity | The study did not report whole-brain analyses (used strong masking) |
| Fourie, M. M. | 2014 | Neural correlates of experienced moral emotion: An fMRI investigation of emotion in response to prejudice feedback | Subjects not exposed to clearly defined in-group and out-group |
| Molenberghs, P. | 2014 | The role of the medial prefrontal cortex in social categorization | The group membership was based on trivial criteria (not ethnic groups) |
| Molenberghs, P. | 2014 | The Influence of Group Membership and Individual Differences in Psychopathy and Perspective Taking on Neural Responses When Punishing and Rewarding Others | The group membership was based on trivial criteria (not ethnic groups) |
| Bestelmeyer, P. E. | 2015 | A Neural Marker for Social Bias Toward In-group Accents | The study did not examine visual processing of ethnic groups (but auditory processing of different accents) |
| Kim, K. | 2015 | Activity in ventromedial prefrontal cortex during self-related processing: positive subjective value or personal significance? | The group membership was defined on the basis of political orientation (not ethnic background) |
| Littlefield, M. M. | 2015 | Being asked to tell an unpleasant truth about another person activates anterior insula and medial prefrontal cortex | The group membership was based on trivial criteria (not ethnic groups) |
| Ruckmann, J. | 2015 | How pain empathy depends on ingroup/outgroup decisions: A functional magnet resonance imaging study | The group membership was based on trivial criteria (not ethnic groups) |
| Senholzi, K. B. | 2015 | Brain activation underlying threat detection to targets of different races | Subjects not exposed to clearly defined in-group and out-group |
| Terbeck, S. | 2015 | β-Adrenoceptor blockade modulates fusiform gyrus activity to black versus white faces | In-group vs. out-group contrast (in BOLD responses) not reported |
| Dunsmoor, J. E. | 2016 | Racial stereotypes impair flexibility of emotional learning | Subjects not exposed to clearly defined in-group and out-group |
| Hein, G. | 2016 | How learning shapes the empathic brain | The study compared responses to different European groups (not necessarily clear or visible ethnic differences) |
| Krendl, A. C. | 2016 | Does Older Adults' Cognitive Function Disrupt the Malleability of Their Attitudes toward Outgroup Members?: An fMRI Investigation | Subjects not exposed to clearly defined in-group and out-group |
| Marsh, L. E. | 2016 | The imitation game: Effects of social cues on 'imitation' are domain-general in nature | The group membership was based on trivial criteria (not ethnic groups) |
| Molenberghs, P. | 2016 | Increased Moral Sensitivity for Outgroup Perpetrators Harming Ingroup Members | The group membership was defined on the basis of university (not ethnic groups) |
| Powers, K. E. | 2016 | Striatal Associative Learning Signals Are Tuned to In-groups | In-group vs. out-group contrast not reported in whole-brain analyses |
| Brown, T. I. | 2017 | Cognitive control, attention, and the other race effect in memory | The study did not report findings separately for each ethnic group |
| Feng, C. L. | 2017 | Mortality Salience Reduces the Discrimination Between In-Group and Out-Group Interactions: A Functional MRI Investigation Using Multi-Voxel Pattern Analysis | The group membership was defined on the basis of both ethnic background and arbitrary group simultaneously |
| Gamond, L. | 2017 | Minimal group membership biases early neural processing of emotional expressions | In-group vs. out-group contrast (in BOLD responses) not reported |
| Greven, I. M. | 2017 | Neural network integration during the perception of in-group and out-group members | The study did not report whole-brain analyses (used strong masking) |
| Haas, I. J. | 2017 | Who Can Deviate from the Party Line? Political Ideology Moderates Evaluation of Incongruent Policy Positions in Insula and Anterior Cingulate Cortex | The study reported only ROI-based analyses |
| Hackel, L. M. | 2017 | Social identity shapes social valuation: evidence from prosocial behavior and vicarious reward | The study reported only ROI-based analyses |
| Lau, T. | 2017 | fMRI Repetition Suppression During Generalized Social Categorization | The group membership was defined on the basis of political orientation and trivial criteria (not ethnic groups) |
| Molenberghs, P. | 2017 | The Neuroscience of Inspirational Leadership: The Importance of Collective-Oriented Language and Shared Group Membership | The study examined different political groups (not ethnic groups) |
| Moradi, Z. | 2017 | Changes in intrinsic functional connectivity and group relevant salience: The case of sport rivalry | The group membership was based on favorite football team (not ethnicity) |
| Reimers, L. | 2017 | Neural substrates of male parochial altruism are modulated by testosterone and behavioral strategy | In-group vs. out-group contrast (in BOLD responses) not reported |
| Spiers, H. J. | 2017 | Anterior Temporal Lobe Tracks the Formation of Prejudice | Subjects not exposed to clearly defined in-group and out-group |
| Apps, M. A. J. | 2018 | Not on my team: Medial prefrontal cortex responses to ingroup fusion and unfair monetary divisions | The study used small-volume correction |
| Carpenter, A. C. | 2018 | Are eyewitness accounts biased? Evaluating false memories for crimes involving in-group or out-group conflict | Subjects not exposed to clearly defined in-group and out-group |
| Jiang, X. M. | 2018 | Neural architecture underlying person perception from in-group and out-group voices | The group membership was defined on the basis of accent (not ethnic background) |
| Mattan, B. D. | 2018 | Motivation Modulates Brain Networks in Response to Faces Varying in Race and Status: A Multivariate Approach | Findings of the same fMRI task within the same dataset were provided in another original paper |
| Wu, C. T. | 2018 | How Do Acquired Political Identities Influence Our Neural Processing toward Others within the Context of a Trust Game? | The group membership was based on political orientation (not ethnic background) |
| Andrews, T. J. | 2019 | Neural Correlates of Group Bias During Natural Viewing | Subjects not exposed to clearly defined in-group and out-group |
| Hughes, B. L. | 2019 | Neural adaptation to faces reveals racial outgroup homogeneity effects in early perception | The study reported only ROI-based analyses |
| Hughes, C. | 2019 | Culture Impacts the Neural Response to Perceiving Outgroups Among Black and White Faces | Subjects not exposed to clearly defined in-group and out-group |
| Izuma, K. | 2019 | Neural signals in amygdala predict implicit prejudice toward an ethnic outgroup | The study compared responses to material (e.g. flags) related to different Asian groups (not clear or visible ethnic differences) |
| Liuzza, M. T. | 2019 | An fMRI study on the neural correlates of social conformity to a sexual minority | Subjects not exposed to clearly defined in-group and out-group |
| Richins, M. T. | 2019 | Empathic responses are reduced to competitive but not non-competitive outgroups | The study compared responses to students of different universities (not ethnic groups) |
| Han, X. C. | 2020 | A neurobiological association of revenge propensity during intergroup conflict | In-group vs. out-group contrast not reported in whole-brain analyses |
| Harada, T. | 2020 | Cultural influences on neural systems of intergroup emotion perception: An fMRI study | In-group vs. out-group contrast (in BOLD responses) not reported |
| Kesner, L. | 2020 | Fusiform Activity Distinguishes Between Subjects With Low and High Xenophobic Attitudes Toward Refugees | Subjects not exposed to clearly defined in-group and out-group |
| Lantos, D. | 2020 | The neural mechanisms of threat and reconciliation efforts between Muslims and non-Muslims | Subjects not exposed to clearly defined in-group and out-group |
| Lelieveld, G. J. | 2020 | Jumping on the 'bad'wagon? How group membership influences responses to the social exclusion of others | Subjects not exposed to clearly defined in-group and out-group |
| Li, X. | 2020 | Guilty by association: How group-based (collective) guilt arises in the brain | The group membership was based on trivial criteria (not ethnic groups) |
| Nugiel, T. | 2020 | How Does Motivation Modulate the Operation of the Mentalizing Network in Person Evaluation? | The study examined different political groups (not ethnic groups) |
| Park, B. | 2020 | An association between biased impression updating and relationship facilitation: A behavioral and fMRI investigation | Subjects not exposed to clearly defined in-group and out-group |
| Reggev, N. | 2020 | Human Face-Selective Cortex Does Not Distinguish between Members of a Racial Outgroup | The study did not include whole-brain analyses |
| Shin, W. G. | 2020 | The Neurobehavioral Mechanisms Underlying Attitudes Toward People With Mental or Physical Illness | Subjects not exposed to clearly defined in-group and out-group |
| Steines, M. | 2020 | Conflicting group memberships modulate neural activation in an emotional production-perception network | The study examined responses to different trivial groups and different European nationalities (not clear ethnic groups) |
| van Gils, S. | 2020 | Better Together? The Neural Response to Moral Dilemmas Is Moderated by the Presence of a Close Other | The study examined responses to different Asian groups (not clear and visible ethnic differences) |
| Vijayakumar, S. | 2020 | Neural mechanisms of predicting individual preferences based on group membership | Subjects not exposed to clearly defined in-group and out-group |
| Cassidy, B. S. | 2021 | Age differences in neural activity related to mentalizing during person perception | The study reported only ROI-based analyses |
| Jie, J. | 2021 | Establishing a Counter-Empathy Processing Model: Evidence from Functional Magnetic Resonance Imaging | The study did not include any clear in-group and out-group |
| Kang, P. | 2021 | Why We Learn Less from Observing Outgroups | The group membership was defined on the basis of political orientation (not ethnic background) |
| Liu, Y. | 2021 | Oxytocin Modulates Neural Individuation/Categorization Processing of Faces in Early Face-Selective Areas | The study did not report whole-brain analyses |

**Supplementary Figure 1.** Funnel plots of the meta-analysis including all the eligible studies.

**MNI coordinates: x = -22, y = -78, z = 46**

**
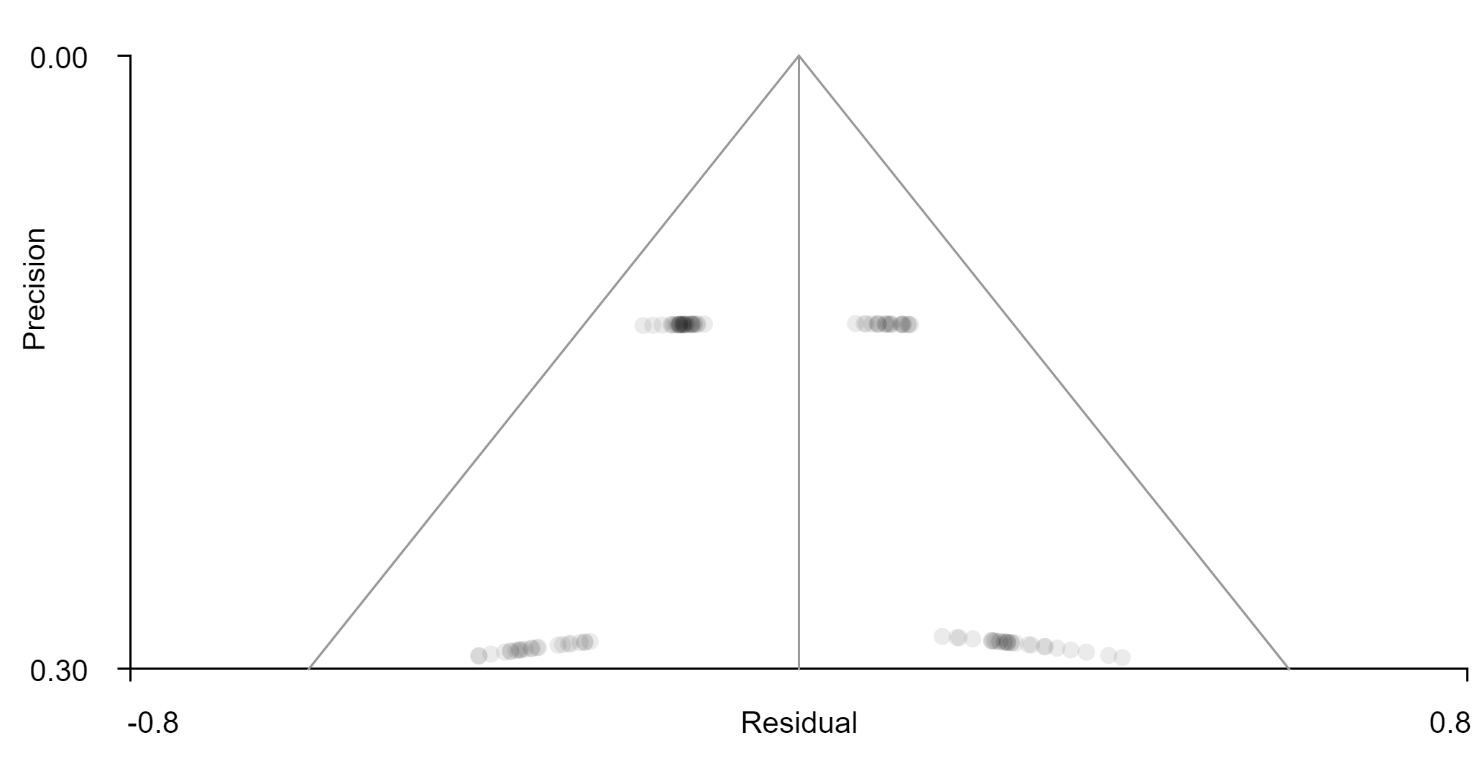
**

**MNI coordinates: x = -6, y = -48, z = 28**

**
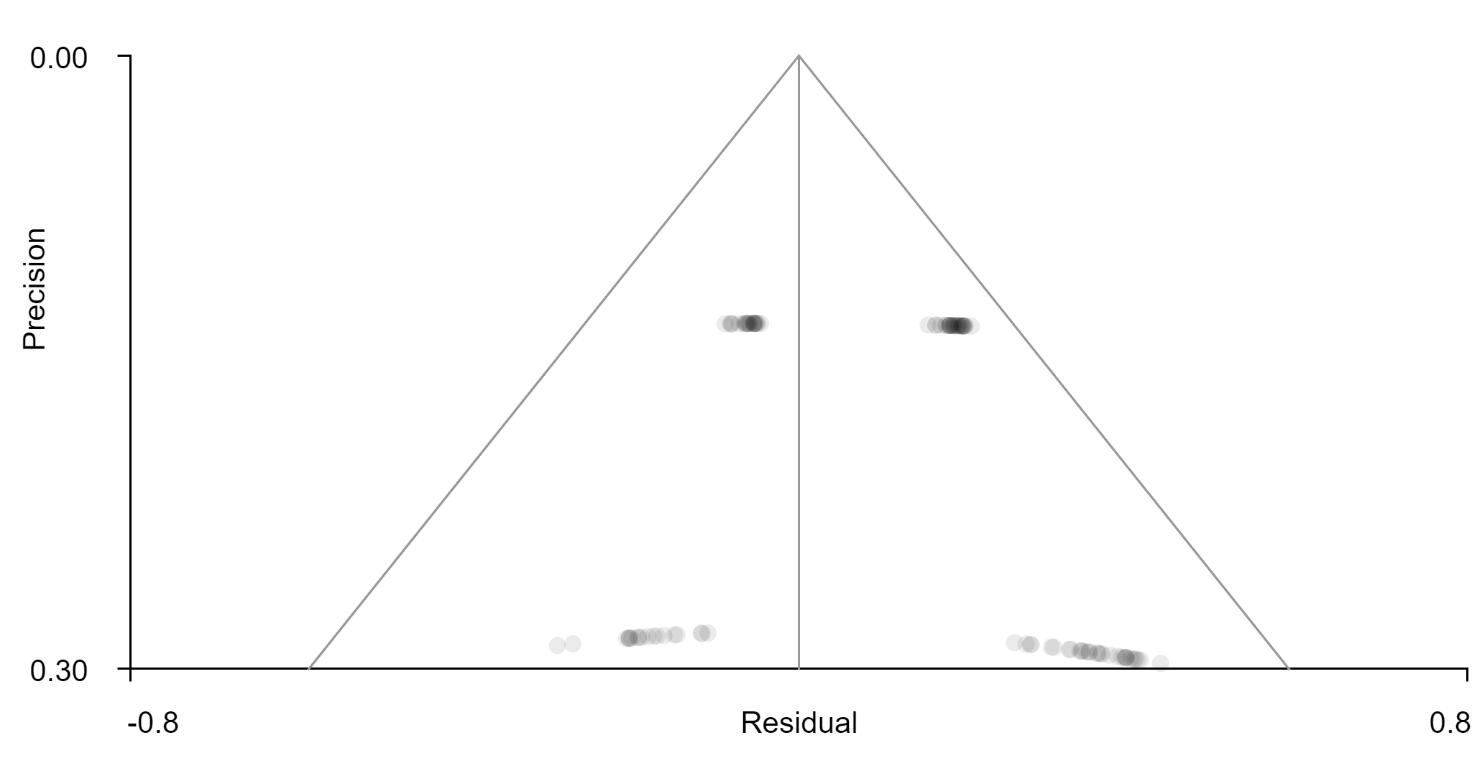
**

**Supplementary Figure 2.** Funnel plots of the meta-analysis in subjects with majority status.

**MNI coordinates: x = -20, y = -76, z = 44**

**
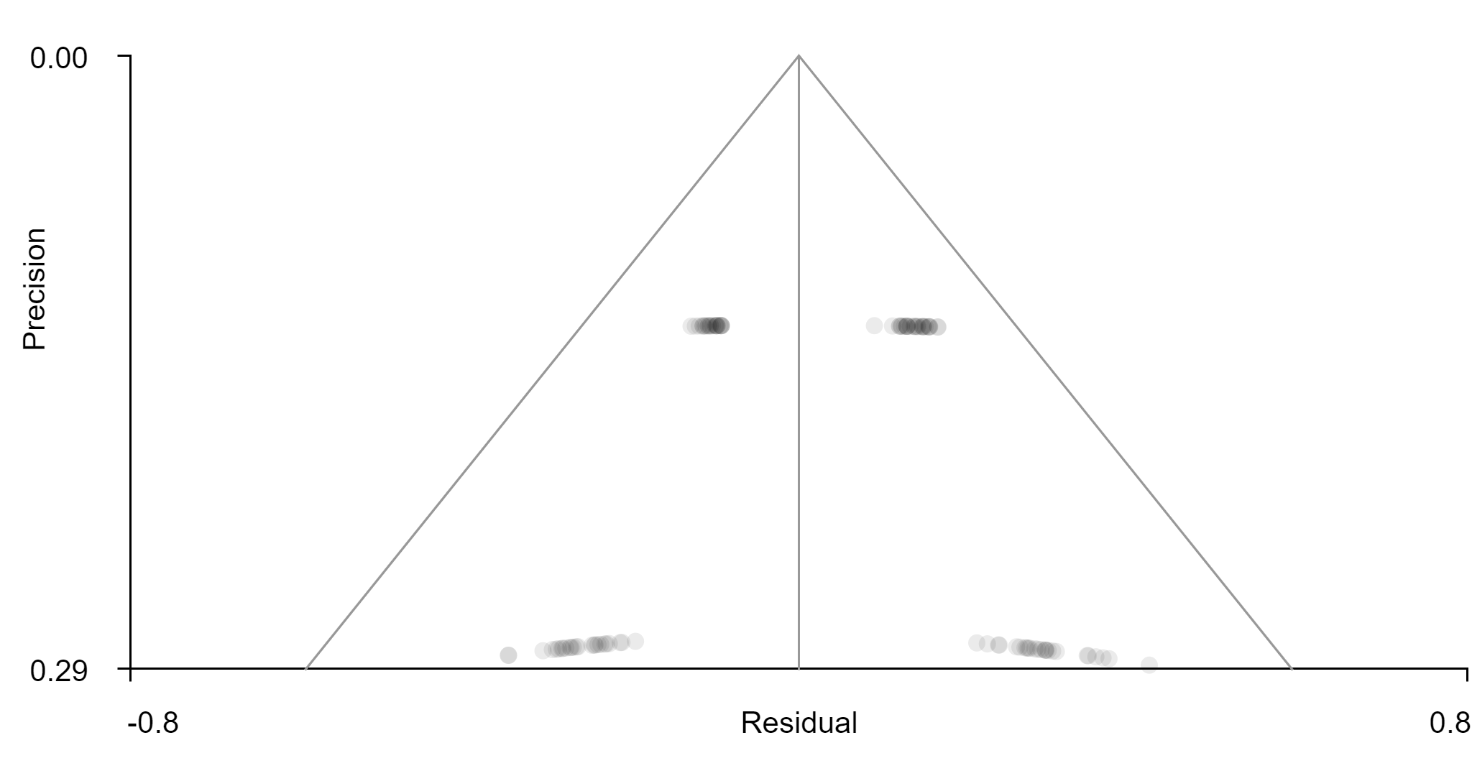
**

**MNI coordinates: x = 18, y = -28, z = -20**

**
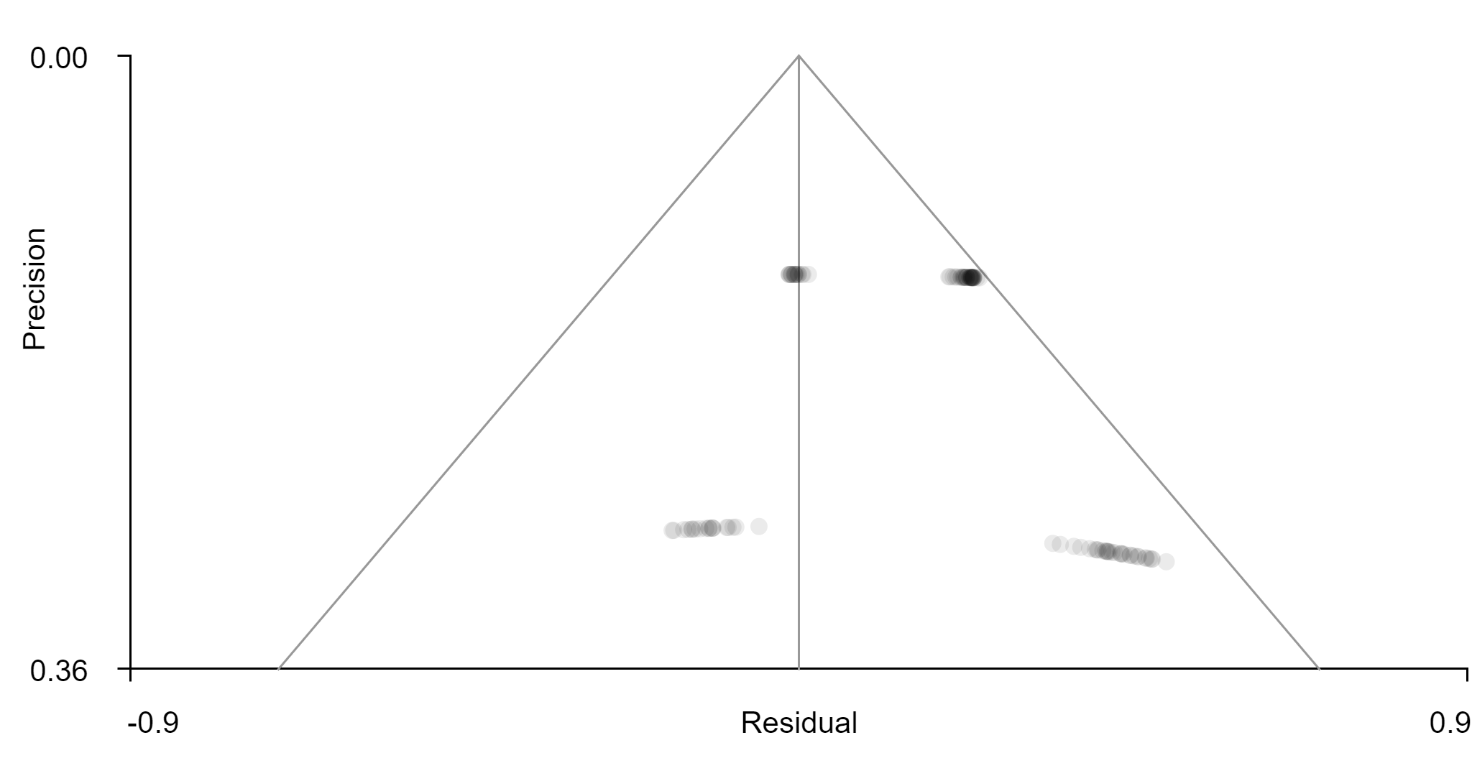
**

**MNI coordinates: x = 50, y = 22, z = 4**

**
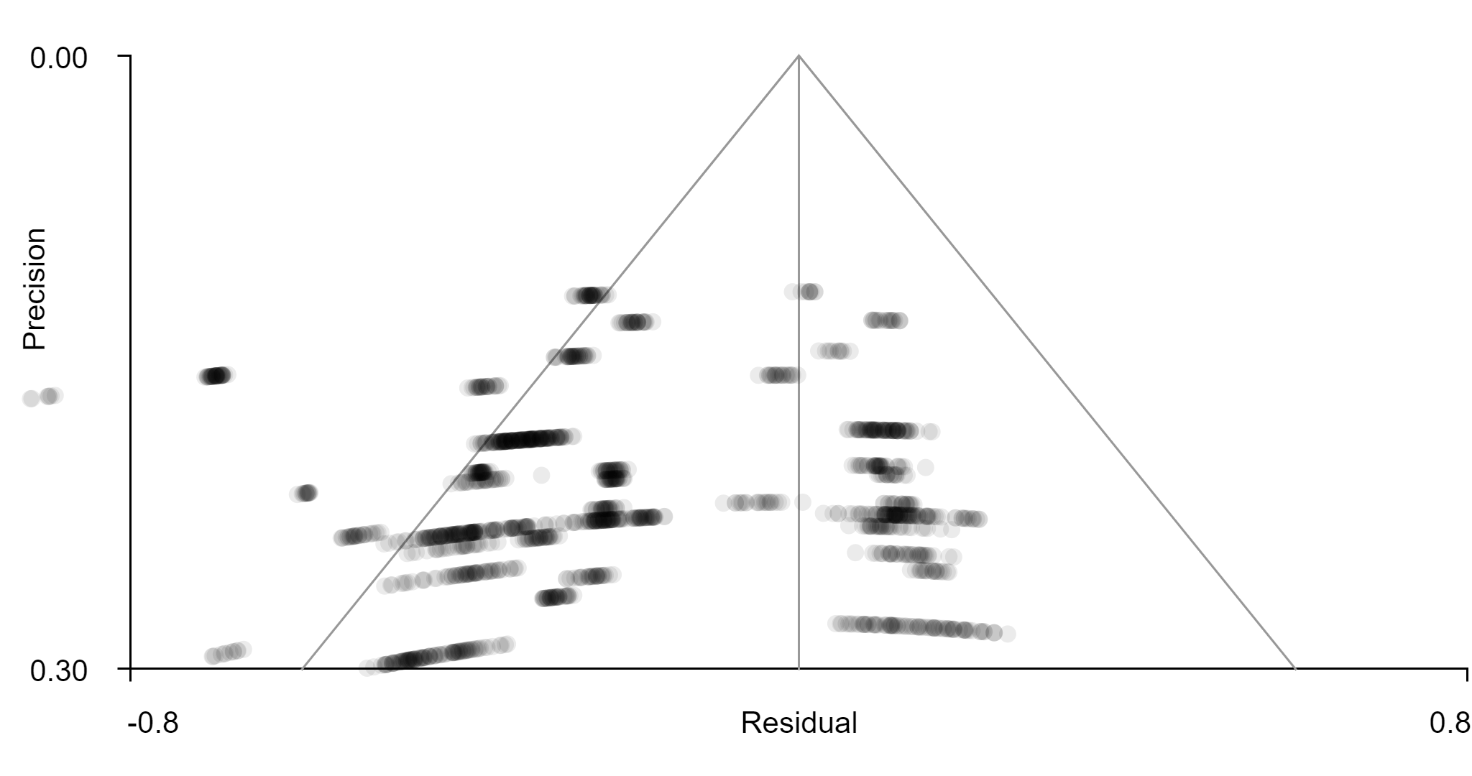
**

**MNI coordinates: x = -30, y = -64, z = -16**


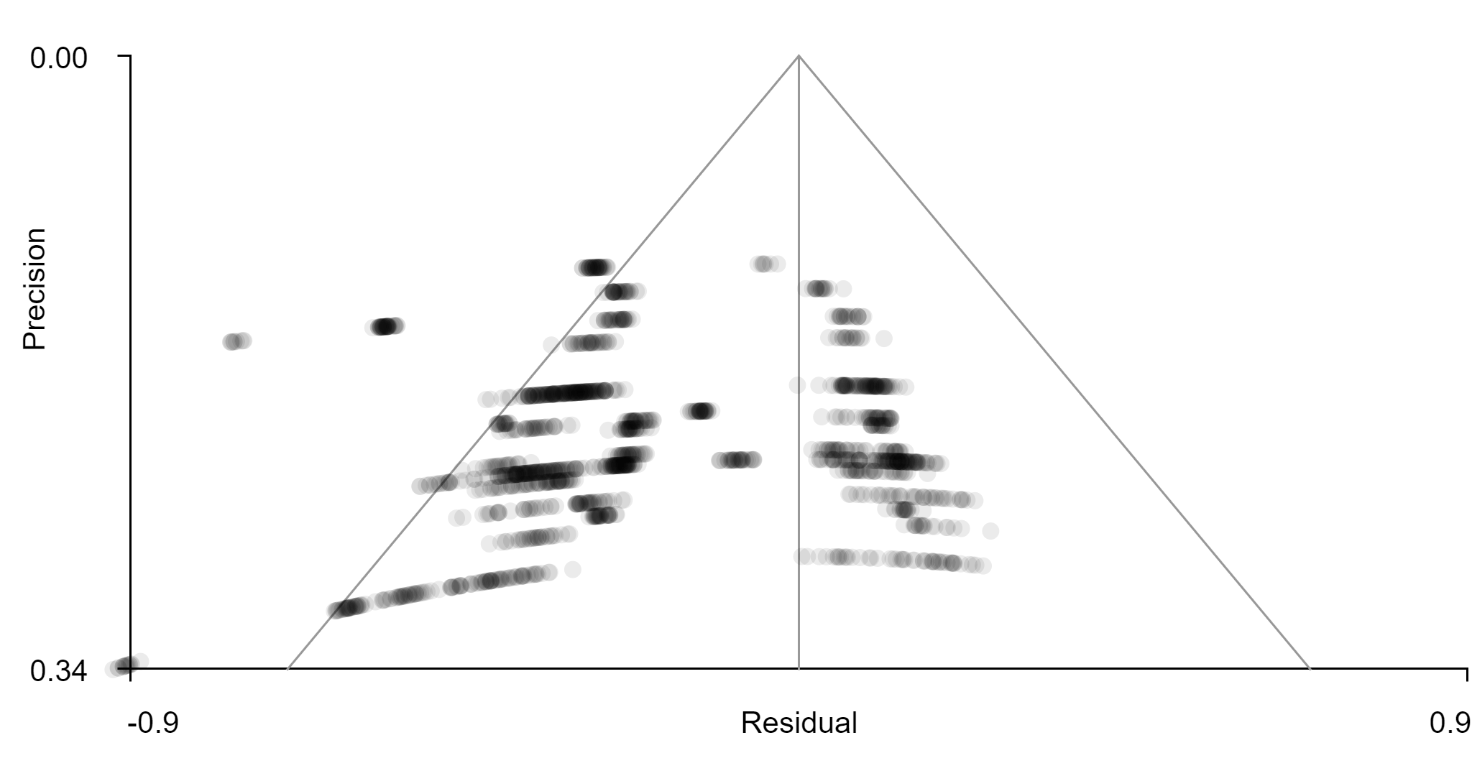


**MNI coordinates: x = 22, y = -96, z = 10**


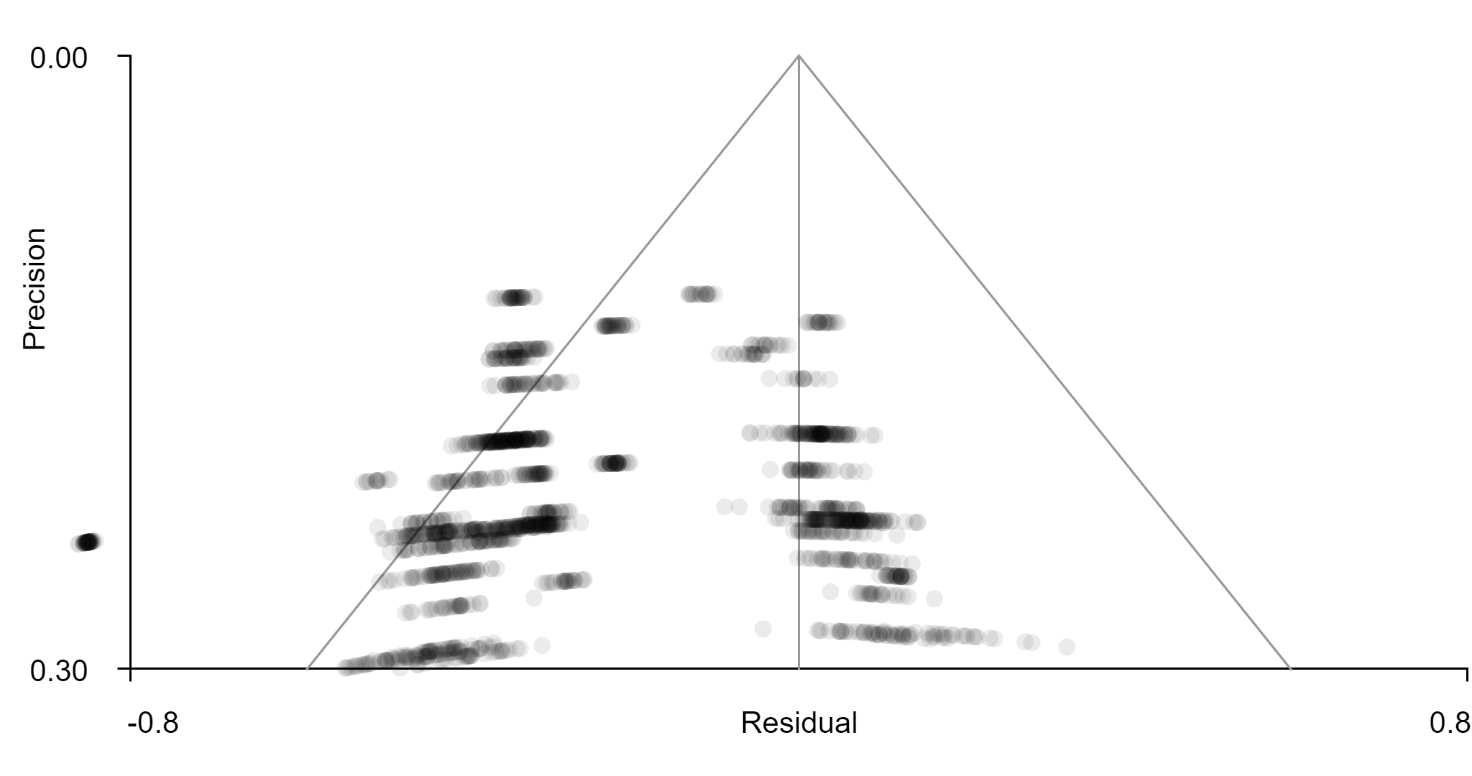


**MNI coordinates: x = 54, y = -58, z = -10**

**
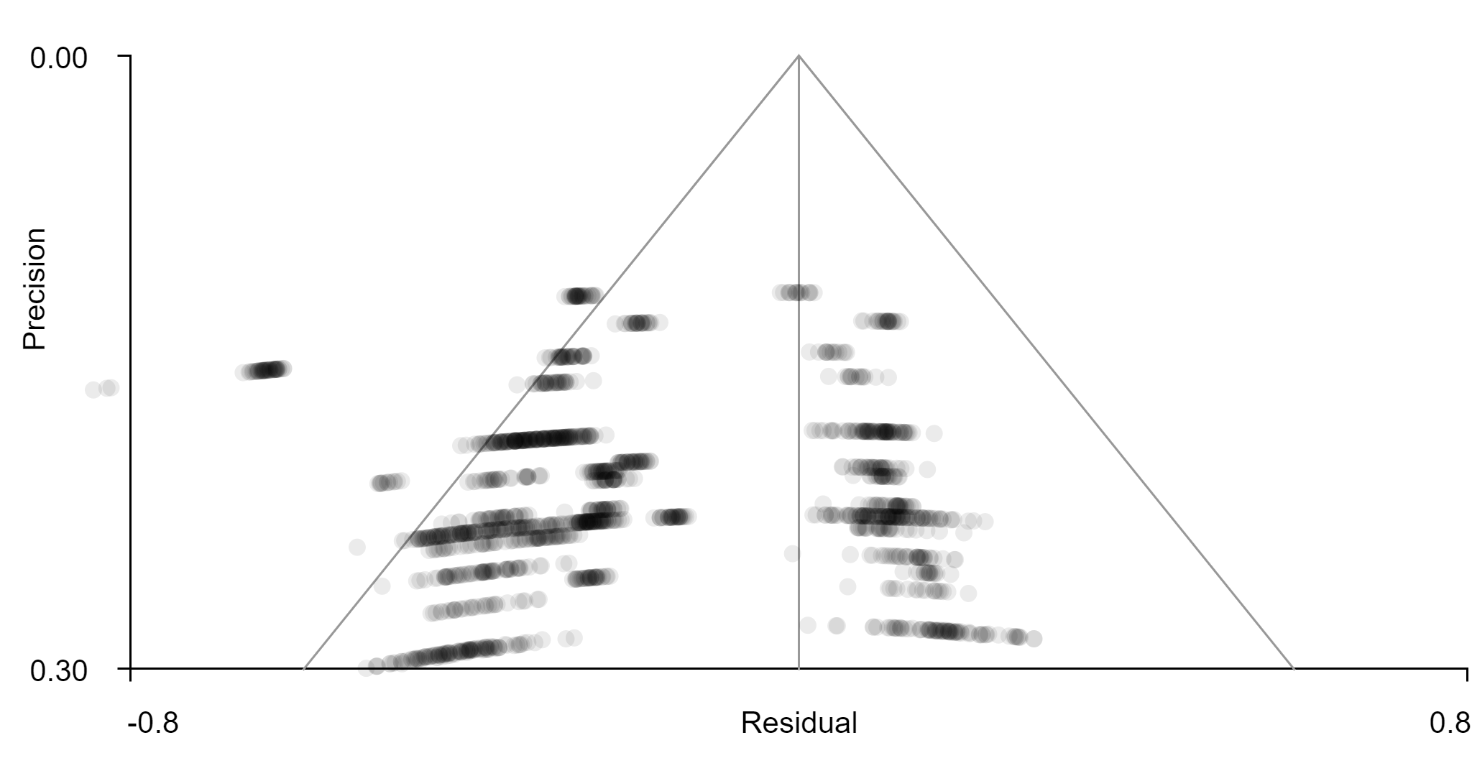
**

**Supplementary Figure 3.** Funnel plots of the meta-analysis on responses toward White target individuals.

**MNI coordinates: x = 2, y = 10, z = 44**

**
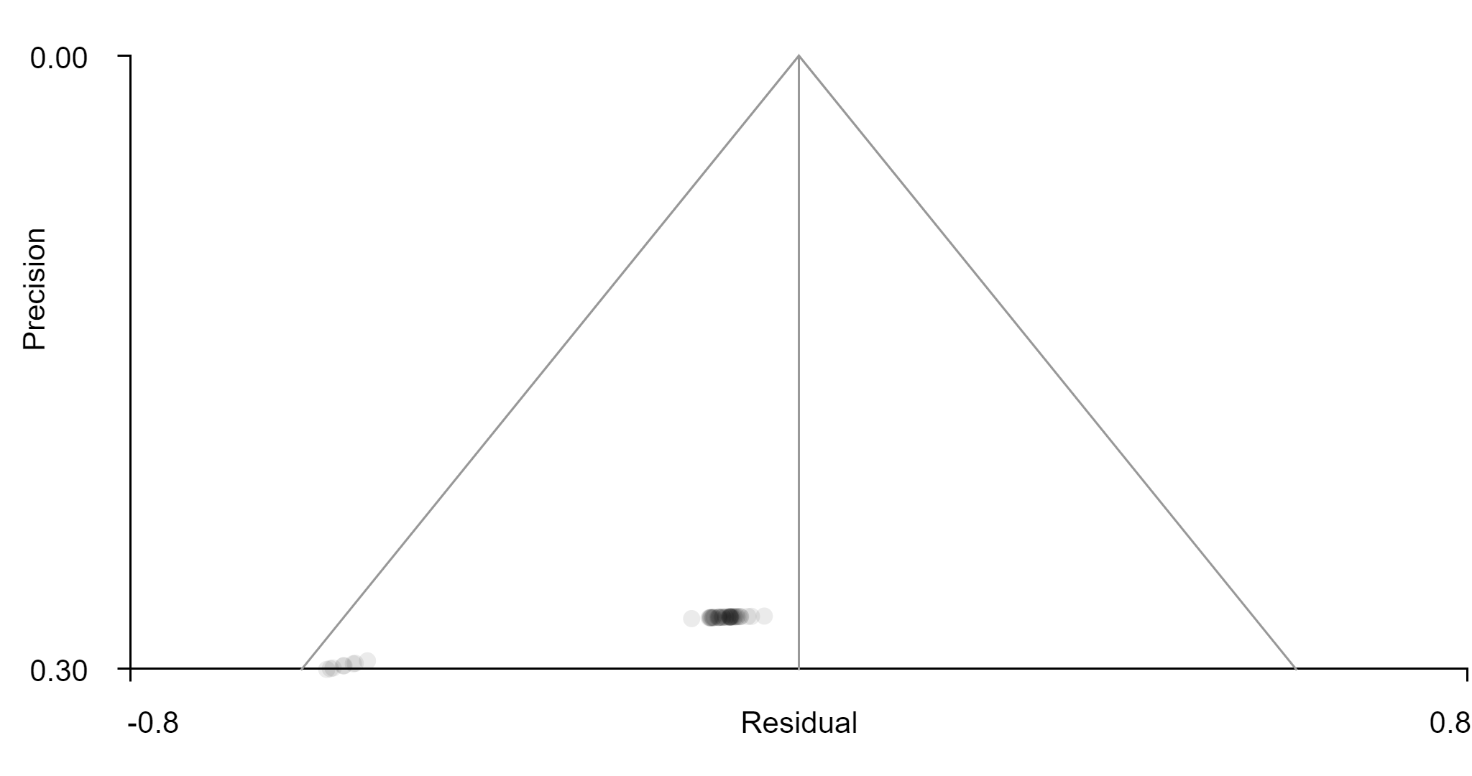
**

**MNI coordinates: x = 10, y = 26, z = 56**

**
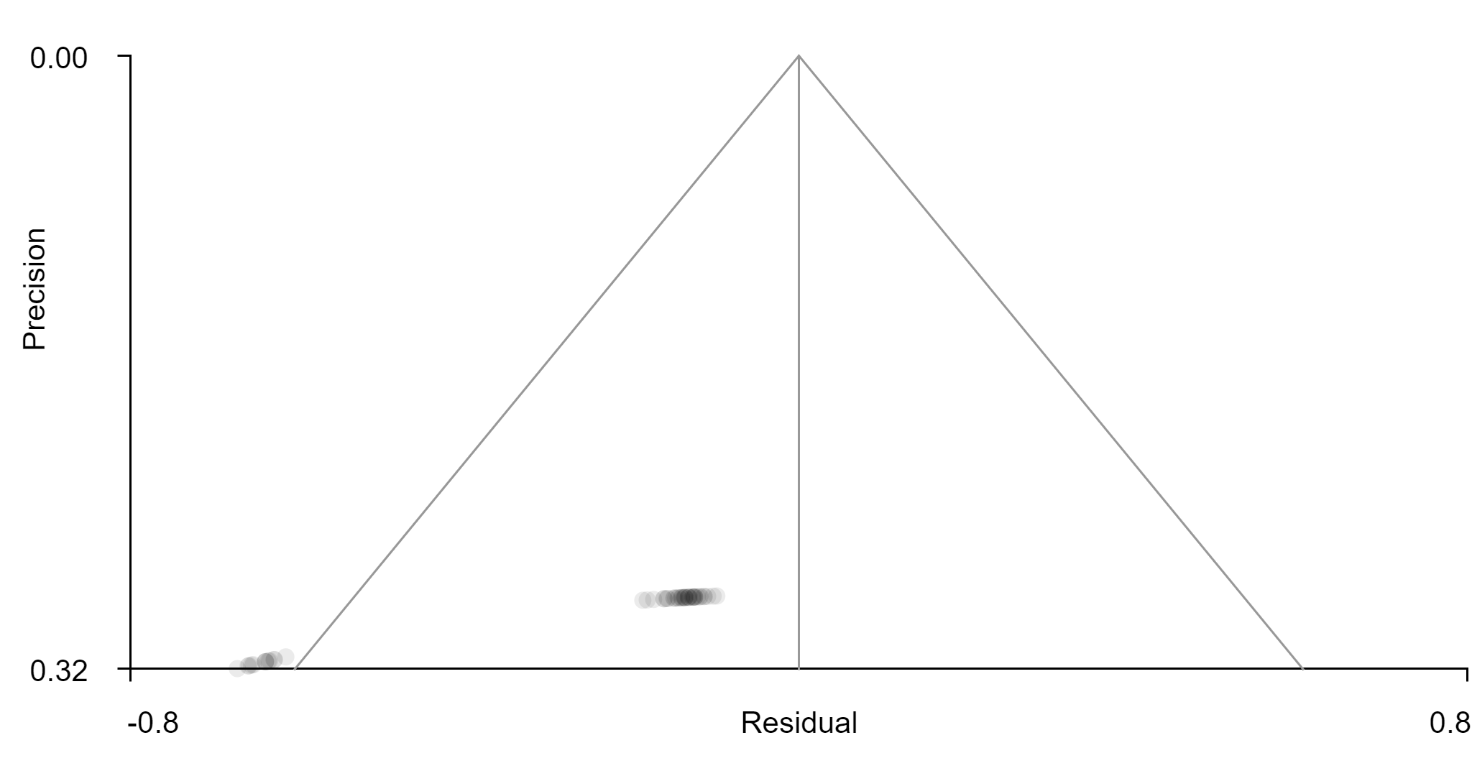
**

**MNI coordinates: x = -10, y = 26, z = 54**

**
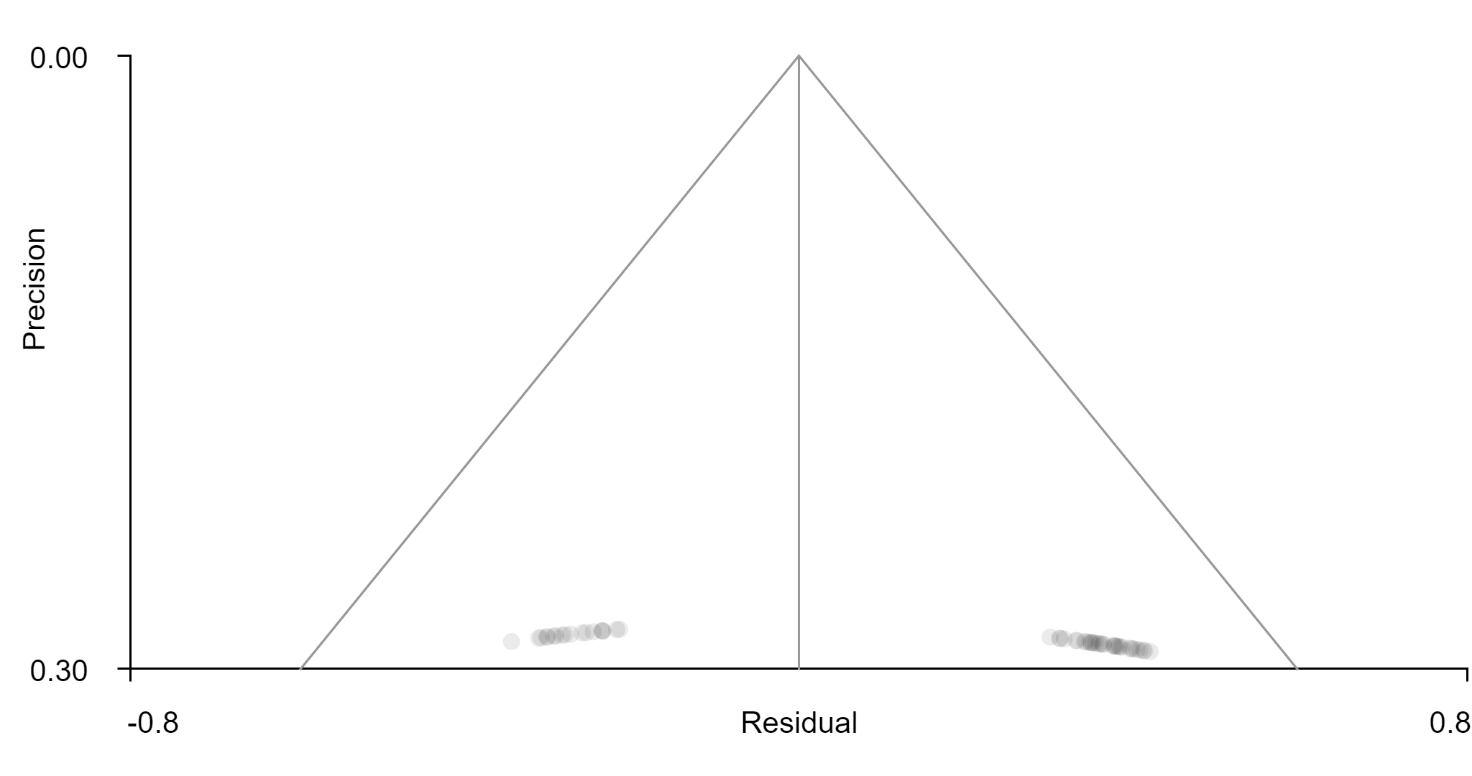
**

**Supplementary Figure 4.** Funnel plots of the meta-analysis on responses toward Black target individuals.

**MNI coordinates: x = -10, y = 8, z = 6**

**
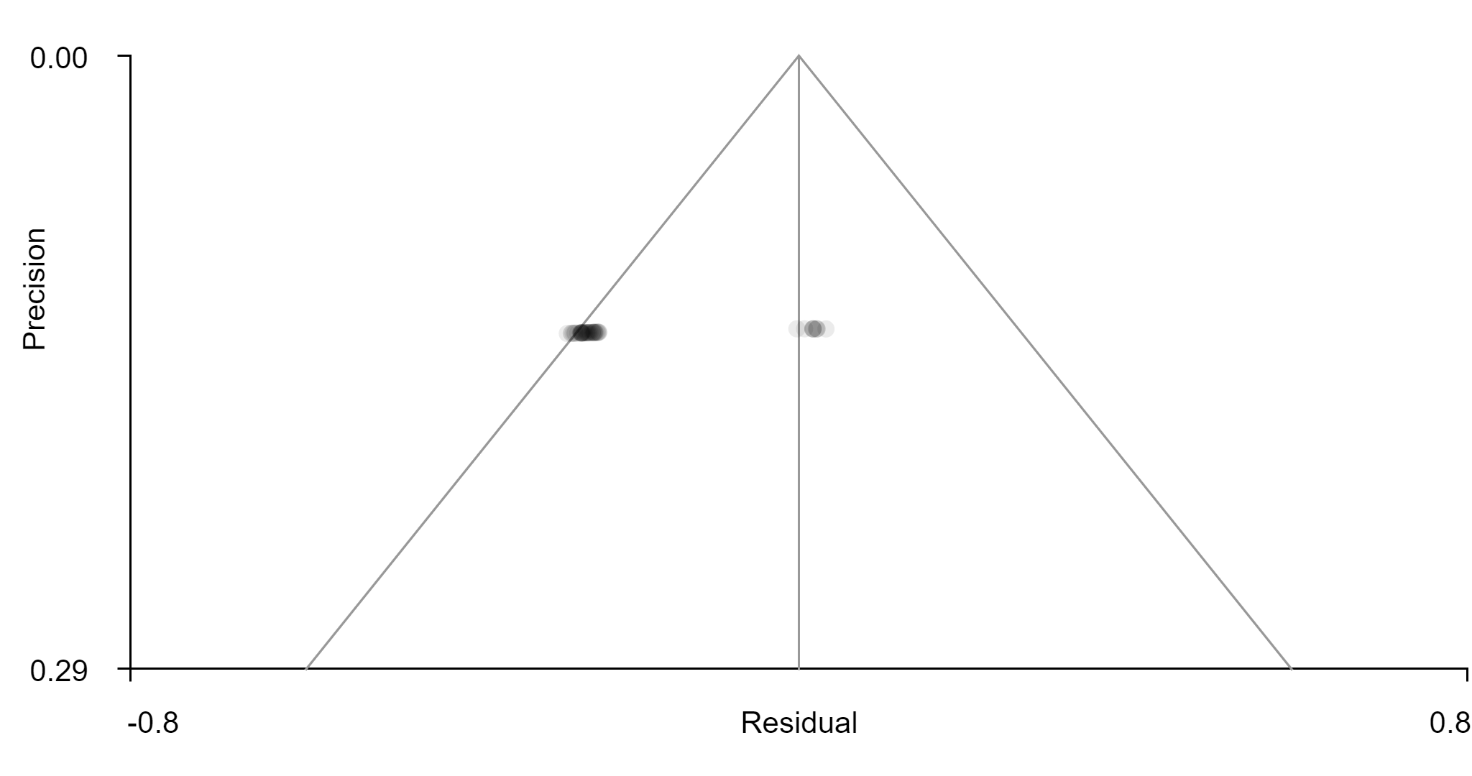
**

**MNI coordinates: x = -4, y = -48, z = 30**

**
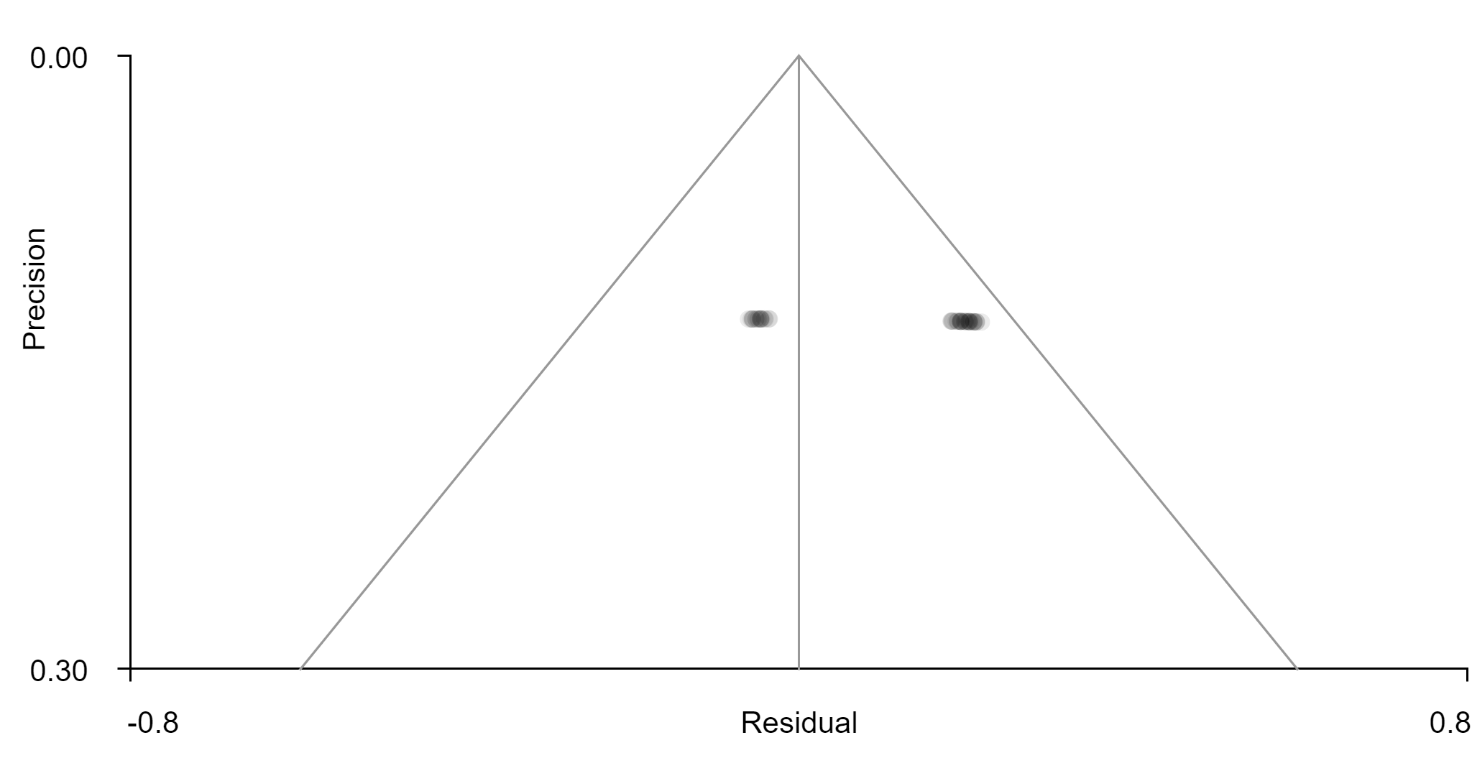
**

**MNI coordinates: x = 20, y = -26, z = -20**

**
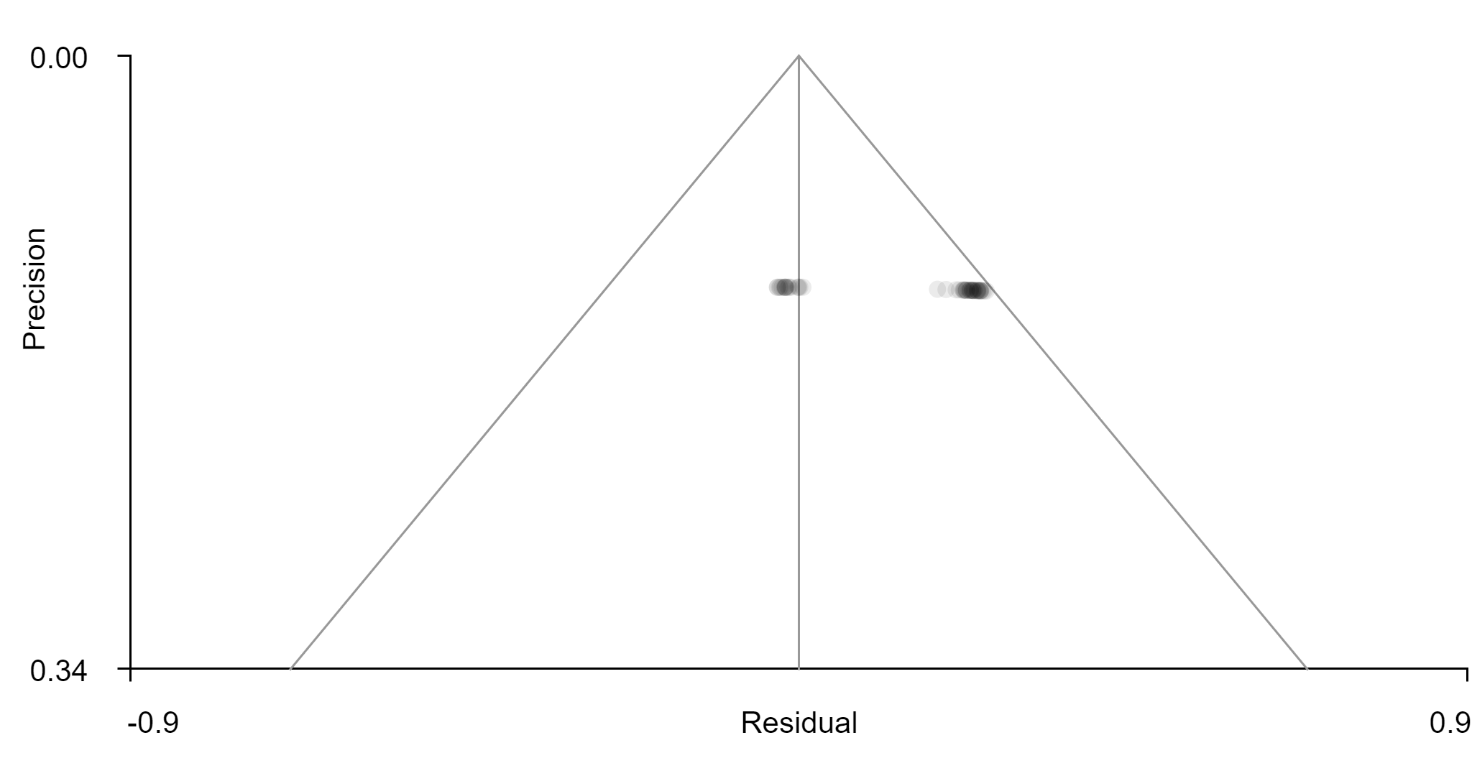
**

**MNI coordinates: x = 48, y = 16, z = -2**

**
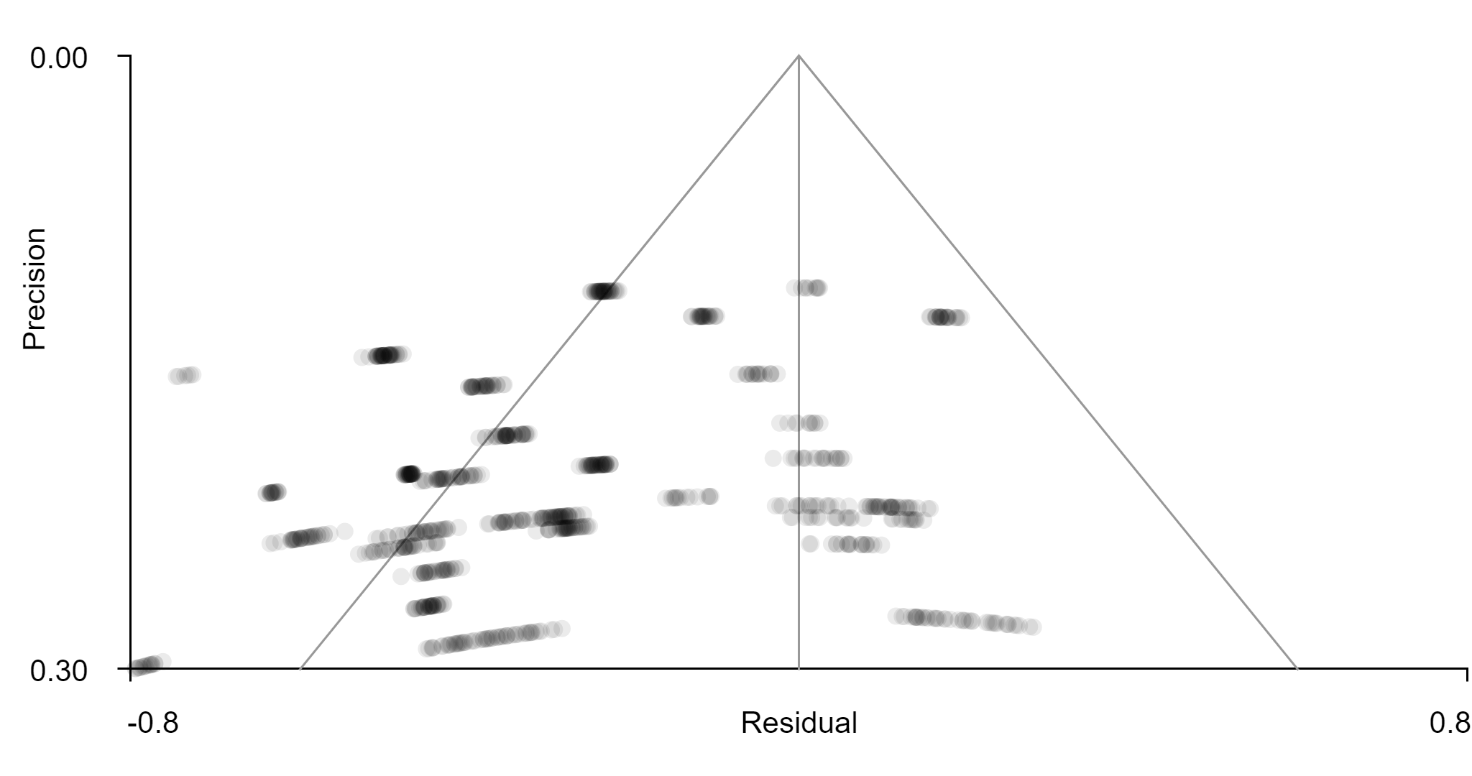
**

**MNI coordinates: x = -20, y = -46, z = -10**

**
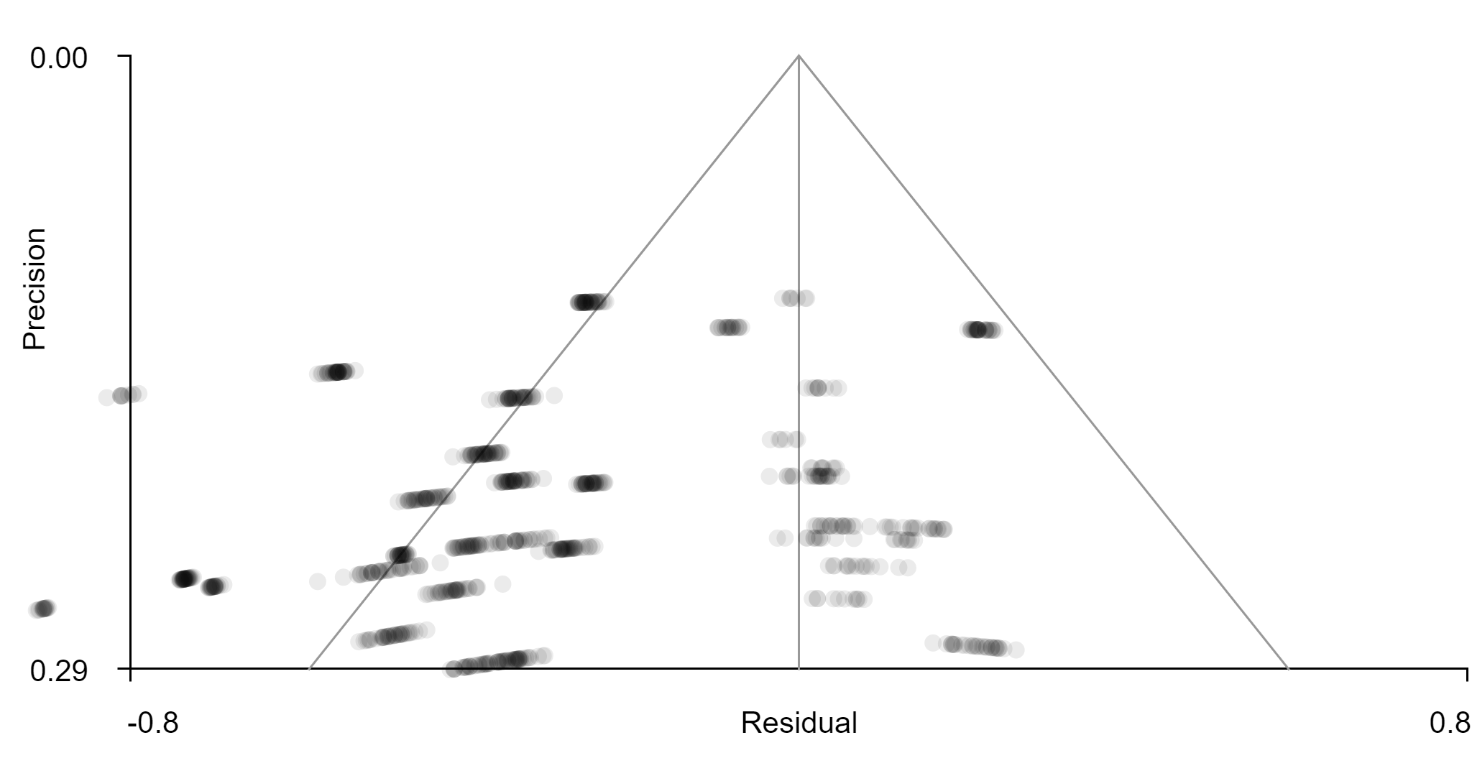
**

**MNI coordinates: x = -2, y = 22, z = 42**

**
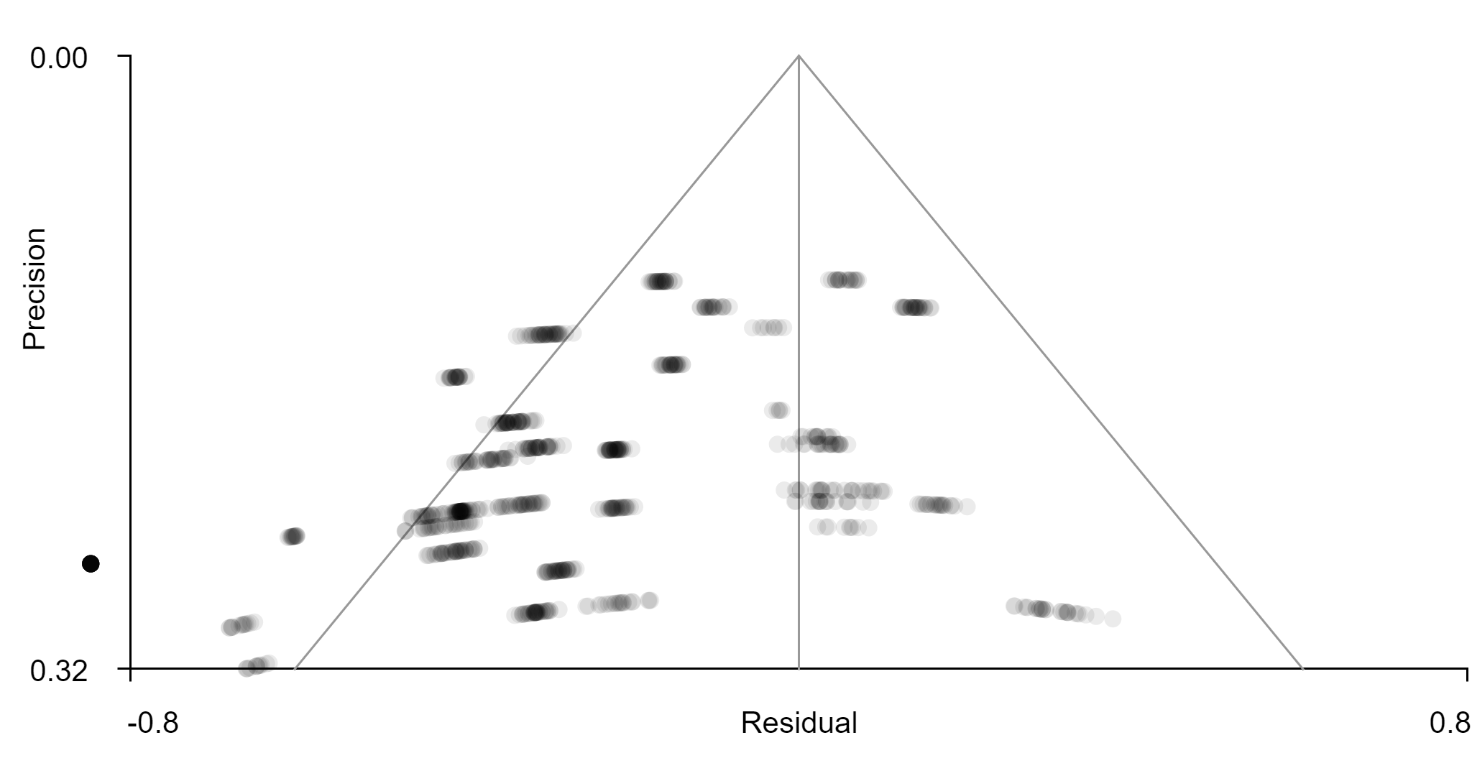
**

**MNI coordinates: x = 30, y = -68, z = -14**

**
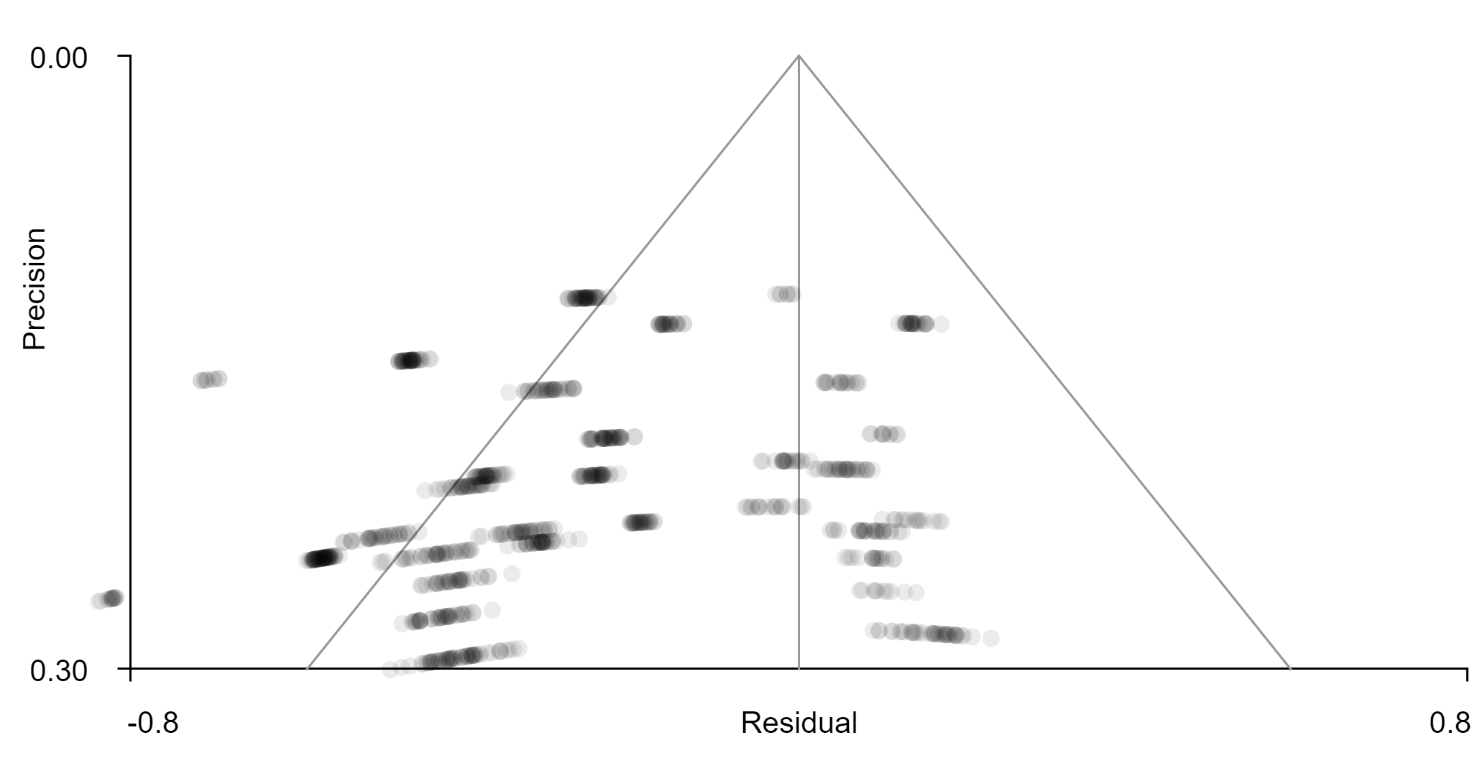
**

**MNI coordinates: x = 54, y = -60, z = -8**

**
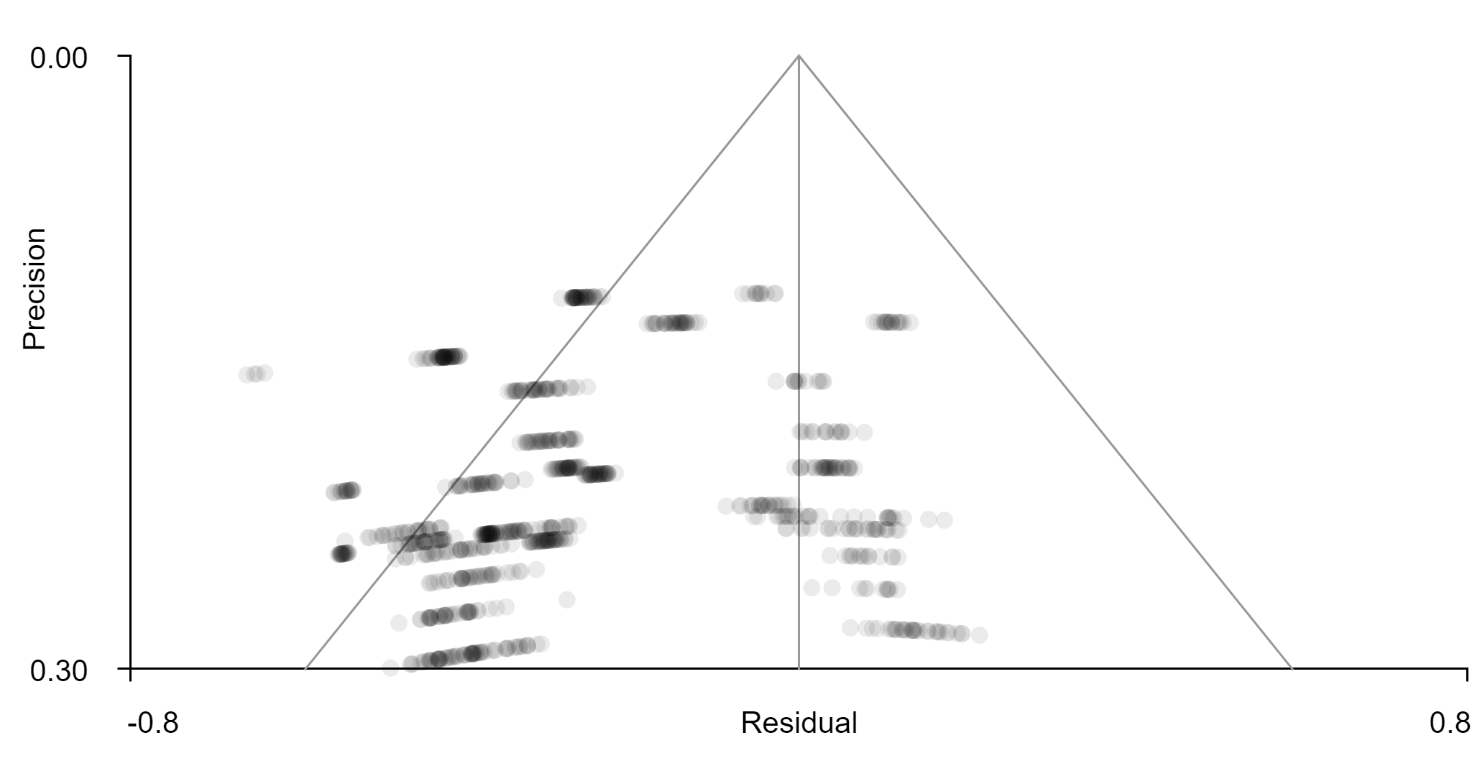
**

**MNI coordinates: x = 54, y = -12, z = 36**

**
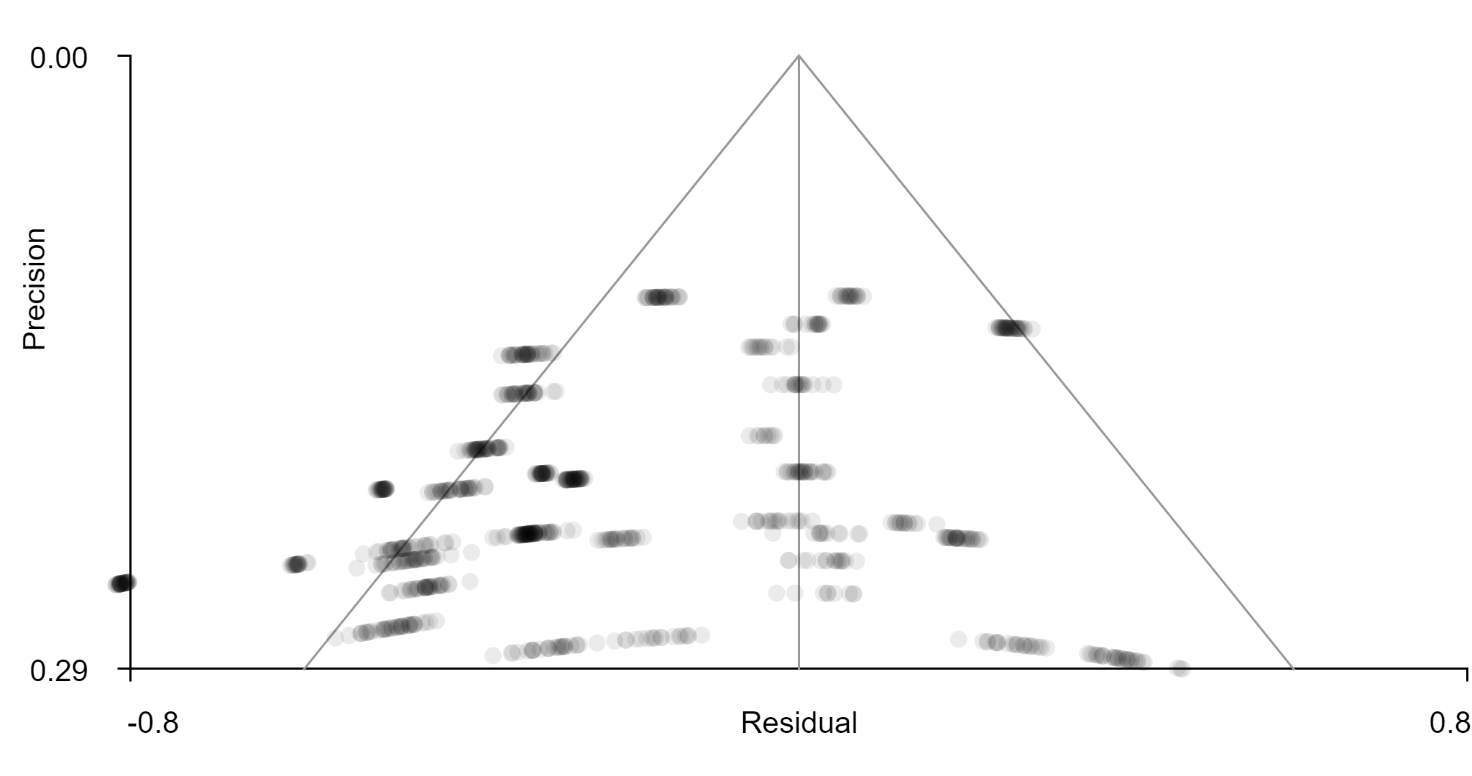
**

**MNI coordinates: x = -40, y = -42, z = 48**

**
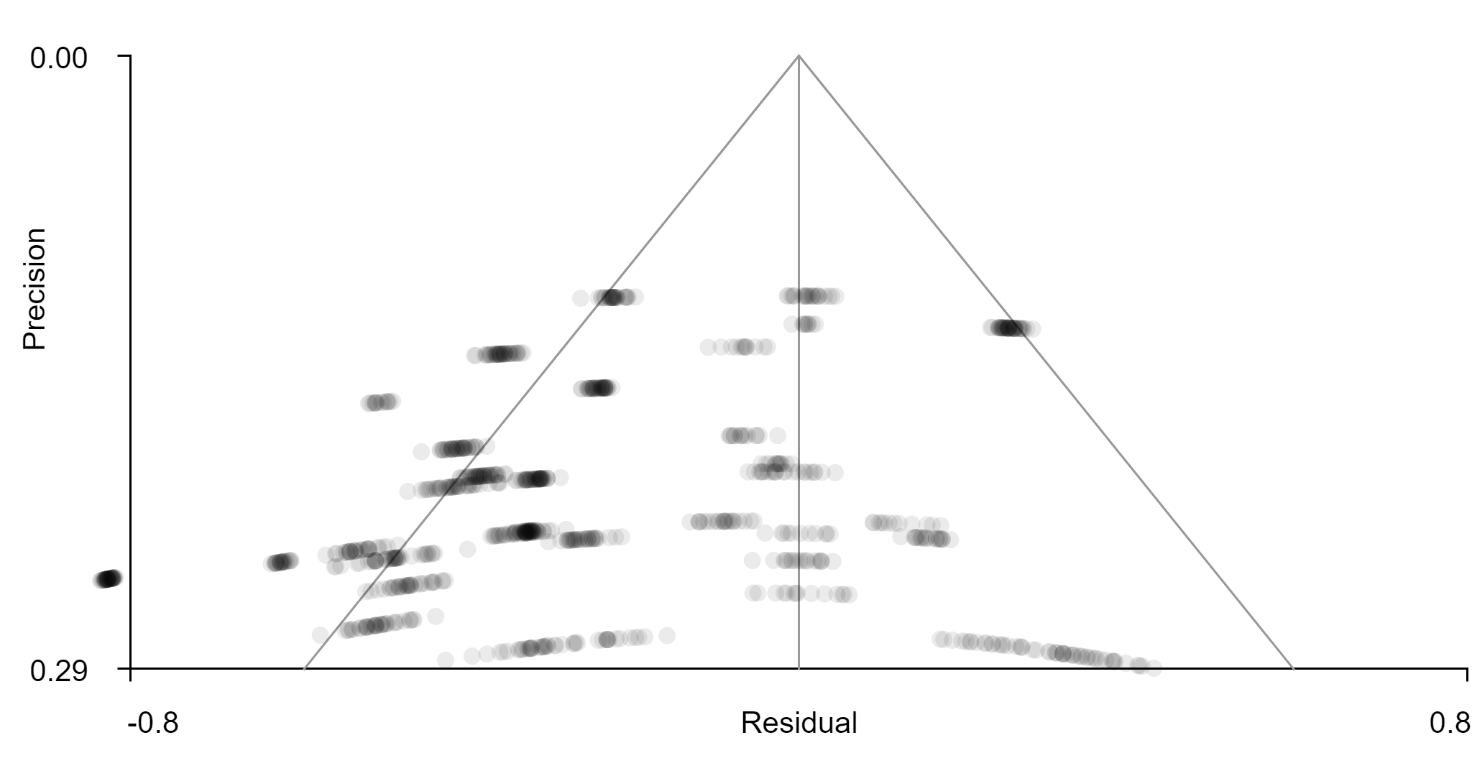
**

**MNI coordinates: x = 0, y = 4, z = 40**

**
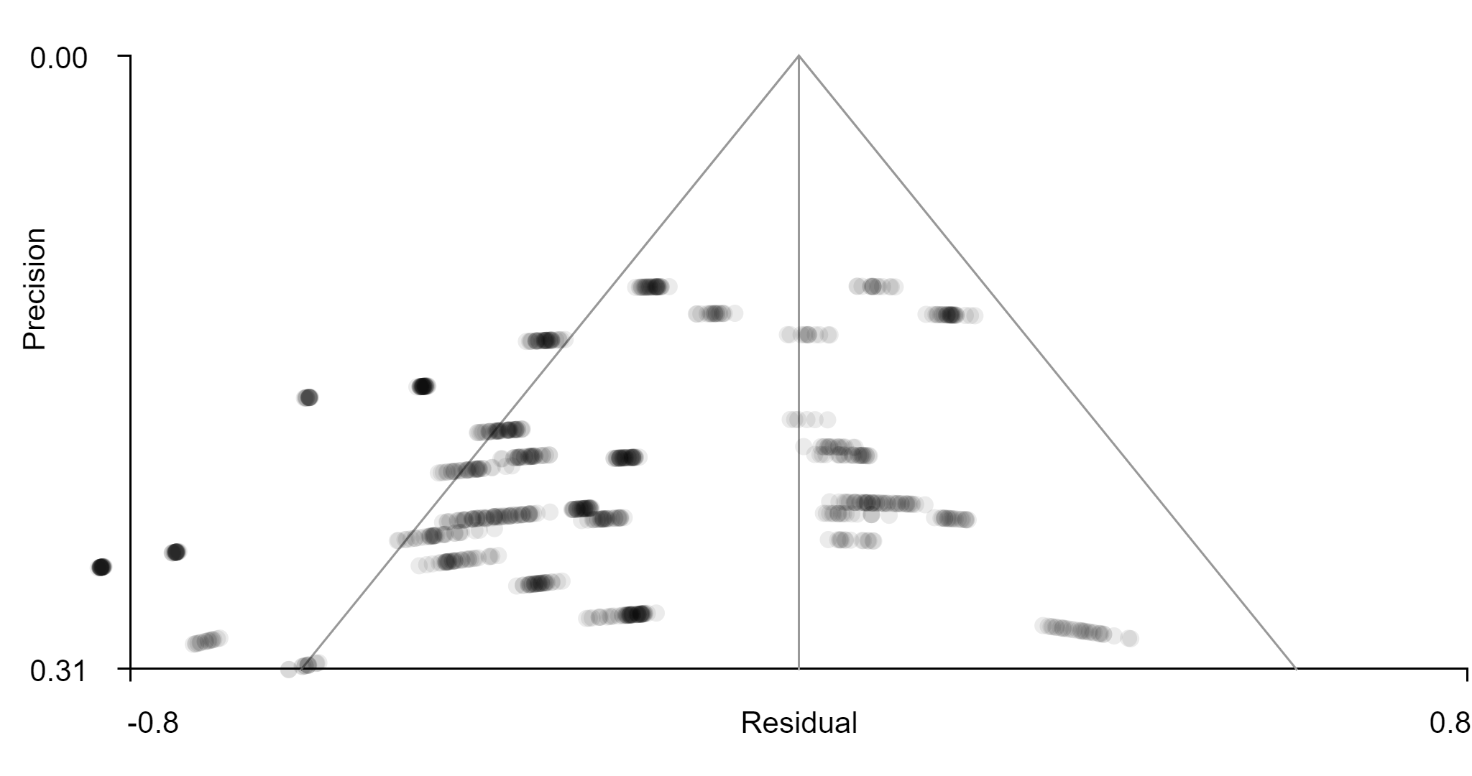
**

**MNI coordinates: x = -36, y = -82, z = 14**

**
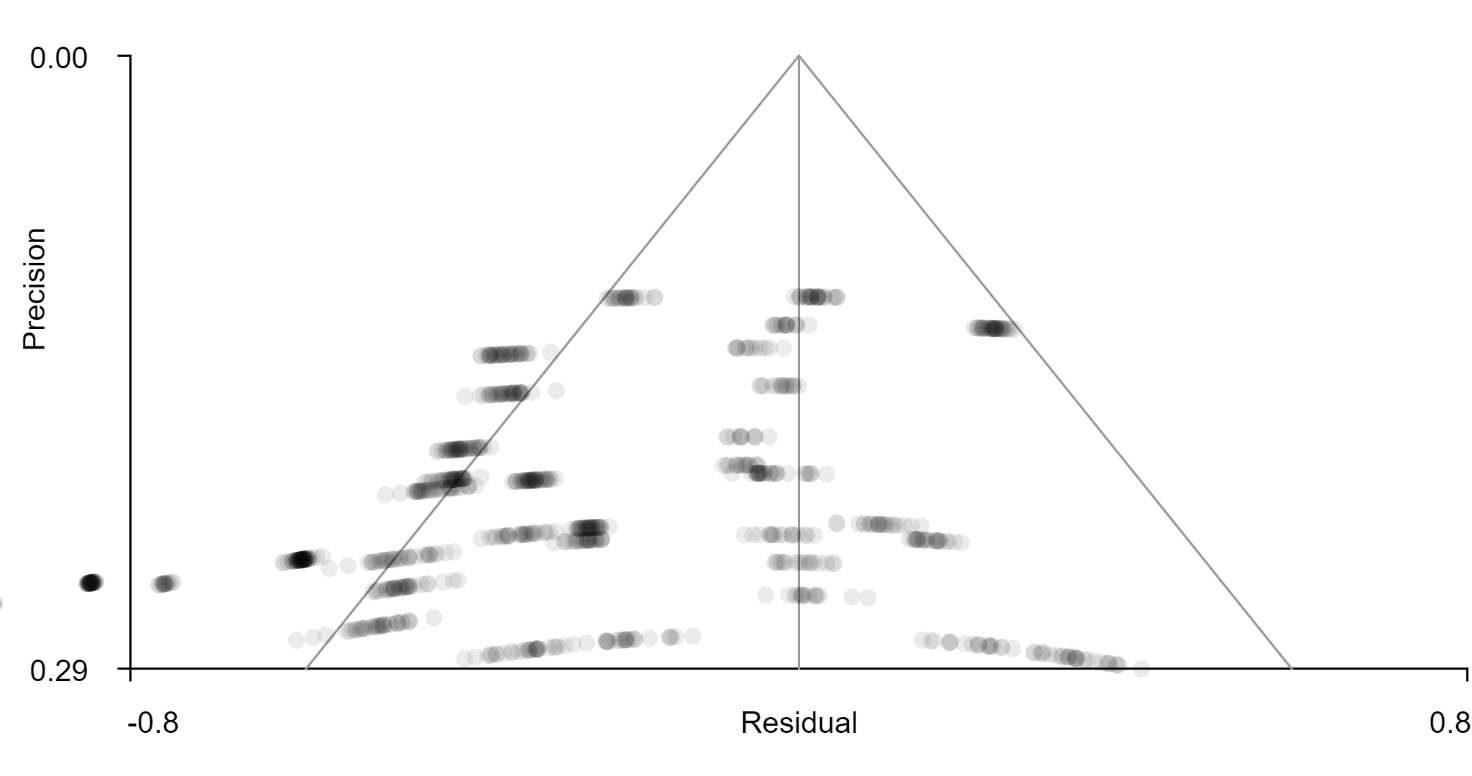
**

**MNI coordinates: x = 32, y = -80, z = -8**

**
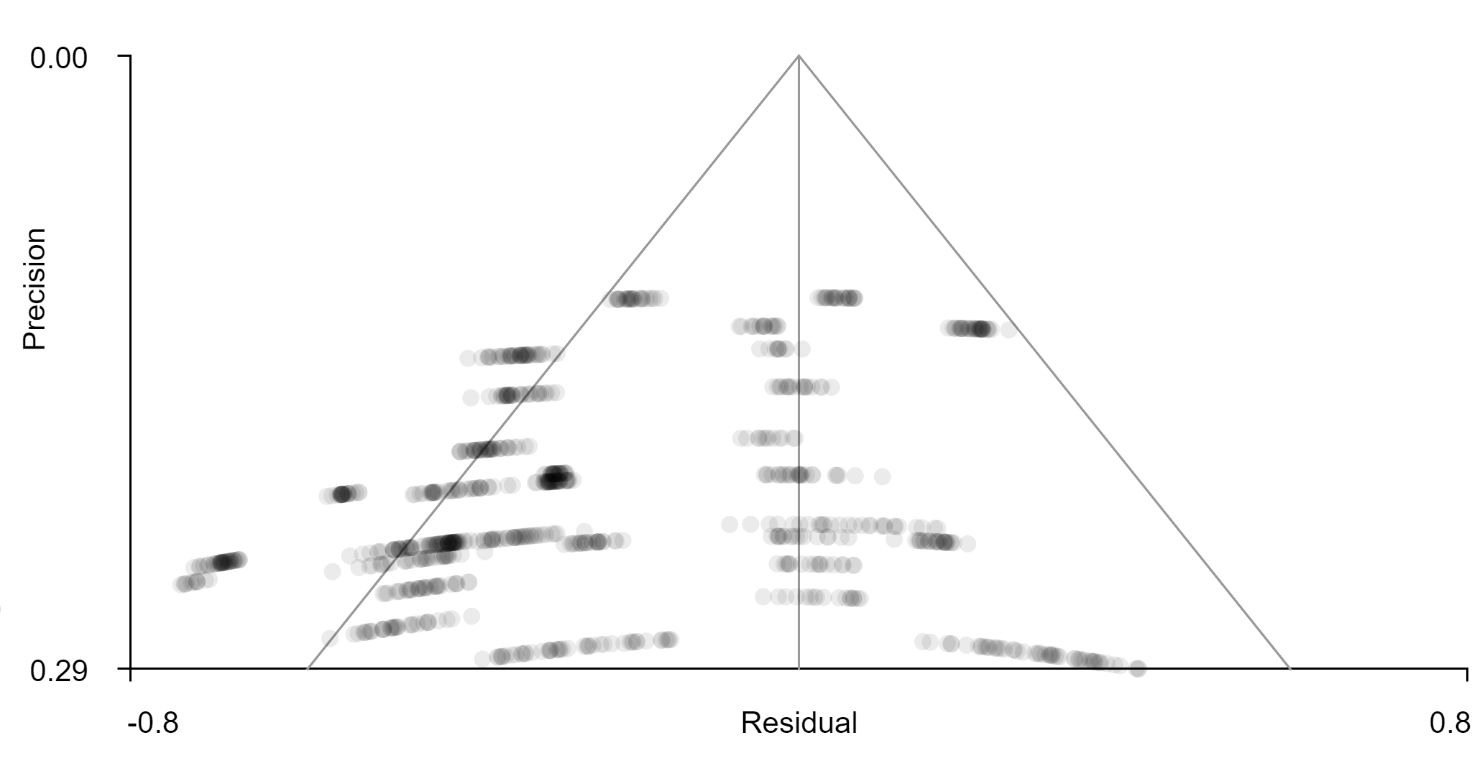
**

**Supplementary Methods.** References of the studies included in the meta-analysis.

Berlingeri, M., Gallucci, M., Danelli, L., Forgiarini, M., Sberna, M., & Paulesu, E. (2016). Guess who's coming to dinner: Brain signatures of racially biased and politically correct behaviors. Neuroscience, 332, 231-241. doi:10.1016/j.neuroscience.2016.06.048

Cao, Y., Contreras-Huerta, L. S., McFadyen, J., & Cunnington, R. (2015). Racial bias in neural response to others' pain is reduced with other-race contact. Cortex, 70, 68-78. doi:10.1016/j.cortex.2015.02.010

Cassidy, B. S., Hughes, C., & Krendl, A. C. (2020). A stronger relationship between reward responsivity and trustworthiness evaluations emerges in healthy aging. Neuropsychol Dev Cogn B Aging Neuropsychol Cogn, 1-18. doi:10.1080/13825585.2020.1809630

Chen, P. A., Whalen, P. J., Freeman, J. B., Taylor, J. M., & Heatherton, T. F. (2015). Brain reward activity to masked in-group smiling faces predicts friendship development Soc Psychol Personal Sci, 8(4), 415-421. doi:10.1177/1948550614566093

Contreras-Huerta, L. S., Baker, K. S., Reynolds, K. J., Batalha, L., & Cunnington, R. (2013). Racial bias in neural empathic responses to pain. PLoS One, 8(12), e84001. doi:10.1371/journal.pone.0084001

Contreras, J. M., Banaji, M. R., & Mitchell, J. P. (2013). Multivoxel patterns in fusiform face area differentiate faces by sex and race. PLoS One, 8(7), e69684. doi:10.1371/journal.pone.0069684

Cunningham, W. A., Johnson, M. K., Raye, C. L., Chris Gatenby, J., Gore, J. C., & Banaji, M. R. (2004). Separable neural components in the processing of black and white faces. Psychol Sci, 15(12), 806-813. doi:10.1111/j.0956-7976.2004.00760.x

Domínguez, D. J., van Nunspeet, F., Gupta, A., Eres, R., Louis, W. R., Decety, J., & Molenberghs, P. (2018). Lateral orbitofrontal cortex activity is modulated by group membership in situations of justified and unjustified violence. Soc Neurosci, 13(6), 739-755. doi:10.1080/17470919.2017.1392342

Farmer, H., Hewstone, M., Spiegler, O., Morse, H., Saifullah, A., Pan, X., . . . Terbeck, S. (2020). Positive intergroup contact modulates fusiform gyrus activity to black and white faces. Sci Rep, 10(1), 2700. doi:10.1038/s41598-020-59633-9

Feng, L., Liu, J., Wang, Z., Li, J., Li, L., Ge, L., . . . Lee, K. (2011). The other face of the other-race effect: an fMRI investigation of the other-race face categorization advantage. Neuropsychologia, 49(13), 3739-3749. doi:10.1016/j.neuropsychologia.2011.09.031

Firat, R. B., Hitlin, S., Magnotta, V., & Tranel, D. (2017). Putting race in context: social class modulates processing of race in the ventromedial prefrontal cortex and amygdala. Soc Cogn Affect Neurosci, 12(8), 1314-1324. doi:10.1093/scan/nsx052

Fourie, M. M., Stein, D. J., Solms, M., Gobodo-Madikizela, P., & Decety, J. (2017). Empathy and moral emotions in post-apartheid South Africa: an fMRI investigation. Soc Cogn Affect Neurosci, 12(6), 881-892. doi:10.1093/scan/nsx019

Freeman, J. B., Schiller, D., Rule, N. O., & Ambady, N. (2010). The neural origins of superficial and individuated judgments about ingroup and outgroup members. Hum Brain Mapp, 31(1), 150-159. doi:10.1002/hbm.20852

Gilbert, S. J., Swencionis, J. K., & Amodio, D. M. (2012). Evaluative vs. trait representation in intergroup social judgments: distinct roles of anterior temporal lobe and prefrontal cortex. Neuropsychologia, 50(14), 3600-3611. doi:10.1016/j.neuropsychologia.2012.09.002

Katsumi, Y., & Dolcos, S. (2017). Neural Correlates of Racial Ingroup Bias in Observing Computer-Animated Social Encounters. Front Hum Neurosci, 11, 632. doi:10.3389/fnhum.2017.00632

Krosch, A. R., & Amodio, D. M. (2019). Scarcity disrupts the neural encoding of Black faces: A socioperceptual pathway to discrimination. J Pers Soc Psychol, 117(5), 859-875. doi:10.1037/pspa0000168

Lee, K. U., Khang, H. S., Kim, K. T., Kim, Y. J., Kweon, Y. S., Shin, Y. W., ... & Liberzon, I. (2008). Distinct processing of facial emotion of own-race versus other-race. Neuroreport, 19(10), 1021-1025.

Li, X., Liu, Y., Luo, S., Wu, B., Wu, X., & Han, S. (2015). Mortality salience enhances racial in-group bias in empathic neural responses to others' suffering. Neuroimage, 118, 376-385. doi:10.1016/j.neuroimage.2015.06.023

Lin, L. C., Qu, Y., & Telzer, E. H. (2018). Intergroup social influence on emotion processing in the brain. Proc Natl Acad Sci U S A, 115(42), 10630-10635. doi:10.1073/pnas.1802111115

Liu, Y., Lin, W., Xu, P., Zhang, D., & Luo, Y. (2015). Neural basis of disgust perception in racial prejudice. Hum Brain Mapp, 36(12), 5275-5286. doi:10.1002/hbm.23010

Losin, E. A., Iacoboni, M., Martin, A., Cross, K. A., & Dapretto, M. (2012). Race modulates neural activity during imitation. Neuroimage, 59(4), 3594-3603. doi:10.1016/j.neuroimage.2011.10.074

Luo, S., Li, B., Ma, Y., Zhang, W., Rao, Y., & Han, S. (2015). Oxytocin receptor gene and racial ingroup bias in empathy-related brain activity. Neuroimage, 110, 22-31. doi:10.1016/j.neuroimage.2015.01.042

Mathur, V. A., Harada, T., Lipke, T., & Chiao, J. Y. (2010). Neural basis of extraordinary empathy and altruistic motivation. Neuroimage, 51(4), 1468-1475. doi:10.1016/j.neuroimage.2010.03.025

Mattan, B. D., Kubota, J. T., Dang, T. P., & Cloutier, J. (2018). External motivation to avoid prejudice alters neural responses to targets varying in race and status. Soc Cogn Affect Neurosci, 13(1), 22-31. doi:10.1093/scan/nsx128

McCutcheon, R., Bloomfield, M. A. P., Dahoun, T., Quinlan, M., Terbeck, S., Mehta, M., & Howes, O. (2018). Amygdala reactivity in ethnic minorities and its relationship to the social environment: an fMRI study. Psychol Med, 48(12), 1985-1992. doi:10.1017/s0033291717003506

Molapour, T., Golkar, A., Navarrete, C. D., Haaker, J., & Olsson, A. (2015). Neural correlates of biased social fear learning and interaction in an intergroup context. Neuroimage, 121, 171-183. doi:10.1016/j.neuroimage.2015.07.015

Rauchbauer, B., Majdandžić, J., Hummer, A., Windischberger, C., & Lamm, C. (2015). Distinct neural processes are engaged in the modulation of mimicry by social group-membership and emotional expressions. Cortex, 70, 49-67. doi:10.1016/j.cortex.2015.03.007

Richeson, J. A., Baird, A. A., Gordon, H. L., Heatherton, T. F., Wyland, C. L., Trawalter, S., & Shelton, J. N. (2003). An fMRI investigation of the impact of interracial contact on executive function. Nat Neurosci, 6(12), 1323-1328. doi:10.1038/nn1156

Rubien-Thomas, E., Berrian, N., Cervera, A., Nardos, B., Cohen, A. O., Lowrey, A., ... & Casey, B. J. (2021). Processing of Task-Irrelevant Race Information is Associated with Diminished Cognitive Control in Black and White Individuals. Cogn Affect Behav Neurosci, 21(3), 625-638.

Rule, N. O., Freeman, J. B., Moran, J. M., Gabrieli, J. D., Adams, R. B., Jr., & Ambady, N. (2010). Voting behavior is reflected in amygdala response across cultures. Soc Cogn Affect Neurosci, 5(2-3), 349-355. doi:10.1093/scan/nsp046

Sheng, F., Liu, Q., Li, H., Fang, F., & Han, S. (2014). Task modulations of racial bias in neural responses to others' suffering. Neuroimage, 88, 263-270. doi:10.1016/j.neuroimage.2013.10.017

Telzer, E. H., Ichien, N., & Qu, Y. (2015). The ties that bind: Group membership shapes the neural correlates of in-group favoritism. Neuroimage, 115, 42-51. doi:10.1016/j.neuroimage.2015.04.035

Van Bavel, J. J., Packer, D. J., & Cunningham, W. A. (2008). The neural substrates of in-group bias: a functional magnetic resonance imaging investigation. Psychol Sci, 19(11), 1131-1139. doi:10.1111/j.1467-9280.2008.02214.x

Wang, C., Wu, B., Liu, Y., Wu, X., & Han, S. (2015). Challenging emotional prejudice by changing self-concept: priming independent self-construal reduces racial in-group bias in neural responses to other's pain. Soc Cogn Affect Neurosci, 10(9), 1195-1201. doi:10.1093/scan/nsv005

Watson, R., & de Gelder, B. (2017). How white and black bodies are perceived depends on what emotion is expressed. Sci Rep, 7(1), 1-12. doi: 10.1038/srep41349

Xu, X., Zuo, X., Wang, X., & Han, S. (2009). Do you feel my pain? Racial group membership modulates empathic neural responses. J Neurosci, 29(26), 8525-8529. doi:10.1523/jneurosci.2418-09.2009

Yan, Z., Schmidt, S. N. L., Saur, S., Kirsch, P., & Mier, D. (2019). The effect of ethnicity and team membership on face processing: a cultural neuroscience perspective. Soc Cogn Affect Neurosci, 14(9), 1017-1025. doi:10.1093/scan/nsz083

Zuo, X., & Han, S. (2013). Cultural experiences reduce racial bias in neural responses to others’ suffering. Culture and Brain, 1, 34-46.
